# Supplementary material for: Promoter‐pervasive transcription causes RNA polymerase II pausing to boost DOG1 expression in response to salt
Source: EMBO J. 2023 Jan 27;42(5):e112443. doi: 10.15252/embj.2022112443 (PMC9975946; doi:10.15252/embj.2022112443)
Supplement: Supplementary file 1 — Appendix [file EMBJ-42-e112443-s001.pdf]

Appendix for

Promoter-pervasive transcription causes Pol II pausing to boost *DOG1* expression in response to salt

Miguel Montez[1*], Maria Majchrowska[1], Michal Krzyszton[1], Grzegorz Bokota[2], Sebastian Sacharowski[1], Magdalena Wrona[1], Ruslan Yatusevich[1], Ferran Massana[1], Dariusz Plewczynski[2, 3] & Szymon Swiezewski[1*]

[1] Laboratory of Seeds Molecular Biology, Institute of Biochemistry and Biophysics, Polish Academy of Sciences, Pawinskiego 5a, 02-106, Warsaw, Poland.

[2] Laboratory of Functional and Structural Genomics, Centre of New Technologies, University of Warsaw, Banacha 2c, 02-097, Warsaw, Poland.

[3] Laboratory of Bioinformatics and Computational Genomics, Faculty of Mathematics and Information Science, Warsaw University of Technology, Koszykowa 75, 00-662, Warsaw, Poland.

[*] Co-corresponding authors

Table of Contents

Figure	Page
Appendix Figure S1	2
Appendix Figure S2	2
Appendix Figure S3	3
Appendix Figure S4	4
Appendix Figure S5	5
Appendix Figure S6	6

Appendix Figure S1

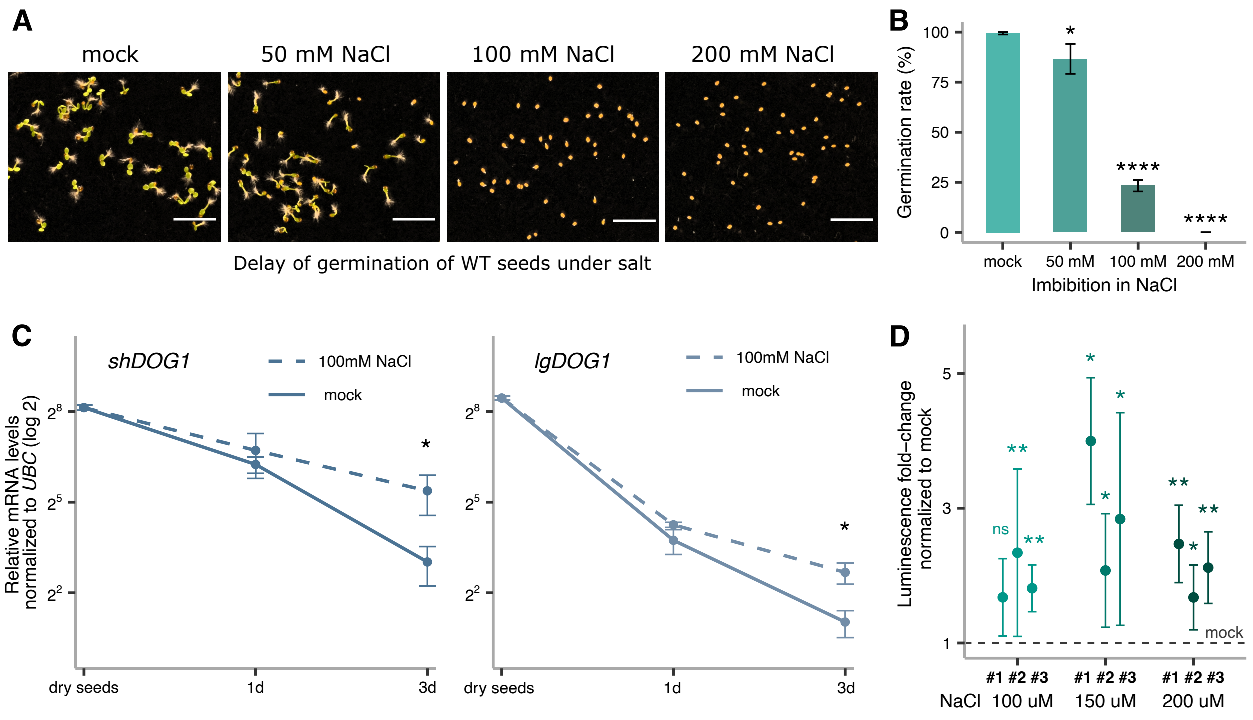

Appendix Figure S1 - *DOG1* expression and seed germination under salt stress

A Germination of WT seeds in different concentrations of NaCl and mock. Pictures were taken 3 days after stratification. Scale bars represent 5 mm.

B Quantification of germinated seeds 3 days after stratification. Bars and error bars represent the mean ± SD. * p-value < 0.05, **** p-value < 0.0001 from two-tailed Student's *t*-test.

C RT-qPCR relative quantification plots for *shDOG1* (left) and *lgDOG1* (right) normalized to *UBC21* (AT5G25760) in dry seeds and seeds imbibed for 1 day (1d) and 3 days (3d) in the absence or presence of 100 mM NaCl. Points and error bars represent the mean ± SD. * p-value < 0.05, **** p-value < 0.0001 from two-tailed Student's *t*-test.

D Luciferase reporter assay. The plot represents the luminescence fold-change of seeds under 100, 150, and 200 mM NaCl relative to mock (horizontal line) for 3 independent transgenic lines (#1, #2, #3) carrying the reporter construct *pDOG1-LUC::DOG1*. * p-value < 0.05, ** p-value < 0.01 from paired Student's *t*-test comparing the raw luminescence levels in counts per second between mock and NaCl-treated samples.

Appendix Figure S2

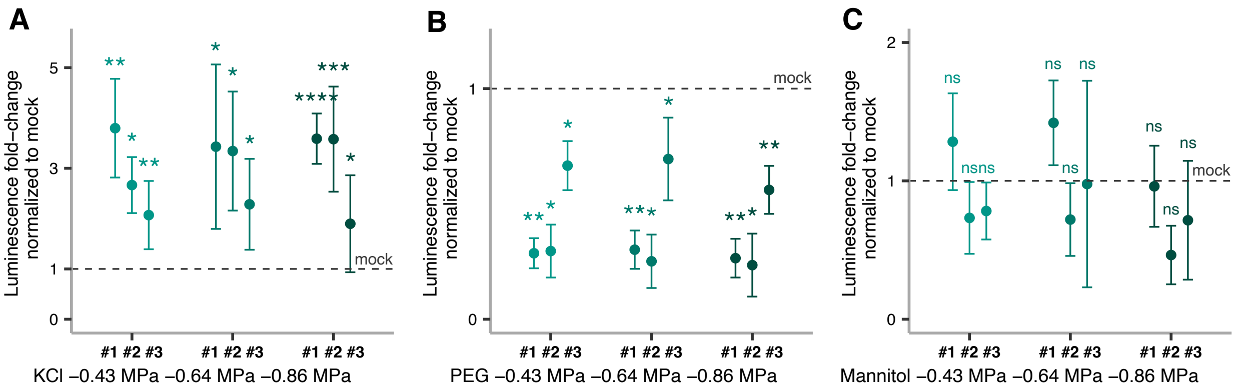

Appendix Figure S2 - Induction of *DOG1* gene expression upon ionic stress

A-C Luciferase reporter assays. Plots represent the changes in the luminescence of seeds caused by KCl (A), PEG (B), and mannitol (C), compared to mock (horizontal line). The osmotic pressures used are equivalent to the osmotic pressures caused by 100, 150, and 200 mM of NaCl. Three independent transgenic lines (#1, #2, #3) carrying the reporter construct *pDOG1-LUC::DOG1* were used. * p-value < 0.05, ** p-value < 0.01 from paired Student's *t*-test comparing the raw luminescence levels in counts per second between mock and treated samples.

Appendix Figure S3

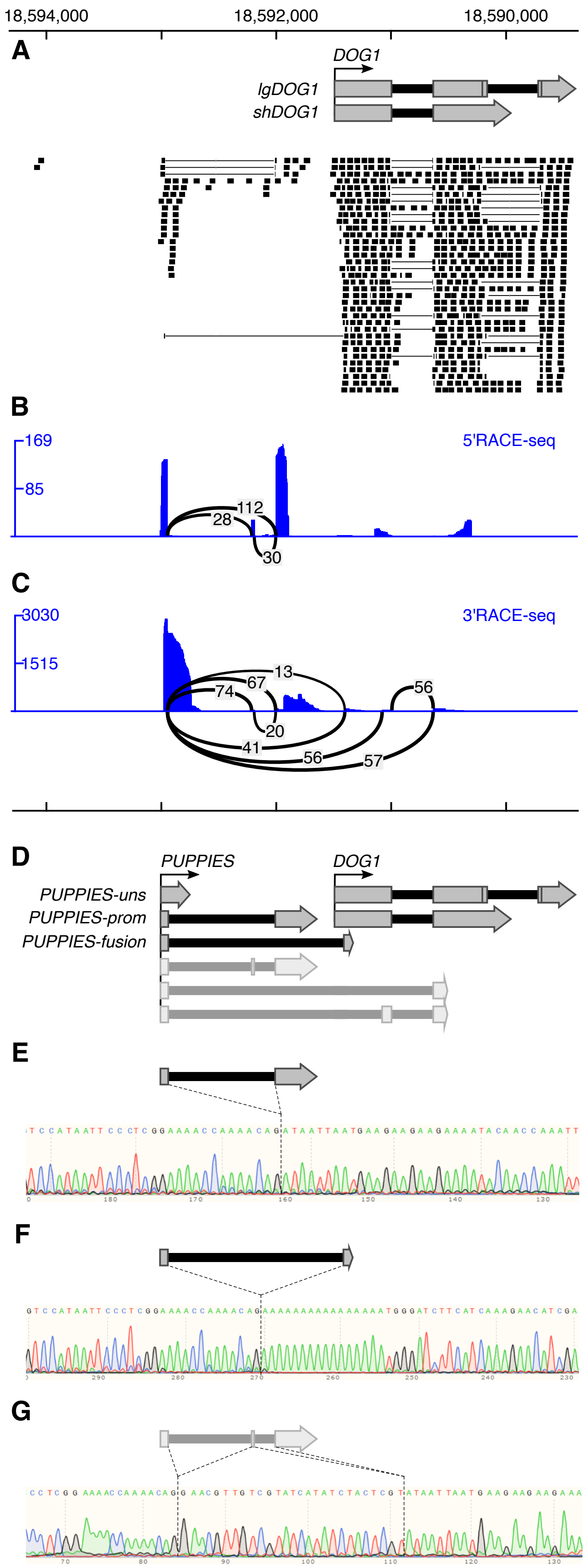

Appendix Figure S3 - Splicing of *PUPPIES* transcript isoforms

A Reads mapped to *DOG1 locus* from RNA-seq in seeds (Narsai *et al*., 2017), showing reads for the *PUPPIES* region. Above is shown a horizontal line giving the chromosome coordinates and a schematic representation of the annotated transcripts from the *DOG1 locus*.

B Sashimi plot from 5´RACE-seq.

C Sashimi plot from 3´RACE-seq.

D Schematic representation of all newly annotated *PUPPIES* transcripts based on our results from 3'RNA-seq, 5' and 3' RACE-seq. In grey are the *PUPPIES* isoforms not quantified using RT-qPCR.

E-G Sanger sequencing of products obtained by PCR amplification of cDNA using primers for *PUPPIES* region. Chromatograms showing splicing of *PUPPIES-prom* isoform (E), splicing of *PUPPIES-fusion* isoform (F), and alternative splicing of *PUPPIES-prom* isoform with the inclusion of a short alternative exon (G).

Appendix Figure S4

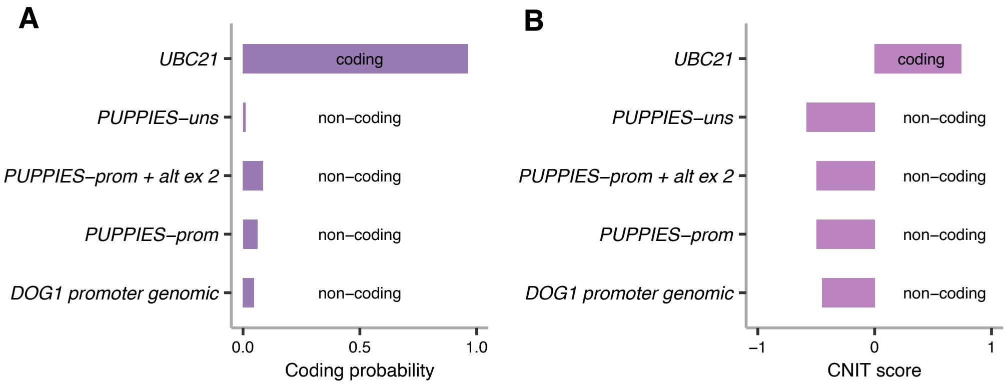

Appendix Figure S4 - Coding/non-coding potential of *PUPPIES* RNA transcripts

A Horizontal bar plot shows the coding probability from the online tool Coding Potential Calculator 2.0 (CPC 2.0; Kang *et al*., 2017).

B Horizontal bar plot shows the CNIT score for coding potential from the online tool Coding-NonCoding Identifying Tool (CNIT; Guo *et al*., 2019).

A, B Analyses were performed using the sequences of *PUPPIES-uns*, *PUPPIES-prom* with the inclusion of short alternative exon, *PUPPIES-prom*, the full genomic region of *DOG1* promoter, and *UBC21* as protein-coding gene control. Both CPC 2.0 and CNIT attribute a coding or non-coding label for each sequence analyzed as annotated on the plots.

Appendix Figure S5

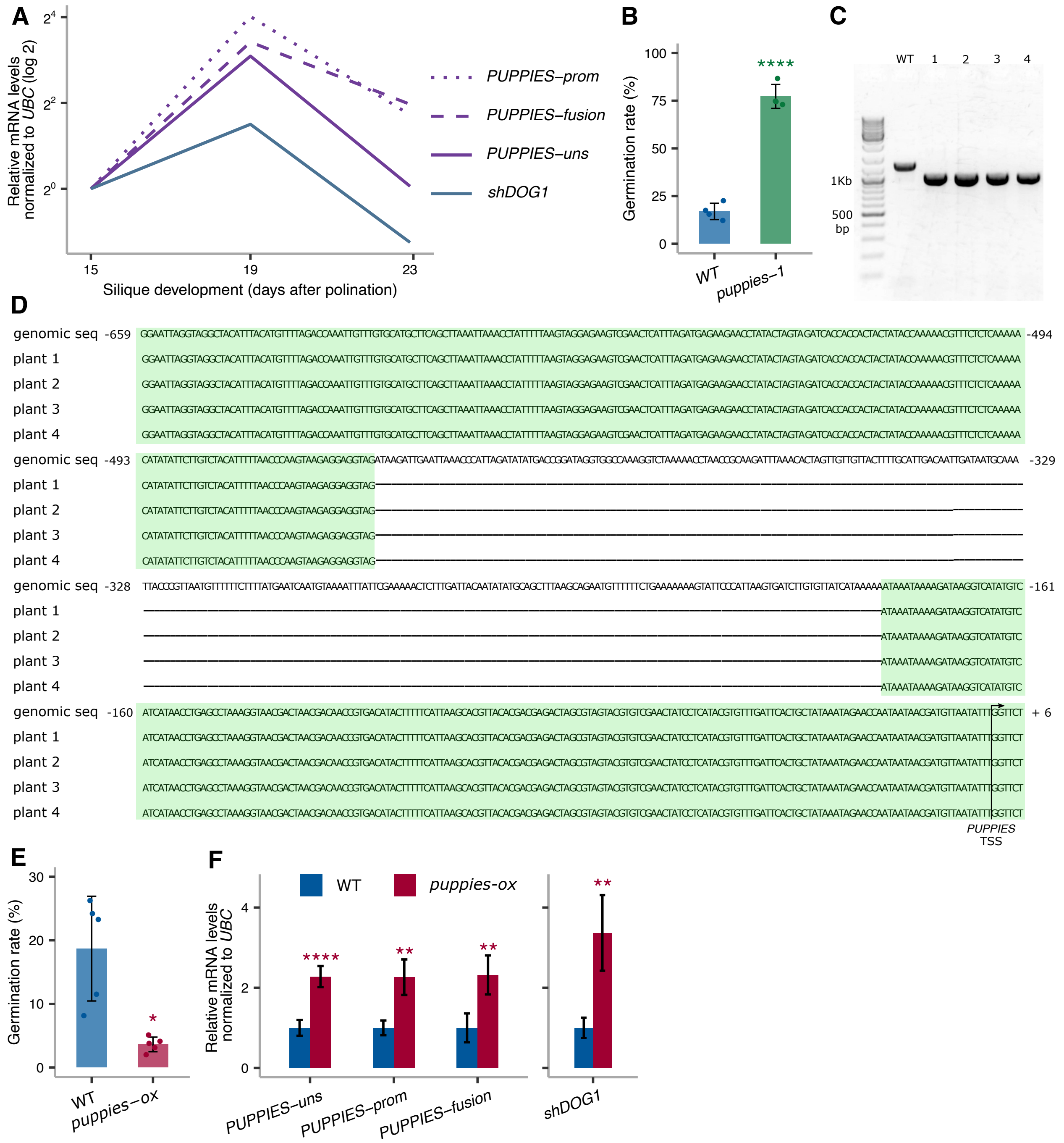

Appendix Figure S5 - *PUPPIES* contribution to primary dormancy strength

A Temporal expression patterns of *PUPPIES-prom*, *PUPPIES-fusion*, *PUPPIES-uns*, and *shDOG1*, during seed maturation (15, 19, and 23 days after pollination). RT-qPCR expression levels were normalized to *UBC21* and related to the first time point.

B Germination rate of seeds from WT and *puppies-1* one week after harvest (without stratification). Germination was counted 3 days after sowing.

C PCR amplification of genomic DNA from WT (lane 2) and 4 different T2 CRISPR-Cas9 plants (lanes 3 to 6) with primers flanking the deletion region. 1Kb Plus gel ladder (lane 1).

D Alignment of the results from Sanger sequencing of PCR products from 4 different T2 CRISPR-Cas9 plants to the genomic sequence of WT. Green boxes represent the sequences matching the genomic reference, while horizontal dashed lines represent the sequence deleted. Distance is given in bp as a relative to *PUPPIES* TSS (arrow).

E Germination rate of seeds from WT (blue) and *puppies-ox* (red) 3 weeks after harvest (without stratification).

B, E Bars and error bars represent the mean ± SD. Points represent individual biological replicates. * p-value < 0.05, **** p-value < 0.0001 from two-tailed Student's *t*-test.

F RT-qPCR expression fold-change of *PUPPIES-uns*, *PUPPIES-prom*, *PUPPIES-fusion*, and *shDOG1* in *puppies-ox* (red) relative to WT (blue) in maturing seeds from siliques 23 days after pollination. Bars and error bars represent the mean ± SD. ** p-value < 0.01, **** p-value < 0.0001 from two-tailed Student's *t*-test.

Appendix Figure S6

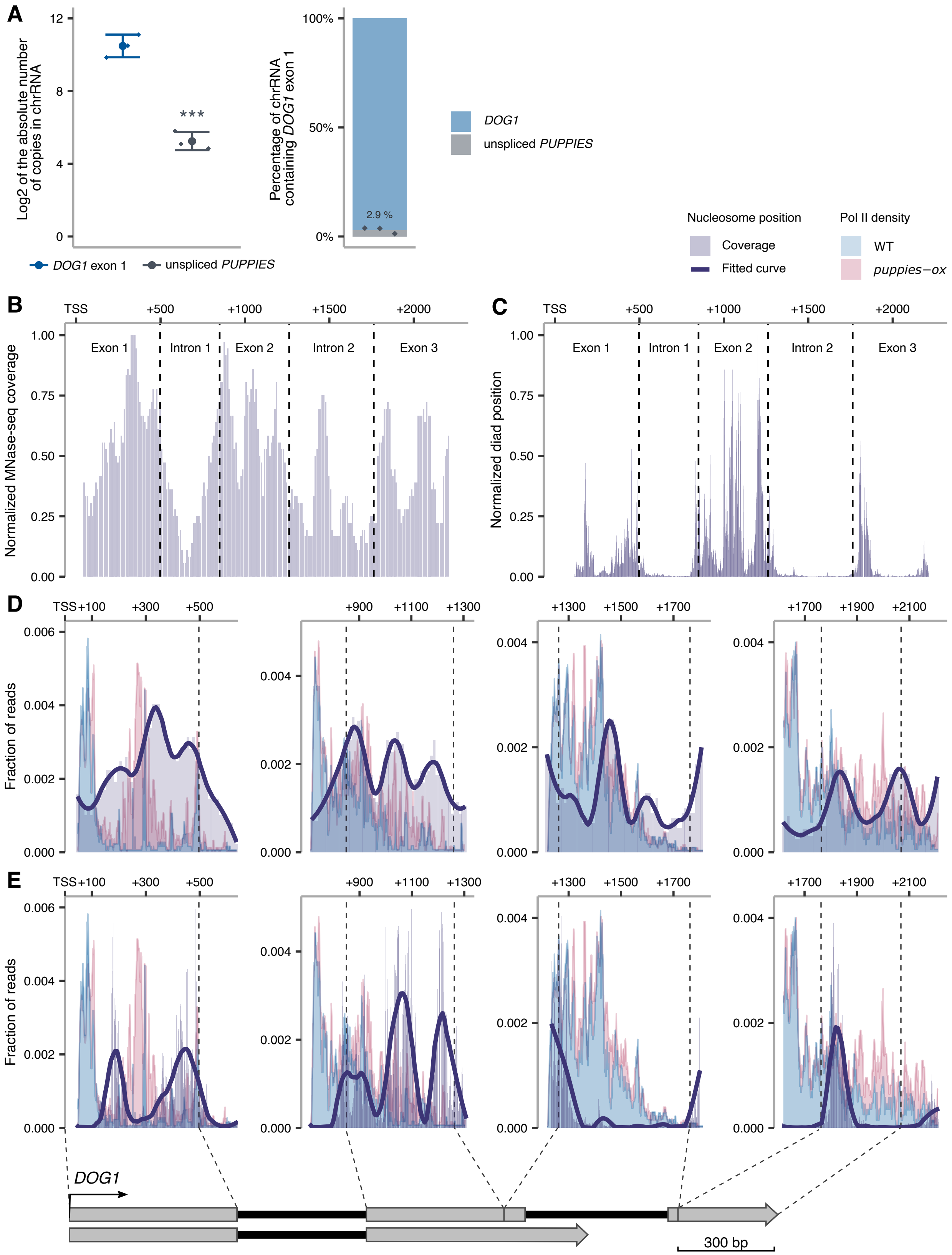

Appendix Figure S6 - Overlap of nucleosome position with Pol II profile on *DOG1 locus*

A The plot on the left shows the absolute levels (in log2 of the number of copies) of chromatin-attached unspliced *PUPPIES* and transcripts containing *DOG1* exon 1, in WT seeds under salt stress, measured by absolute RT-qPCR quantification. The plot on the right shows the levels of unspliced *PUPPIES* as the percentage of absolute *DOG1* exon 1 copy number. The *DOG1* levels (in light blue) are estimated by subtracting the number of copies of unspliced *PUPPIES* from the total *DOG1* exon 1, displayed in percentage.

B Plot representing read coverage from the MNase-seq dataset GSM4916341 (Data ref: Luo *et al*., 2020b) on the *DOG1 locus*.

C Plot representing nucleosome position prediction *in silico* based on genomic DNA sequence of the *DOG1 locus* (van der Heijden *et al*., 2012). Distance is given in bp from *DOG1* TSS. Vertical dashed lines show the exon boundaries, with text annotation for exons and introns.

D, E Overlap of targeted NET-Seq profiles of Pol II density for WT (blue) and *puppies-ox* (red) with nucleosome position from MNase-seq (Data ref: Luo *et al*., 2020b; D) and from *in silico* prediction (E). The nucleosome position is represented by purple lines and bars. A schematic of the *DOG1 locus* is given at the bottom with dashed lines connecting the exon boundaries with the plots. Dark grey boxes for exons of *lgDOG1* RNA isoform and light grey boxes for exons of *shDOG1* RNA isoform.
