# Supplementary material for: Promoter‐pervasive transcription causes RNA polymerase II pausing to boost DOG1 expression in response to salt
Source: EMBO J. 2023 Jan 27;42(5):e112443. doi: 10.15252/embj.2022112443 (PMC9975946; doi:10.15252/embj.2022112443)
Supplement: Supplementary file 2 — Expanded View Figures PDF [file EMBJ-42-e112443-s003.pdf]

Expanded View Figures

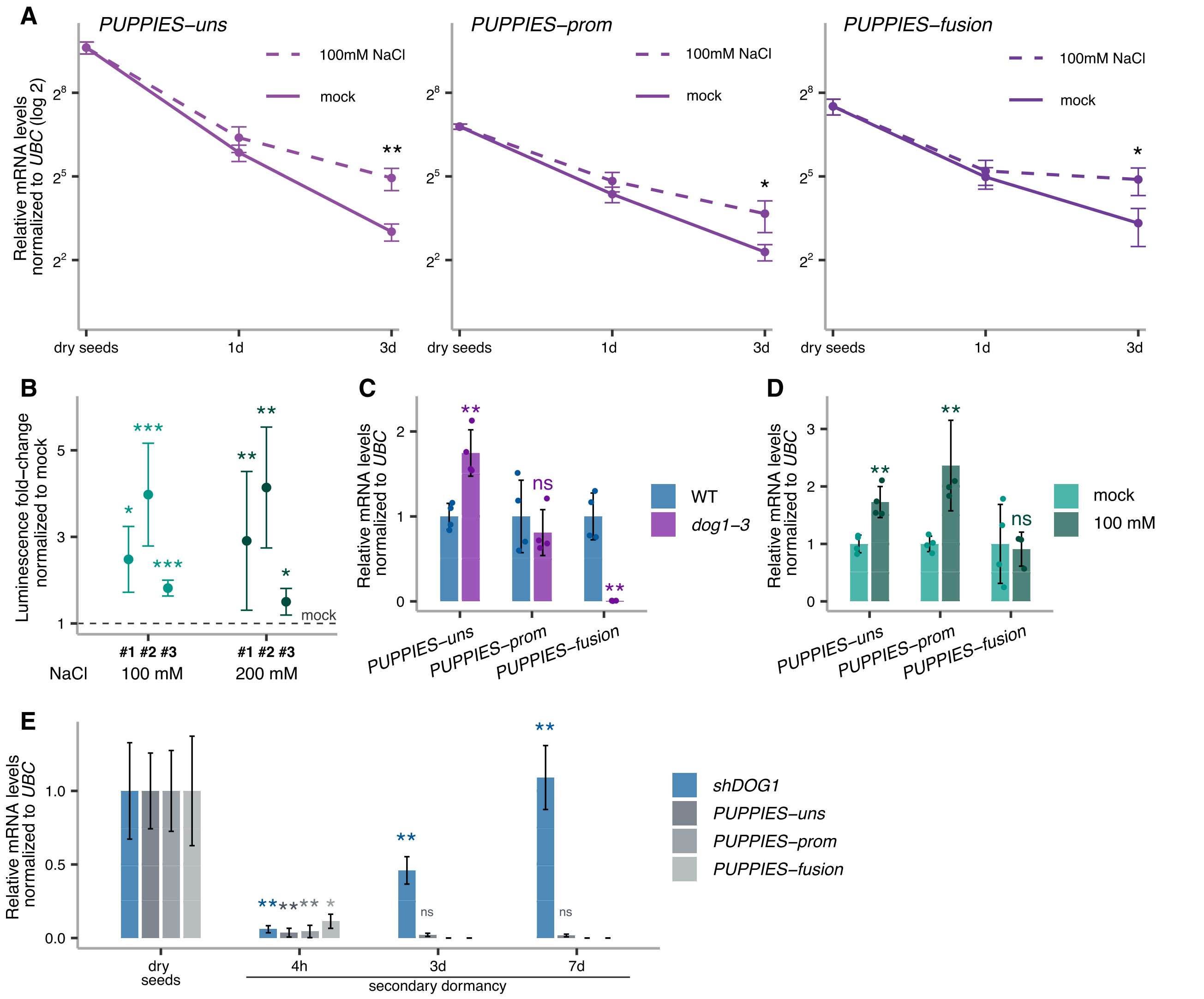

Figure EV1. *PUPPIES* response to salt stress.

A Relative quantification of *PUPPIES-uns*, *PUPPIES-prom* and *PUPPIES-fusion* (from left to right) normalized to *UBC21* in dry seeds and seeds imbibed for 1 and 3 days in mock versus 100 mM NaCl. RT–qPCR points and error bars represent the mean ± SD. **P*-value < 0.05, ***P*-value < 0.01 from two-tailed Student's *t*-test.

B Luciferase reporter assay. Plots represent the luminescence fold-change of seeds under 100 or 200 mM NaCl normalized to mock (horizontal dashed line) for three independent transgenic lines (#1, #2, #3) carrying the reporter construct *psDOG1::LUC*. Error bars represent the mean ± SD. **P*-value < 0.05, ***P*-value < 0.01, ****P*-value < 0.001 from paired Student's *t*-test comparing the raw luminescence levels in counts per second between mock and NaCl-treated samples.

C RT–qPCR for *PUPPIES-uns*, *PUPPIES-prom* and *PUPPIES-fusion*. Expression in seeds treated with 100 mM NaCl in *dog1-3* relative to WT.

D Expression in *dog1-3* mutant seeds treated with 100 mM NaCl relative to mock.

E RT–qPCR for *shDOG1*, *PUPPIES-uns*, *PUPPIES-prom* and *PUPPIES-fusion* during heat stress induction of secondary dormancy of WT seeds. Bars and error bars represent the mean ± SD with $n = 3$. Expression levels normalized to *UBC21*. Expression levels of *PUPPIES-prom* and *PUPPIES-fusion* at 3 and 7 days were undetectable. Asterisks show the statistical significance for each transcript from the two-tailed Student's *t*-test for the comparison of each timepoint to its previous timepoint. ns *P*-value > 0.05, **P*-value < 0.05, ***P*-value < 0.01.

Data information: (C, D) Expression levels normalized to *UBC21*. Bars and error bars represent the mean ± SD. Points represent biological replicates. ns *P*-value > 0.05, ***P*-value < 0.01 from two-tailed Student's *t*-test.

 © 2023 The Authors

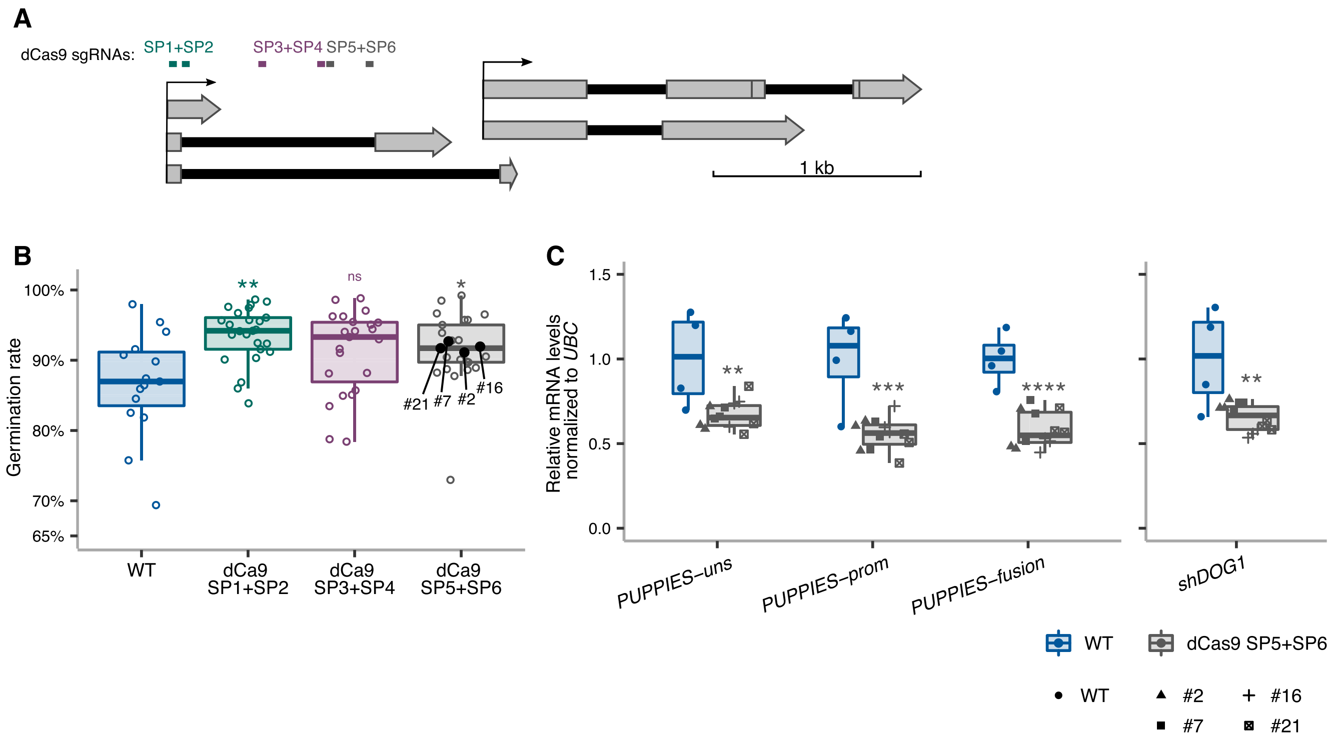

Figure EV2. Blocking *PUPPIES* with dCas9.

A Schematics of *DOG1 locus* with *PUPPIES* and *DOG1* TSS indicated by black arrows. Two sgRNAs per construct were introduced in WT targeting a ubiquitously expressed "dead" Cas9 protein (dCas9) to block *PUPPIES* transcription. The sgRNA positions are: SP1 + SP2 (in green), SP3 + SP4 (in purple), SP5 + SP6 (in grey).

B Box plots showing the percentage of germination in 150 mM NaCl at 4 days after stratification for WT (blue) and T2 seeds from selected transformants carrying dCas9 and the sgRNA pairs SP1 + SP2 (green), SP3 + SP4 (purple), SP5 + SP6 (grey). Points show the germination of seeds from each individual transgenic plant. Four independent SP5 + SP6 plants (#2, #7, #16, #21, represented by black points) with germination closest to the median were propagated and gene expression analyses (C) were carried out in the T3 seeds imbibed in the presence of 100 mM NaCl. Points represent biological replicates.

C Box plots show the expression levels measured by RT–qPCR of *PUPPIES-uns*, *PUPPIES-prom*, *PUPPIES-fusion* and *shDOG1* normalized to *UBC21* in the four different transgenic lines of SP5 + SP6 dCas9 (grey) relative to WT (blue). Points represent biological replicates.

Data information: (B, C) The box plot's central band marks the median, boxes mark the first and third quartiles, and whiskers extend the boxes to the largest value no further than 1.5 times the interquartile range. Statistical significance from two-tailed Student's *t*-test. ns *P*-value > 0.05, **P*-value < 0.05, ***P*-value < 0.01, ****P*-value < 0.001, *****P*-value < 0.0001.

© 2023 The Authors

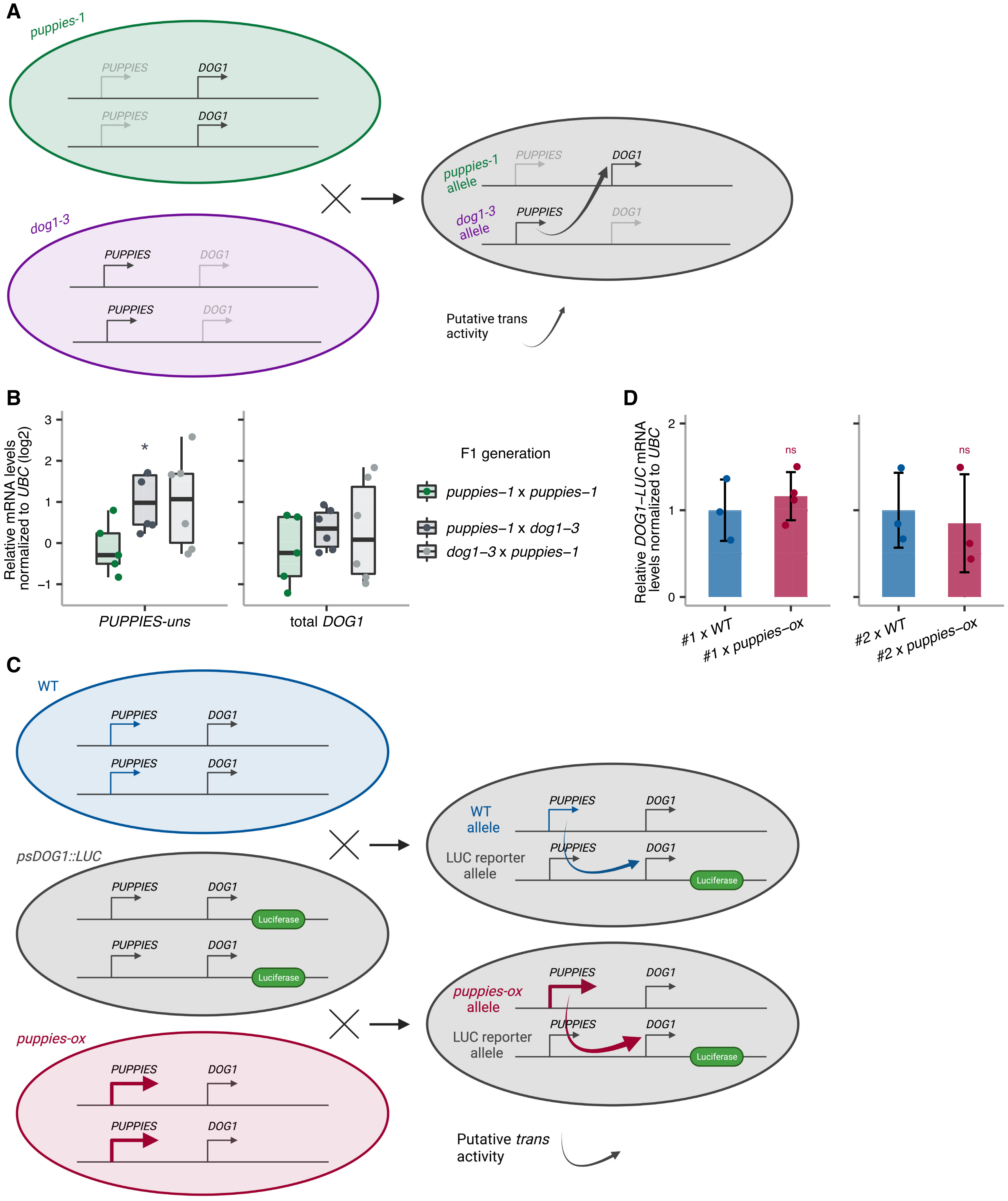

Figure EV3.

© 2023 The Authors

Figure EV3. *PUPPIES* do not activate *DOG1* expression in *trans*.

A Schematic representation of the cross between both diploid homozygous *puppies-1* and *dog1-3* mutants. The heterozygous F1 is used to test if supplying *PUPPIES* expression (from the *dog1-3* allele) induces *DOG1* expression (from the *puppies-1* allele) compared with the homozygous *puppies-1*.

B RT–qPCR for *PUPPIES-uns* and total *DOG1* normalized to *UBC21* in F1 seeds imbibed in the presence of 100 mM NaCl. *y*-axis in logarithmic scale. Points represent biological replicates. The box plot's central band marks the median, boxes mark the first and third quartiles, and whiskers extend the boxes to the largest value no further than 1.5 times the interquartile range. Statistical significance from two-tailed Student's *t*-test. *P-value < 0.05.

C Schematic representation of the crosses between *psDOG1::LUC* and either WT or *puppies-ox*. The heterozygous F1 from *psDOG1::LUC* crossed with *puppies-ox* is used to test if supplying *PUPPIES* expression (from the *puppies-ox* allele) induces *DOG1-LUC* expression (from the reporter allele) relative to the levels of *DOG1-LUC* in the *psDOG1::LUC* crossed with WT.

D RT–qPCR with primers specific for *DOG1-LUC* in F1 seeds of two independent *psDOG1::LUC* lines (#1 and #2) crossed with either WT or *puppies-ox* imbibed in the presence of 100 mM NaCl. Expression was normalized to *UBC21*. Bars and error bars represent the mean ± SD. Points represent biological replicates. ns P-value > 0.05 from two-tailed Student's *t*-test.

Figure EV4. *DOG1* single-molecule RNA FISH.

A Single *z*-section image of smFISH for *DOG1* in embryo cells from imbibed seeds. Separate DAPI (blue) and *DOG1* (grey) channels are shown on the left and middle images. A merged image is shown on the right. Manual segmentation (green outline) of the cells is performed based on a certain level of background fluorescence visible on the *DOG1* channel. Arrowhead points to the *focus* corresponding to the transcription site (TS). For each cell, the segmented area is projected to the *z*-sections above and below corresponding, respectively, to the top and bottom edges of the cell. Scale bar is 5 μm.

B After the projection of the cell segmentation across the *z*-stack, the image is denoised in each *z*-section. A maximum projection of the denoised *z*-stack is then performed to obtain a representative 2D image (C). The denoised *z*-stack is also used for the reconstruction of the cell and *foci* detection in 3D (see Materials and Methods section).

C Arrowhead points to the *focus* corresponding to the TS. Scale bar is 2 μm.

D, E *z*-stack max-projection images of *DOG1* smFISH in seeds imbibed in 100 mM NaCl of WT (left) versus *dog1-3* (right). The scale bar is 5 μm. Please note that WT picture in panel E is the same representative picture as used in Fig 4G.

F *z*-stack max-projection images of *DOG1* smFISH in WT without (left) versus with (right) RNase A treatment before hybridization. The scale bar is 5 μm.

G *z*-stack max-projection images of *DOG1* smFISH in seeds imbibed in 100 mM NaCl of WT (left) versus *puppies-1* (right). The scale bar is 5 μm.

H Comparison of fold-change of *DOG1* expression from RT–qPCR (left) with cytoplasmic *DOG1 foci* number per cell from smFISH (right; replotted data shown in Fig 4E) in *puppies-1* relative to WT. Points and error bars represent the mean ± SD. Diamond-shaped points in the RT–qPCR plot represent a pool of seeds collected from five plants, and diamond-shaped points in the smFISH plot represent a single embryo. Statistical significance from two-tailed Student's *t*-test. *P-value < 0.05, ****P-value < 0.0001.

I Distribution of intensities of cytoplasmic *DOG1 foci* in WT and *puppies-1* in arbitrary units (a.u.).

J *z*-stack max-projection images of *DOG1* smFISH in seeds imbibed in 100 mM NaCl of WT (left) versus *puppies-ox* (right). The scale bar is 5 μm.

K RT–qPCR quantification of 3′ end and 5′ end of *shDOG1* on nascent RNA from seeds imbibed in 100 mM NaCl from WT, *puppies-ox* and *puppies-1*. The plot shows the ratio of 3′ end to 5′ end relative to WT. Nascent RNA levels were normalized to *UBC21*. Bars and error bars represent the mean ± SD. Statistical significance from two-tailed Student's *t*-test. ns P-value > 0.05.

© 2023 The Authors

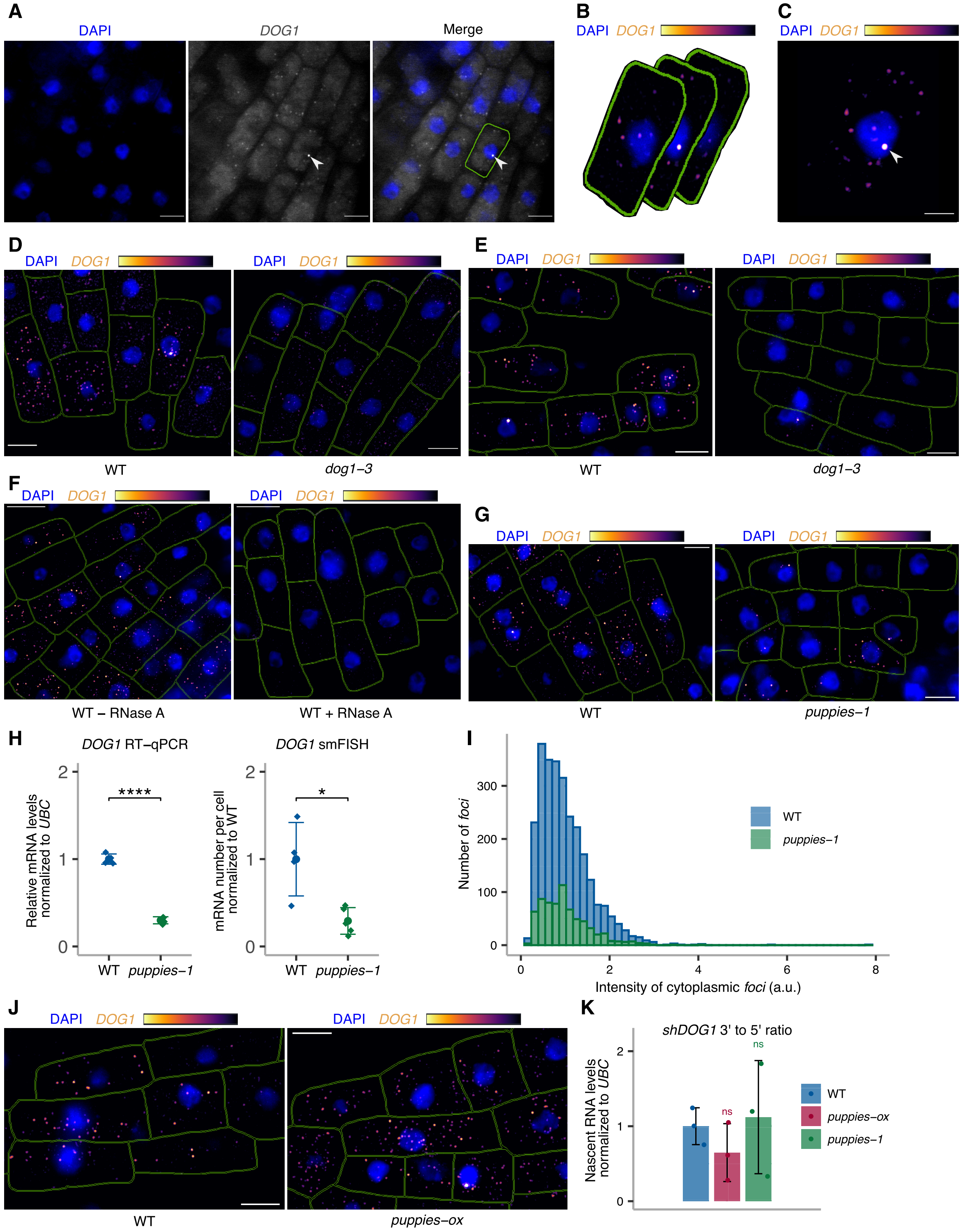

Figure EV4.

© 2023 The Authors

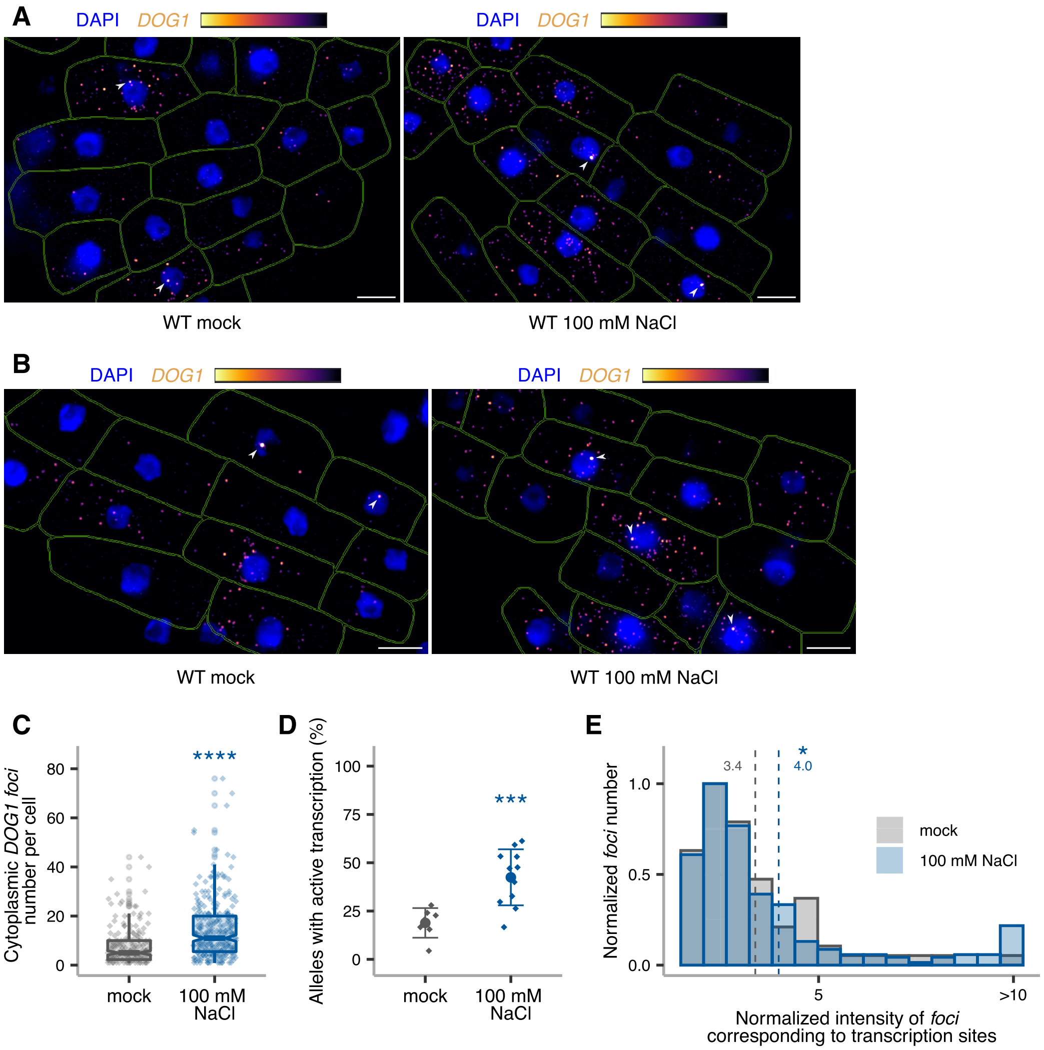

Figure EV5. *DOG1* single-molecule RNA FISH in response to salt stress.

A, B *z*-stack max-projection images of *DOG1* smFISH in WT embryos from imbibed seeds in mock (left) versus 100 mM NaCl (right). Arrowheads point to *foci* corresponding to the transcription sites (TS). The scale bar is 5 μm.

C Notched box plots showing the cytoplasmic *DOG1 foci* number per cell in mock versus salt treatment. Diamond-shaped points represent each cell, $n = 208$ cells from mock and $n = 309$ cells from NaCl-treated. The box plot's central band marks the median, lower and upper box limits mark the first and third quartiles, whiskers extend the boxes to the largest value no further than 1.5 times the interquartile range, and the notches extend to 1.58 times the interquartile range divided by sqrt (n).

D Plot showing the frequency of alleles with detected *foci* corresponding to *DOG1* transcription sites for mock versus NaCl-treated. Points and error bars represent the mean ± SD. Diamond-shaped points represent single embryos.

E Distribution of intensities of *foci* corresponding to *DOG1* TS in mock and NaCl-treated. In the *x*-axis is the fluorescence intensity fold-change of *foci* classified as TS to the average intensity of nuclear *foci*. In the *y*-axis is the number of *foci* normalized to their maximum value. Vertical dashed lines indicate the average fluorescence fold-change for mock in green (3.4) and NaCl-treated in blue (4.0). $n = 47$ *foci* from mock and $n = 159$ *foci* from NaCl-treated.

Data information: (C–E) Statistical significance from two-tailed Student's *t*-test. *P-value < 0.05, ***P-value < 0.001, ****P-value < 0.0001.

© 2023 The Authors
