# Supplementary material for: Promoter‐pervasive transcription causes RNA polymerase II pausing to boost DOG1 expression in response to salt
Source: EMBO J. 2023 Jan 27;42(5):e112443. doi: 10.15252/embj.2022112443 (PMC9975946; doi:10.15252/embj.2022112443)
Supplement: Supplementary file 8 — PDF+ [file EMBJ-42-e112443-s004.pdf]

Article

TRANSPARENT PROCESS OPEN ACCESS

THE EMBO JOURNAL

# Promoter-pervasive transcription causes RNA polymerase II pausing to boost *DOG1* expression in response to salt

Miguel Montez[1,*], Maria Majchrowska[1], Michal Krzyszton[1], Grzegorz Bokota[2], Sebastian Sacharowski[1], Magdalena Wrona[1], Ruslan Yatusevich[1], Ferran Massana[1], Dariusz Plewczynski[2,3] & Szymon Swiezewski[1,**]

## Abstract

**Eukaryotic genomes are pervasively transcribed by RNA polymerase II. Yet, the molecular and biological implications of such a phenomenon are still largely puzzling. Here, we describe noncoding RNA transcription upstream of the *Arabidopsis thaliana DOG1* gene, which governs salt stress responses and is a key regulator of seed dormancy. We find that expression of the *DOG1* gene is induced by salt stress, thereby causing a delay in seed germination. We uncover extensive transcriptional activity on the promoter of the *DOG1* gene, which produces a variety of lncRNAs. These lncRNAs, named *PUPPIES*, are co-directionally transcribed and extend into the *DOG1* coding region. We show that *PUPPIES* RNAs respond to salt stress and boost *DOG1* expression, resulting in delayed germination. This positive role of pervasive *PUPPIES* transcription on *DOG1* gene expression is associated with augmented pausing of RNA polymerase II, slower transcription and higher transcriptional burst size. These findings highlight the positive role of upstream co-directional transcription in controlling transcriptional dynamics of downstream genes.**

**Keywords** long noncoding RNAs; RNA polymerase II pausing; salt stress; transcriptional dynamics
**Subject Categories** Chromatin, Transcription & Genomics; Plant Biology
**DOI** 10.15252/embj.2022112443 | Received 23 August 2022 | Revised 2 January 2023 | Accepted 9 January 2023 | Published online 27 January 2023
**The EMBO Journal (2023) 42: e112443**

## Introduction

Seed germination is a major phase transition in the plant's development. It is influenced by seed dormancy, which is the ability of seeds to postpone germination when under favourable conditions. Seed dormancy contributes to the natural variability of germination timing and bet-hedging (Finch-Savage & Footitt, 2017). *Delay of germination 1* (*DOG1*) gene is a QTL for seed dormancy (Alonso-Blanco *et al*, 2003; Bentsink *et al*, 2006). *DOG1* gene expression is induced during seed maturation and accumulated DOG1 protein results in the dormancy of mature dry seeds (Nakabayashi *et al*, 2012).

*DOG1* expression is regulated at the transcriptional level by multiple mechanisms, including alternative polyadenylation (APA) generating short *DOG1* (*shDOG1*) and long *DOG1* (*lgDOG1*) isoforms (Cyrek *et al*, 2015), as well as repressed by the antisense long noncoding RNA (lncRNA) *1GOD* (Fedak *et al*, 2016; Kowalczyk *et al*, 2017).

In plants, antisense lncRNAs are typically transcribed from promoters within the 3′ or downstream gene regions. Besides *1GOD*, antisense lncRNAs have been described to control important physiological responses (Swiezewski *et al*, 2009; Kindgren *et al*, 2018; Zhao *et al*, 2018). Upstream of genes, another class of noncoding transcription, gives rise to promoter upstream transcripts (PROMPTS). PROMPTS are frequently transcribed in the antisense orientation and quickly degraded by the exosome (Lloret-Llinares *et al*, 2016; Thieffry *et al*, 2020). Additionally, promoter regions are also a source of stable lncRNA transcribed as independent transcriptional units including *APOLO* and *COLDWRAP*, with a repressive impact on the nearby genes (Ariel *et al*, 2014; Kim & Sung, 2017). Furthermore, promoter regions can also be transcribed as a result of readthrough from the upstream gene or an upstream transcription start site (TSS) of the same gene. In the yeast model *S. cerevisiae*, pervasive transcription from the upstream gene over the downstream was observed in one-quarter of all tandem genes (Pelechano *et al*, 2013). A similar abundance of such events was also observed in human cells (Vilborg *et al*, 2015; Vilborg & Steitz, 2017). In *Arabidopsis*, the readthrough of upstream transcripts over downstream genes was shown to be limited by the activity of nuclear exoribonucleases (Crisp *et al*, 2018; Krzyszton *et al*, 2018) and BORDER proteins (Yu *et al*, 2019).

1 Laboratory of Seeds Molecular Biology, Institute of Biochemistry and Biophysics, Polish Academy of Sciences, Warsaw, Poland
2 Laboratory of Functional and Structural Genomics, Centre of New Technologies, University of Warsaw, Warsaw, Poland
3 Laboratory of Bioinformatics and Computational Genomics, Faculty of Mathematics and Information Science, Warsaw University of Technology, Warsaw, Poland
*Corresponding author. Tel: +48 22 592 57 25; E-mail: miguel-coelho@hotmail.com
**Corresponding author. Tel: +48 22 592 57 25; E-mail: sswiez@ibb.waw.pl

© 2023 The Authors. Published under the terms of the CC BY 4.0 license

Here, we uncover the role of *DOG1* in controlling the timing of germination of seeds under salt stress. In response to this stress, the *DOG1* gene promoter is extensively transcribed, generating a variety of lncRNAs, that we name *PUPPIES*. We show that *PUPPIES* pervasive transcription, induced in response to salt stress, stimulates *DOG1* expression to delay germination. Our results indicate that *PUPPIES* induce *DOG1* expression by altering Pol II dynamics on the gene. *PUPPIES* boost the number of Pol II molecules loaded per *DOG1* transcriptional burst. Interestingly, the enhanced *DOG1* transcription is accompanied by augmented Pol II pausing, slower transcription through nucleosome-containing but not nucleosome-depleted regions of *DOG1*, together with more efficient splicing.

# Results

### *DOG1* gene regulates the speed of seed germination upon salt stress

Understanding seed dormancy is crucial for agriculture as farmers strive for rapid and uniform germination that synchronizes plant development and reduces costs. Seeds rarely face optimal germination conditions, and salt stress is known to delay seed germination in various plant species (Abel & MacKenzie, 1964; Ungar, 1978; Jones, 1986), including *Arabidopsis* (Quesada *et al*, 2002; Vallejo *et al*, 2010). In agreement, we show that the delay of germination of wild-type (WT) seeds is proportional to the increasing concentrations of NaCl (Appendix Fig S1A and B). Given the key role of the *DOG1* gene in seed dormancy-mediated control of germination time, we tested whether *DOG1* also takes part in delaying seedling establishment under salt stress.

We show that *DOG1* knockout (*dog1-3*) and upregulation (*dog1-5*) mutants (Cyrek *et al*, 2015; Fedak *et al*, 2016) display, respectively, weaker and stronger inhibition of germination by various concentrations of NaCl (Fig 1A). Analysis of the germination time curve in the presence of 100 mM NaCl (Fig 1B) suggests that *dog1-3* and *dog1-5* mutants have, respectively, faster and slower germination under salt stress. As reported during seed maturation (Fedak *et al*, 2016), we confirm that in seeds imbibed in 100 mM NaCl, *dog1-3* and *dog1-5* mutants have, respectively, lower and higher *DOG1* expression relative to WT (Fig 1C). These results suggest that *DOG1* gene is involved in the physiological response of seeds to salt stress. In our experiments, we applied salt stress during stratification, which releases seed dormancy and leads to no visible differences in germination speed between WT and *dog1* mutants in the absence of salt (Fig 1A). This suggests that the role of the *DOG1* gene in the salt-induced delay of germination is independent of its function in primary dormancy.

RT–qPCR reveals that after 3 days of imbibition in the presence of NaCl, *DOG1* expression is significantly induced relative to mock (Fig 1D and Appendix Fig S1C). We confirmed this using transgenic lines with a luciferase (*LUC*) reporter inserted after the start codon in *DOG1* genomic sequence (*pDOG1-LUC::DOG1*; Fedak *et al*, 2016; Fig 1E and F). Three independent lines show increased luminescence when exposed to NaCl (Appendix Fig S1D), confirming that *DOG1* expression is induced by salt. Salt stress imposes a combination of augmented osmotic stress and ionic toxicity. Interestingly, the *pDOG1-LUC::DOG1* signal was induced by NaCl (Fig 1F and Appendix Fig S1D) and KCl (Appendix Fig S2A) but not by PEG or mannitol (Appendix Fig S2B and C). These results suggest that *DOG1* expression is induced by ionic stress but not osmotic stress. In summary, our results indicate that *DOG1* apart from its well-known function in seed dormancy is induced in seeds in response to ionic imbalance and plays a role in controlling the speed of germination under salt stress.

### Transcriptomic response to salt stress in seeds depends on *DOG1* expression

In order to investigate the underlying genome-wide salt stress response and its dependence on *DOG1*, we performed 3'RNA-seq in imbibed seeds from WT and *DOG1* knockout *dog1-3* in the presence and absence of 100 mM NaCl. Salt treatment of WT seeds results in 835 downregulated and 1,136 upregulated genes ($FDR < 0.05$ and $|\log2FC| > \log2(1.5)$; Fig 1G and Dataset EV1). Downregulated genes are overrepresented for nonredundant gene ontology (GO) terms associated with biosynthetic and metabolic processes (Fig 1H and Dataset EV2). Upregulated genes are overrepresented for nonredundant GO terms associated with stress responses, including water deprivation (Fig 1I and Dataset EV2). These results are consistent with published transcriptomic data from salt stress (Sun *et al*, 2019; Dorone *et al*, 2021; Wang *et al*, 2021; Butt *et al*, 2022) and provide a baseline for further studies of stress response in seeds. Additionally, we observe that *DOG1* and multiple other regulators of seed germination are differentially expressed upon salt treatment (Fig 1J). This explains the changes in germination under the stress and suggests it does not solely depend on *DOG1* but a combination of multiple regulators.

Interestingly, only 49% of differentially expressed genes (DEGs) identified in salt-treated WT seeds are differentially expressed in *dog1-3* upon salt treatment (Fig 1K). Notably, we observe that genes induced by salt in WT are generally induced to a lesser extent in the *dog1-3* mutant, many of those being over two times less upregulated or even downregulated (Fig 1L). Similarly, genes repressed in salt-treated WT seeds are less down or even upregulated in the *dog1-3* mutant (Fig 1M). These results show that the *dog1-3* mutant has an altered transcriptomic response to salt stress.

### *DOG1* gene promoter is pervasively transcribed

Inspection of 3'RNA-seq reveals a surprisingly high coverage of reads mapped to the *DOG1* promoter and part of exon 1 in salt conditions (Fig 2A), suggesting the existence of previously unannotated sense transcripts in the *DOG1* promoter. Of note, reads over this region are also detected in a publicly available RNA-seq dataset from *Arabidopsis* seeds (Narsai *et al*, 2017; Appendix Fig S3A). Using a nanoCAGE- and nanoPARE-based (Salimullah *et al*, 2011; Schon *et al*, 2018) 5'RACE-seq, we confirm transcription of *DOG1* promoter originating from a well-defined TSS around 1.5-Kb upstream of *DOG1* TSS (Fig 2B). 3'RACE-seq shows that in contrast to the 5′ end, the 3′ ends of this lncRNA are not well defined, giving rise to multiple transcript isoforms (Fig 2C). We named these transcripts *PUPPIES* due to their proximity to the *DOG1* gene. *PUPPIES* can terminate shortly after their TSS generating unspliced transcripts around 260 bp long (*PUPPIES-uns*); *PUPPIES* can also be spliced into and terminate over a broad region on the *DOG1*

 © 2023 The Authors

promoter (*PUPPIES-prom*) or can be spliced into the *DOG1* coding region terminating inside *DOG1* exons 1 and 2 (*PUPPIES-fusion*; Fig 2D and Appendix Fig S3B–D). The splicing events were confirmed by PCR followed by Sanger sequencing (Appendix Fig S3E and F). Additionally, as 3'RNA-seq is based on oligo(dT) priming, at least some *PUPPIES* isoforms are likely to be polyadenylated. The coding/noncoding potential of *PUPPIES* transcripts was assessed using Coding Potential Calculator 2.0 (CPC 2.0; Kang *et al*, 2017) and Coding-NonCoding Identifying Tool (CNIT; Guo *et al*, 2019). Both tools classify all tested *PUPPIES* isoforms as noncoding in contrast to the coding control *UBC21* (Appendix Fig S4A and B).

Here, we show that in seeds imbibed under salt stress, the *DOG1* promoter region is extensively transcribed generating lncRNAs named *PUPPIES*. *PUPPIES* are a collection of diverse isoforms generated by alternative splicing and termination. Besides, *PUPPIES* are co-directionally transcribed and invasive to the *DOG1* promoter and gene body.

### *PUPPIES* transcription is responsive to salt stress

Our RNA-seq data suggest that *PUPPIES* are induced by salt stress (Fig 2A). To confirm these results, we performed RT–qPCR in seeds

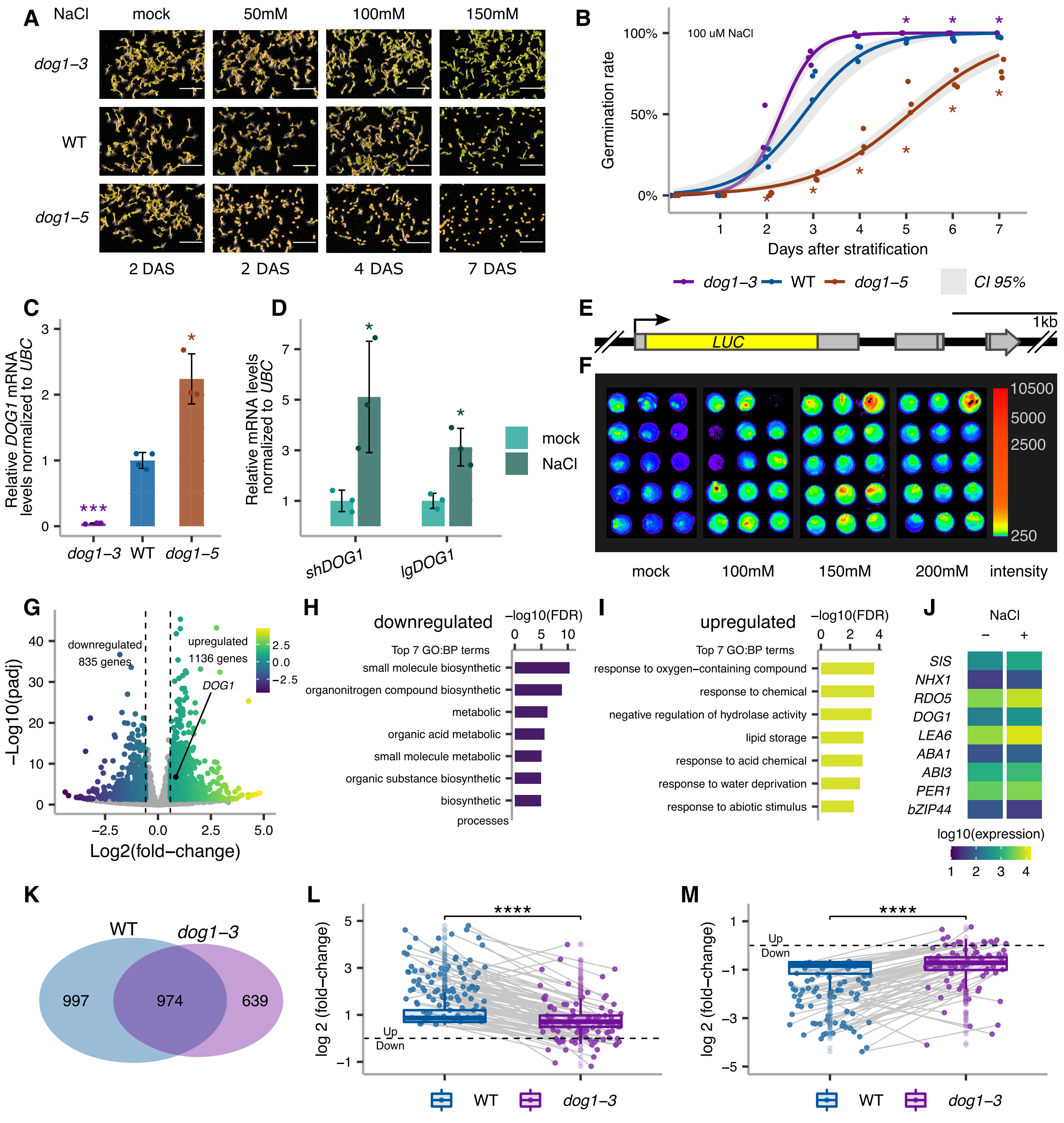


**Figure 1.**

**Figure 1. Seed phenotypic and transcriptomic response to salt stress depends on *DOG1*.**

A Germination of *dog1-3* (knockout), WT and *dog1-5* (*DOG1* upregulation) seeds in media supplemented with NaCl for the indicated number of days after stratification (DAS). Scale bar represents 5 mm.

B Germination rate in 100 mM NaCl measured during 7 days after stratification for seeds of different genotypes. Lines represent the fitted curves with a 95% confidence interval (grey area). *$P$-value $< 0.05$ from two-tailed Student's $t$-test. Data for WT are the same as plotted in Fig 3C.

C *shDOG1* expression levels normalized to *UBC21* (AT5G25760) in *dog1-3* and *dog1-5* relative to WT.

D *shDOG1* and *lgDOG1* expression levels normalized to *UBC21* in seeds treated with 100 mM NaCl relative to mock.

E Schematics of the *pDOG1-LUC::DOG1* construct with *LUC* reporter inserted after *DOG1* ATG, including complete intergenic regions upstream and downstream of the *DOG1* gene (Fedak *et al*, 2016).

F Luciferase reporter activity in seeds in different concentrations of NaCl or mock. The colour bar shows the luminescence in counts per second on a logarithmic scale.

G Volcano plot shows DEGs as coloured points (DESeq2; |log2fold-change| > log2(1.5), FDR < 0.05). The colour scale shows log2fold-change. *DOG1* gene is highlighted in black. The number of downregulated and upregulated genes is provided on the plot.

H, I Top 7 nonredundant Gene Ontology terms for biological process (GO:BP) for down- (H) and upregulated genes (I).

J Mean absolute expression levels from 3'RNA-seq of selected genes known to be involved in salt stress response and/or seed germination. Data from mock (−) and 100 mM NaCl (+).

K Venn diagram shows the overlap between genes affected by salt treatment in WT and *dog1-3* (DESeq2; |log2fold-change| > log2(1.5), FDR < 0.05).

L, M Box plots represent the behaviour of genes upregulated (L) and downregulated (M) by salt in WT. Points show genes for which expression is more than two times higher or lower in *dog1-3* compared with WT. Grey lines connect the same genes. The box plot's central band marks the median, boxes mark the first and third quartiles, and whiskers extend the boxes to the largest value no further than 1.5 times the interquartile range. For the comparison of transcriptomic responses, the Wilcoxon rank-sum test was applied using each gene (DEG in WT) as a biological replicate. Sample size $n$ = 1,136 for both WT and *dog1-3* (L) and $n$ = 835 for both WT and *dog1-3* (M) ****$P$-value $< 0.0001$.

Data information: (C, D) Bars and error bars represent the mean ± SD. Points represent biological replicates. *$P$-value $< 0.05$, ***$P$-value $< 0.001$ from two-tailed Student's $t$-test.

imbibed in the absence or presence of NaCl using primers specific for three *PUPPIES* isoforms: *PUPPIES-uns*, *PUPPIES-prom* and *PUPPIES-fusion* (Fig 2D). We show that salt stress not only induces *DOG1* but also all tested *PUPPIES* isoforms (Figs 2E and EV1A), confirming the initial observation from 3'RNA-seq.

Next, we used a published transgenic line (Fedak *et al*, 2016) with the *DOG1* promoter region (containing *PUPPIES*) followed by the *DOG1* exon1, intron1 and exon2 region fused to luciferase reporter (Fig 2F). This construct lacks the 3′ end of the *DOG1* gene which contains a previously characterized antisense transcript *1GOD* (Fedak *et al*, 2016) as well as *DOG1* exon 3 of the *lgDOG1* isoforms (Cyrek *et al*, 2015). Using three independent transgenic lines, we show that this truncated *DOG1* construct is still induced by salt (Figs 2G and EV1B). This suggests that the important regulatory elements located at the 3′ end of *DOG1* are not required for the induction of *DOG1* by salt stress.

### *PUPPIES* are positive regulators of *DOG1* expression in response to salt stress

We observe that both *PUPPIES* and *DOG1* are induced by salt. To understand the causative relationship between them, we first tested whether *PUPPIES* control *DOG1* expression. We used a T-DNA mutant (*puppies-1*) with the insertion around 400-bp upstream of *DOG1* TSS (Fig 3A). RT–qPCR shows that all *PUPPIES* isoforms are significantly downregulated in *puppies-1* (Fig 3B). Notably, in *puppies-1*, we also observe a strong downregulation of *DOG1* expression (Fig 3B). Consistent with low *DOG1* expression, *puppies-1* mutants display a weaker inhibition of seed germination upon salt stress (Fig 3C). These results suggest a positive impact of *PUPPIES* on *DOG1* expression and delay of germination.

Importantly, RT–qPCR on *dog1-3* shows that *DOG1* knockout does not result in downregulation of *PUPPIES*, except for the isoform in which transcription is directly blocked by the T-DNA insertion in this mutant (Fig EV1C). We speculate that the loss of *PUPPIES-fusion* caused by the T-DNA insertion on *dog1-3* leads to a compensating increase in the levels of unspliced *PUPPIES* (*PUPPIES-uns*; Fig EV1C). Additionally, we show that *PUPPIES* expression is still induced by salt in the *dog1-3* background (Fig EV1D). The same could be observed in the 3'RNA-seq data for the *dog1-3* mutant (Fig 2A). In short, *PUPPIES* knock-down is associated with a strong *DOG1* downregulation, yet *DOG1* knockout does not downregulate *PUPPIES* nor affect *PUPPIES* responsiveness to salt. Moreover, *DOG1* gene expression is known to be induced in seeds during imbibition under prolonged heat stress causing a re-induction of seed dormancy, called secondary seed dormancy (Argyris *et al*, 2008, Leymarie *et al*, 2008). In agreement with published data (Krzyszton *et al*, 2022), *DOG1* expression is strongly induced during secondary dormancy induction. By contrast, *PUPPIES* expression is not induced but rather shut down (Fig EV1E). This supports the idea that *PUPPIES* regulation differs from that of *DOG1* and that *PUPPIES* are induced by only some of the *DOG1*-inducing signals.

In contrast to secondary dormancy, during seed maturation, *PUPPIES* expression has the same dynamics as *DOG1* (Appendix Fig S5A). In agreement, *puppies-1* displays weaker primary seed dormancy (Appendix Fig S5B), a result consistent with the idea that *PUPPIES* induce *DOG1* expression not only in response to salt but also during primary seed dormancy establishment. We propose that *PUPPIES* are partially independent of *DOG1* and that upon salt stress *PUPPIES* act upstream of, and as a positive regulator of *DOG1* expression to control seed germination.

Next, we used CRISPR-Cas9 aiming to create deletions in the *PUPPIES* region. We obtained a mutant with slower seed germination under salt stress (Fig 3D). Sanger sequencing reveals a deletion (from −186 to −449) upstream of *PUPPIES* TSS (Fig 3A, and Appendix Fig S5C and D). This leads us to hypothesize that the deletion may remove a negative regulatory element on the *PUPPIES* promoter resulting in *PUPPIES* overexpression, which would explain the phenotype. RT–qPCR confirms *PUPPIES* upregulation in the deletion mutant (*puppies-ox*; Fig 3E) and induction of *DOG1*


© 2023 The Authors

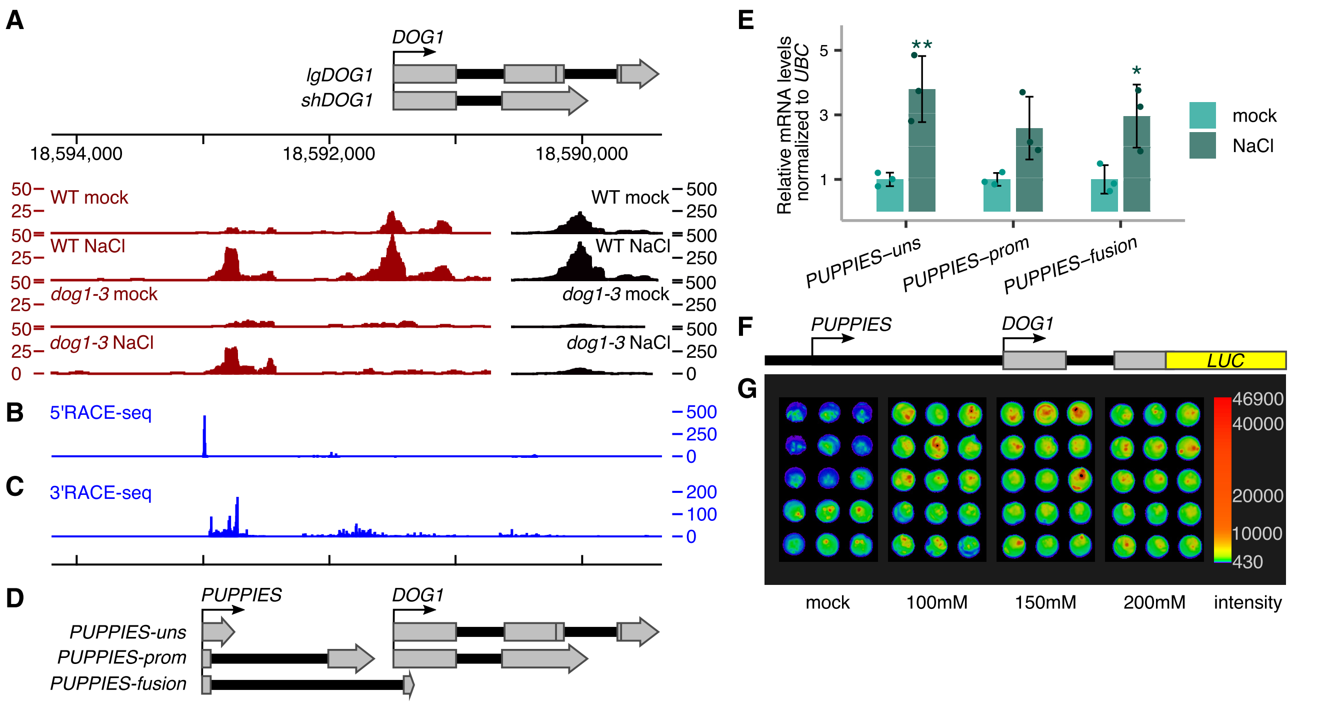


**Figure 2. Transcriptional activity of salt-sensing element on *DOG1* promoter.**

A 3'RNA-seq read coverage on *DOG1 locus*. Black colour is used for coverage within the scale on the right-hand side (0–500), and red is used for coverage within the scale on the left-hand side (0–50). Above is a schematic representation of the annotated transcripts from the *DOG1 locus* and the chromosome coordinates.
B 5'RACE-seq from a primer within the *DOG1* promoter reveals a transcription start site (TSS) upstream of the *DOG1* gene.
C 3'RACE-seq results show novel transcription termination sites (TTS) along the *DOG1* promoter and gene body.
D Schematics of newly annotated *PUPPIES* isoforms, co-directionally transcribed upstream of *DOG1*.
E RT–qPCR with primers specific for *PUPPIES-uns*, *PUPPIES-prom*, and *PUPPIES-fusion* isoforms. Expression levels normalized to *UBC21* for 100 mM NaCl-treated seeds and relative to mock. Bars and error bars represent the mean ± SD. Points represent biological replicates. $^*P$-value < 0.05, $^{**}P$-value < 0.01 from two-tailed Student's *t*-test.
F Schematics of *psDOG1::LUC* construct with Luciferase reporter sequence inserted at the end of *DOG1* exon 2 (Fedak *et al*, 2016).
G Luciferase activity in seeds in different concentrations of NaCl or mock. The colour bar shows the luminescence in counts per second on a logarithmic scale.

expression in seeds under salt stress (Fig 3E). Additionally, we show that *puppies-ox* seeds display stronger primary dormancy (Appendix Fig S5E) and higher expression of *PUPPIES* and *DOG1* compared with WT at late stages of seed maturation (Appendix Fig S5F).

Additionally, we used a dCas9 system to interfere with *PUPPIES* transcription without changing the genomic sequence, which could alter *DOG1* expression. This was achieved using transgenes carrying one of three different pairs of sgRNAs together with a constitutively expressed catalytically inactive or "dead" Cas9 (dCas9) protein which is thought to bind but not to cleave DNA and therefore has been used as a roadblock for transcription (Bikard *et al*, 2013; Qi *et al*, 2013; Piatek *et al*, 2015). We show that guiding dCas9 to three different regions of *PUPPIES* (Fig EV2A) causes faster germination of seeds under salt stress (Fig EV2B). We selected four independent transgenic lines from one construct for gene expression analysis. As expected, *PUPPIES* expression is significantly downregulated in the dCas9 lines, importantly consistent with faster germination (Fig EV2B) we observe downregulation of *DOG1* expression (Fig EV2C). These results support the idea that *PUPPIES* affect the expression of *DOG1* independently of changes in its genomic sequence.

Based on a series of *PUPPIES* mutants and the correlation of expression patterns during seed maturation and salt stress, we suggest that *PUPPIES* lncRNAs act as positive regulators of *DOG1* expression. Additionally, *PUPPIES* seem to be important for *DOG1* regulation in seeds under salt stress in contrast to heat stress.

### *PUPPIES* induce *DOG1* in *cis*

To understand whether *PUPPIES* work in *cis* or in *trans*, we expressed *PUPPIES* from a different allele to that expressing *DOG1*. For that, we crossed the *PUPPIES* knock-down mutant *puppies-1* with the *DOG1* knockout *dog1-3*. We then asked whether *PUPPIES*, expressed from the *dog1-3* allele, are able to induce *DOG1* expression from the *puppies-1* allele in the heterozygous F1 generation (Fig EV3A). In seeds imbibed in the presence of NaCl, RT–qPCR shows higher levels of *PUPPIES* in the heterozygous F1 relative to homozygous *puppies-1* F1 (Fig EV3B), consistent with *PUPPIES* transcription from the *dog1-3* allele. By contrast, we do not detect higher levels of *DOG1* expression in the heterozygous F1 seeds (Fig EV3B). These results suggest that *PUPPIES* are unable to activate *DOG1* expression in *trans*. We further tested this hypothesis by crossing either WT or *puppies-ox* with two independent *psDOG1::LUC*

© 2023 The Authors 

reporter lines (Fig EV3C). In the heterozygous F1 generation, we measured *DOG1* expression from the *psDOG1::LUC* allele using primers specific to *DOG1-LUC* transcripts to test whether the higher expression of *PUPPIES* from the *puppies-ox* allele leads to the activation of the *DOG1* reporter. RT–qPCR in F1 seeds imbibed in the presence of NaCl show no changes in *DOG1*-linked reporter in any of the crosses (Fig EV3D), suggesting that changes in *PUPPIES* expression originating from *puppies-ox* allele does not affect *DOG1* expression from the transgene. Based on these results, we hypothesize that *PUPPIES* lncRNAs induce *DOG1* expression in *cis* but not in *trans*.

### *PUPPIES* regulate *DOG1* transcription

As shown previously, maturing seeds of the *puppies-ox* mutant have higher expression of *DOG1* and *PUPPIES* when compared to WT. Therefore, the observed high levels of *DOG1* and *PUPPIES* in *puppies-ox* seeds imbibed in salt could be a consequence of persistently high levels of mRNA accumulated during seed maturation in this mutant. Contrary to this idea, when compared to WT, the *puppies-ox* mutant shows a higher fold induction of *PUPPIES* and *DOG1* in seeds under salt stress than the fold induction observed in

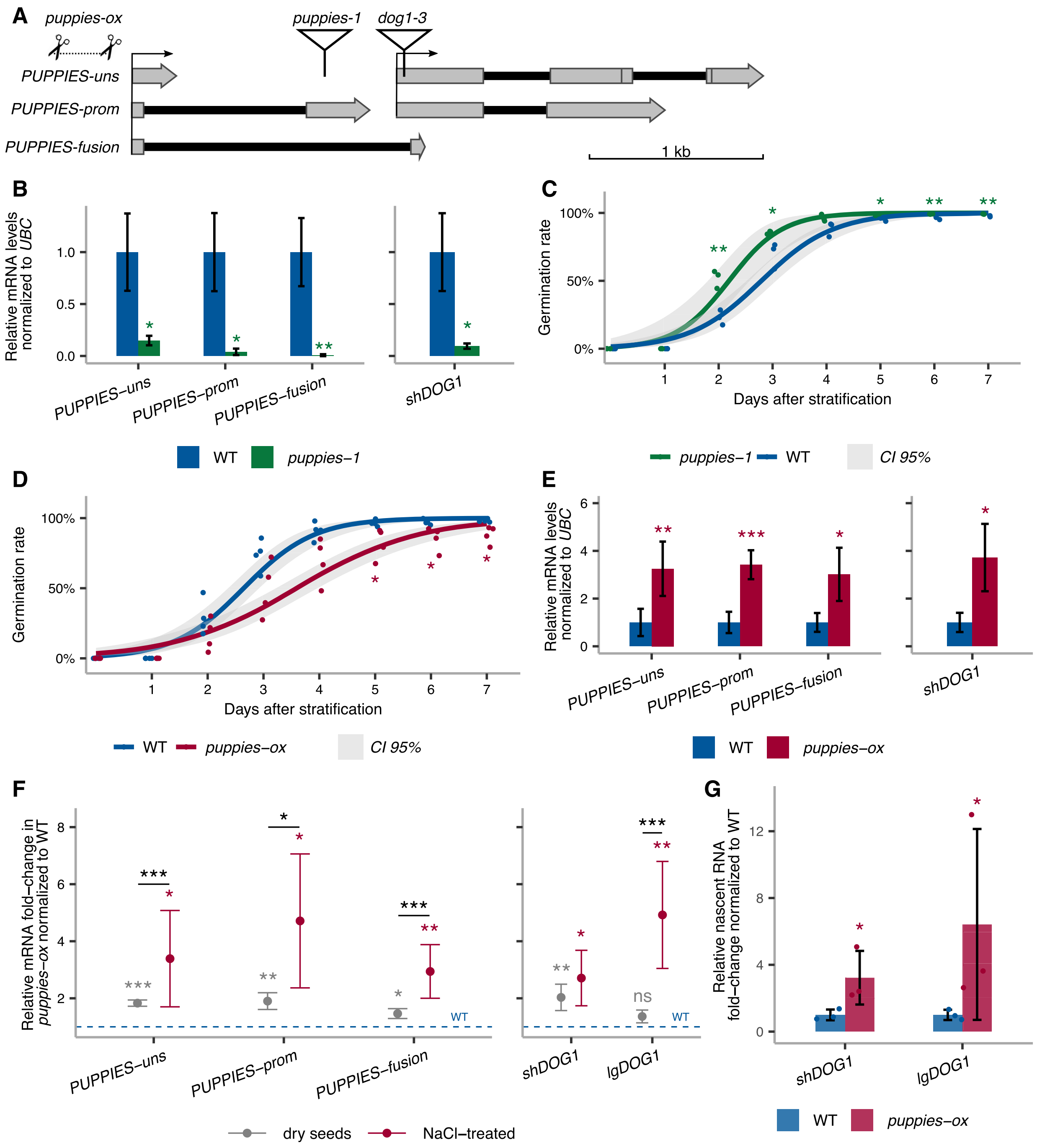


**Figure 3.**


© 2023 The Authors

**Figure 3. *PUPPIES*-mediated changes in *DOG1* gene expression and salt stress response.**

A Schematics of *DOG1 locus* with *PUPPIES* and *DOG1* TSS indicated by black arrows. The T-DNA insertions in *puppies-1* and *dog1-3* mutants, and CRISPR-Cas9 deletion in *puppies-ox* are shown.

B RT–qPCR with primers specific for *PUPPIES-uns*, *PUPPIES-prom*, *PUPPIES-fusion* and *shDOG1*. Expression levels normalized to *UBC21* in seeds treated with 100 mM NaCl in the *puppies-1* mutant relative to WT.

C, D Germination time-course in 100 mM NaCl after stratification for *puppies-1* (C) and *puppies-ox* (D) relative to WT. Lines represent fitted curves with a 95% confidence interval (grey area), dots represent data points, **P*-value < 0.05 from the two-tailed Student's *t*-test. (C) Data for WT are the same as plotted in Fig 1B.

E RT–qPCR (same as in B) in *puppies-ox* relative to WT.

F RT–qPCR fold-change induction of *PUPPIES-uns*, *PUPPIES-prom*, *PUPPIES-fusion*, *shDOG1* and *lgDOG1* in *puppies-ox* relative to WT (blue dashed line), in dry (light grey) and imbibed seeds in the presence of 100 mM NaCl (red). Significance from the two-tailed Student's *t*-test for comparing dry seeds of WT and *puppies-ox* is represented with light grey asterisks, and salt-imbibed seeds of WT and *puppies-ox* are represented with red asterisks. Black asterisks represent significance from two-way ANOVA with Tukey's multiple comparisons test for dry seeds versus imbibed seeds. ns *P*-value > 0.05, **P*-value < 0.05, ***P*-value < 0.01, ****P*-value < 0.001. Error bars represent the mean ± SD. *n* = 4 biological replicates.

G RT–qPCR with primers for *shDOG1* and *lgDOG1* on nascent RNA from seeds imbibed in 100 mM NaCl from *puppies-ox* relative to WT. Nascent RNA levels were normalized to *UBC21*. Bars and error bars represent the mean ± SD. Points represent biological replicates. Statistical significance from two-tailed Student's *t*-test. **P*-value < 0.05.

Data information: (B, E) Bars and error bars represent the mean ± SD. Statistical significance from two-tailed Student's *t*-test. **P*-value < 0.05, ***P*-value < 0.01, ****P*-value < 0.001.

dry seeds (Fig 3F). This suggests that in response to salt, the high levels of *PUPPIES* and *DOG1* in *puppies-ox* are a result of *de novo* transcription after imbibition, and not pre-existing high mRNA levels in dry seeds.

Upstream pervasive transcription has been demonstrated to silence downstream genes (Nguyen *et al*, 2014). Surprisingly, *PUPPIES* positively regulate the levels of *DOG1* steady-state mRNA. To test *PUPPIES* effect on *DOG1* expression at the transcriptional level, we employed a procedure to isolate nascent RNAs attached to the chromatin (chrRNA). RT–qPCR on chrRNA shows higher levels of *DOG1* nascent RNA in *puppies-ox* (Fig 3G). This suggests that *PUPPIES* act by activating *DOG1* at the transcriptional level.

## smFISH reveals changes in *DOG1* transcription dynamics

Single-molecule RNA fluorescence *in situ* hybridization (smFISH) is a technique used to image single RNA transcripts, thus allowing the examination of transcriptional features of gene expression regulation (Femino *et al*, 1998; Raj *et al*, 2006, 2008). In plants, this method was previously used for the roots of young seedlings to describe multiple aspects of transcriptional regulation of the *FLC* gene (Rosa *et al*, 2016; Duncan & Rosa, 2017; Ietswaart *et al*, 2017). Here, we adapted this method to image *DOG1* transcripts in *Arabidopsis* embryos. *DOG1* smFISH reveals multiple *foci* distributed in the cytoplasm and nucleus of the embryo cells from seeds under salt stress (Figs 4A and EV4A–C), which were absent in *dog1-3* (Fig EV4D and E), and sensitive to RNase A treatment (Fig EV4F). A fraction of cells shows one or two brighter *foci* that can be interpreted as genomic *loci* with active transcription (transcription sites; Figs 4A, and EV4A and C) containing multiple nascent transcripts as reported before (Mueller *et al*, 2013; Gómez-Schiavon *et al*, 2017). We detect on average 20 *foci* (*DOG1* transcripts) per cell (Fig 4B). Importantly, smFISH shows a drastic reduction of cytoplasmic *foci* (*DOG1* mRNA) in *puppies-1* compared with WT (Figs 4C and D, and EV4G), consistent with RT–qPCR (Fig EV4H). The distribution of intensities of cytoplasmic *foci* in *puppies-1* is not significantly changed compared with WT (Fig EV4I). Notably, we observe a significant reduction in intensities for the brightest nuclear *foci* that we assume to correspond to *DOG1* transcription sites (TS; Fig 4C and E). This suggests that *puppies-1* has a lower number of nascent or chromatin-attached *DOG1* transcripts per round of transcription. Additionally, we detect a lower fraction of cells with TS *foci* in *puppies-1* (Fig 4F).

By contrast, *puppies-ox* shows no significant difference in the frequency of the cells with active *DOG1* transcription, compared with WT (Fig 4G and H). One possibility is that the frequency of active transcription is intrinsic to the T-DNA insertion. On the contrary, the observed changes may be a consequence of lower intensity of TS containing only a few transcribing polymerases, which will become indistinguishable from the intensity of one full-length transcript, and will therefore not be counted in our analysis as a *DOG1* TS but as an mRNA.

Yet, in agreement with changes in *puppies-1*, we do observe a higher intensity of TS in *puppies-ox* (Fig 4G and I), suggesting a higher number of nascent or chromatin-attached *DOG1*, consistent with RT–qPCR from chrRNA (Fig 3G). The release of Pol II from promoters often happens in bursts with the transcription by several Pol II enzymes followed by a period of absence of initiation events, a phenomenon called transcriptional bursting. The number of Pol II per burst of transcription is defined as transcriptional burst size (Raj *et al*, 2006; Cai *et al*, 2008; Zenklusen *et al*, 2008; Suter *et al*, 2011). Our results could suggest that *PUPPIES* affect *DOG1* burst size. However, the smFISH intensity signal of TS is also affected by the length of the transcripts, as longer nascent transcripts allow the binding of a higher number of fluorescently labelled probes. Therefore, we isolated chrRNA and calculated the ratio of 3′ to 5′ ends for *DOG1*, so that a higher 3′ to 5′ ends ratio would reflect increased abundance of longer transcripts.

Our analysis shows no significant changes in the ratio between 3′ and 5′ ends of chrRNA in the *PUPPIES* mutants compared with WT, which suggests that *PUPPIES* do not change the length of *DOG1* nascent transcripts attached to chromatin, but rather the number of Pol II molecules per round of *DOG1* transcription (burst size). Consistent with that, we also observe an increase in the number of cytoplasmic *DOG1* mRNA in the *puppies-ox* mutant (Fig 4J).

The smFISH experiments reveal changes in mRNA number and transcriptional bursting in the *PUPPIES* mutant's seeds under salt stress. In agreement, smFISH analysis on WT embryos from mock and NaCl-treated seeds shows that salt stress induces the number of *DOG1* mRNA per cell (Fig EV5A–C). A result that is consistent with

our previous data (Figs 1D and F, 2A and G, and Appendix Fig S1C). Additionally, we observe a higher frequency of cells with active *DOG1* transcription (Fig EV5D) and higher intensity of *DOG1* TS (Fig EV5E).

Our results suggest that *PUPPIES* modulate features of *DOG1* transcriptional bursting such as burst size and are consistent with a recent work showing the involvement of *cis*-acting lncRNAs in controlling transcriptional burst size or frequency of the nearby genes (Johnsson *et al*, 2022).

### *PUPPIES* regulate *DOG1* transcription pausing and processivity

Given the observed changes in Pol II burst size, we speculate that *PUPPIES* could affect *DOG1* Pol II processivity. We, therefore, used a modified version of targeted nascent RNA analysis (Oesterreich *et al*, 2016; Herz *et al*, 2019) referred to as targeted NET-seq (Native Elongating Transcript sequencing) hereafter. Briefly, chrRNA was purified from seeds imbibed under salt stress, adapters ligated at 3′ ends, followed by library preparation using primers spanning the entire *DOG1* gene, and next-generation sequencing. After UMI-based PCR duplicates removal and filtering reads mapped to *DOG1*, we ended up with around 19,000 unique reads per each biological replicate of WT and *puppies-ox*. For each primer, we plotted 3′ ends of reads, giving us a single nucleotide resolution map of Pol II position along the *DOG1* transcriptional unit.

Strikingly, we observe a drastic increase of Pol II density in *puppies-ox* mutant around 300-bp downstream of *DOG1* TSS, where a pause site is revealed by a sharp peak in WT (Fig 5A). These results suggest that *PUPPIES* overexpression causes Pol II to strongly accumulate at this *DOG1* promoter-proximal pause site. Since the total unspliced *PUPPIES* attached to the chromatin (including those that may contain *DOG1* exon 1) constitutes only 2.9% of the total chrRNA containing *DOG1* exon 1 (Appendix Fig S6A), the strong NET-seq peak is unlikely to reflect *PUPPIES*-transcribing Pol II density over this region. Moreover, targeted NET-seq shows that in *puppies-ox*, Pol II travels slower through exons (Fig 5A–C) but not through introns (Fig 5D and E). The enhanced promoter-proximal pausing together with slower transcription over exons but not introns suggest an augmented difficulty in Pol II transcribing through nucleosomes. We overlapped our targeted NET-seq signal with the mapping of nucleosome occupancy on the *DOG1 locus* from public MNase-seq data (Data ref: Luo *et al*, 2020b; Appendix Fig S6B) and nucleosome occupancy prediction based on DNA sequence (van der Heijden *et al*, 2012; Appendix Fig S6C). Notably, we detect a nucleosome at or immediately downstream of nearly all regions with enhanced pausing in the *puppies-ox* mutant, including the promoter-proximal pausing site (Appendix Fig S6D and E). This observation supports the hypothesis that *PUPPIES* enhance *DOG1* expression by modulating *DOG1* transcriptional dynamics through nucleosomes.

In summary, our extensive transcriptional analyses reveal that *PUPPIES* induce *DOG1* transcription. Surprisingly, the positive impact of *PUPPIES* on *DOG1* transcription is associated with an augmented promoter-proximal pausing and slower transcription through exonic regions, possibly by quelling Pol II ability to pass through nucleosomes. We hypothesize that at the same time these changes allow the loading of a higher number of Pol II molecules per round of transcription as revealed by smFISH.

### *PUPPIES* modulation of *DOG1* transcription feeds back on the splicing outcome

Slower transcription processivity was shown to enhance splicing efficiency and remodel alternative splicing events (Neugebauer, 2002; Naftelberg *et al*, 2015; Saldi *et al*, 2016). In plants, transcription elongation rate was shown before to contribute to alternative splicing and gene expression regulation, including *DOG1* (Dolata *et al*, 2015; Herz *et al*, 2019). Since in *puppies-ox* we observe changes consistent with slower transcription of *DOG1* exons, we postulate that *PUPPIES* also affect *DOG1* splicing. Indeed, *puppies-ox* displays enhanced *DOG1* splicing efficiency revealed by lower *DOG1* intron 1 retention levels and changes in *DOG1* intron 2 alternative splicing (Fig 5F). In agreement, we observe lower splicing efficiency and opposite *DOG1* alternative splicing changes in *puppies-1* (Fig 5G). Furthermore, we tested whether the splicing changes observed in steady-state mRNA were also detected in nascent RNAs. RT–qPCR on chrRNA showed higher and lower splicing efficiency in *puppies-ox* and *puppies-1*, respectively (Fig 5H), consistent with the previous results (Fig 5F and G).

If the differences in transcription dynamics observed by targeted NET-seq in *puppies-ox* were a consequence of splicing, we would expect those to be observed during/after the transcription of introns. Instead, we observe the most striking difference in Pol II pausing at the beginning of the gene. This suggests that splicing of *DOG1* is rather a consequence, and not a cause of Pol II processivity.

## Discussion

Based on our results, we propose a model in which the *DOG1* promoter region generates multiple lncRNAs, named *PUPPIES*. *PUPPIES* are induced by salt stress and their pervasive transcription induces *DOG1* expression (Fig 6). Surprisingly, on the *DOG1 locus*, *PUPPIES* invasive transcription does not result in a negative interference with gene expression but in the induction of *DOG1* expression. This causes a delay in seed germination under salt stress. The positive effect of *PUPPIES* on *DOG1* is associated with changes in Pol II transcriptional dynamics. Particularly, *PUPPIES* induce stronger Pol II promoter-proximal pausing on the *DOG1* gene, slower transcription through exons, and loading of a higher number of Pol II complexes per *DOG1* transcriptional burst. Ultimately, *PUPPIES*-mediated changes in *DOG1* transcription enhance splicing efficiency and affect alternative splice-site selection.

*DOG1* gene is a well-known regulator of primary dormancy strength that regulates germination timing in fresh seeds (Alonso-Blanco *et al*, 2003; Bentsink *et al*, 2006; Nakabayashi *et al*, 2012). Here, we identify a function of the *DOG1* gene in controlling the speed of germination under salt stress (Fig 1). We show that ionic imbalance induces *DOG1* expression (Fig 1D–F and Appendix Fig S2A), which results in a delay in germination. By contrast, osmotic stress caused by PEG results in the downregulation of *DOG1* (Appendix Fig S2B). We note that PEG is used to prime seeds for germination (Heydecker *et al*, 1973; Waqas *et al*, 2019); therefore, it seems possible that *DOG1* downregulation by PEG may contribute to this process.

Very little is known about the response of seeds to salt. Our 3’RNA-seq shows that in response to salt stress, seeds overexpress

 © 2023 The Authors

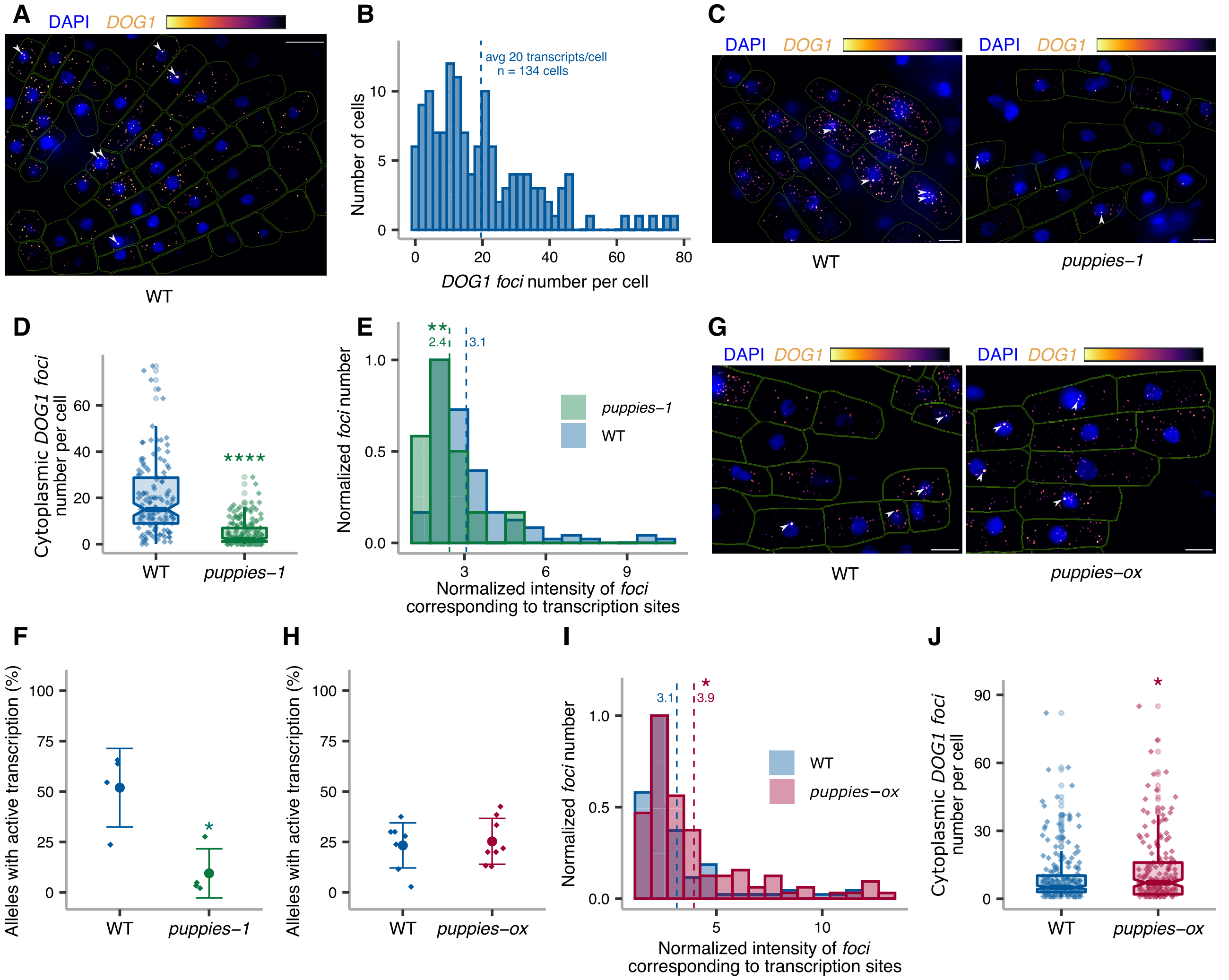


**Figure 4. *DOG1* transcriptional regulation by *PUPPIES* revealed by single-molecule RNA FISH.**

- A *z*-stack max-projection image of smFISH for *DOG1* RNA. The "Inferno" colour scale is used for the intensity of fluorescence from Quasar670 fluorophore (*DOG1*). The blue colour shows fluorescence from DAPI (nuclei staining). Arrowheads point to *foci* corresponding to transcription sites (TS). The scale bar is 20 μm.
- B Distribution of cytoplasmic *DOG1 foci* per cell in WT. The blue vertical dashed line indicates the average.
- C *z*-stack max-projection images of *DOG1* smFISH in seeds imbibed in 100 mM NaCl of WT (left) versus *puppies-1* (right). Arrowheads point to *foci* corresponding to TS. The scale bar is 5 μm.
- D Notched box plots showing the cytoplasmic *DOG1 foci* number per cell in WT and *puppies-1*. Diamond-shaped points represent each cell, $n = 134$ cells from WT and $n = 182$ cells from *puppies-1*.
- E Distribution of intensities of *foci* corresponding to *DOG1* transcription sites in WT and *puppies-1*. In the *x*-axis is the fluorescence intensity fold-change of *foci* classified as TS to the average intensity of nuclear *foci*. In the *y*-axis is the number of *foci* normalized to their maximum value. Vertical dashed lines indicate the average fluorescence fold-change for WT (3.1) and *puppies-1* (2.4). $n = 135$ *foci* from WT, $n = 29$ *foci* from *puppies-1*.
- F Plot showing the frequency of alleles with detected *foci* corresponding to *DOG1* TS for WT and *puppies-1*. Points and error bars represent the mean ± SD. Diamond-shaped points represent single embryos.
- G *z*-stack max-projection images from seeds imbibed in 100 mM NaCl of WT (left) versus *puppies-ox* (right). Arrowheads point to *foci* corresponding to TS. The scale bar is 5 μm.
- H Same as (F), for WT and *puppies-ox*.
- I Same as in (E), in WT and *puppies-ox*. Vertical dashed lines indicate the average fluorescence fold-change for WT (3.1) and *puppies-ox* (3.9). $n = 107$ *foci* from WT and $n = 105$ foci from *puppies-ox*.
- J Notched box plot, same as (D), in WT and *puppies-ox*. $n = 212$ cells from WT, $n = 179$ cells from *puppies-ox*.

Data information: (D–F, H–J) Statistical significance from two-tailed Student's *t*-test. **P*-value < 0.05, ***P*-value < 0.01, *****P*-value < 0.0001. (D and J) The box plot's central band marks the median, lower and upper box limits mark the first and third quartiles, whiskers extend the boxes to the largest value no further than 1.5 times the interquartile range, and the notches extend to 1.58 times the interquartile range divided by sqrt (*n*).

© 2023 The Authors 

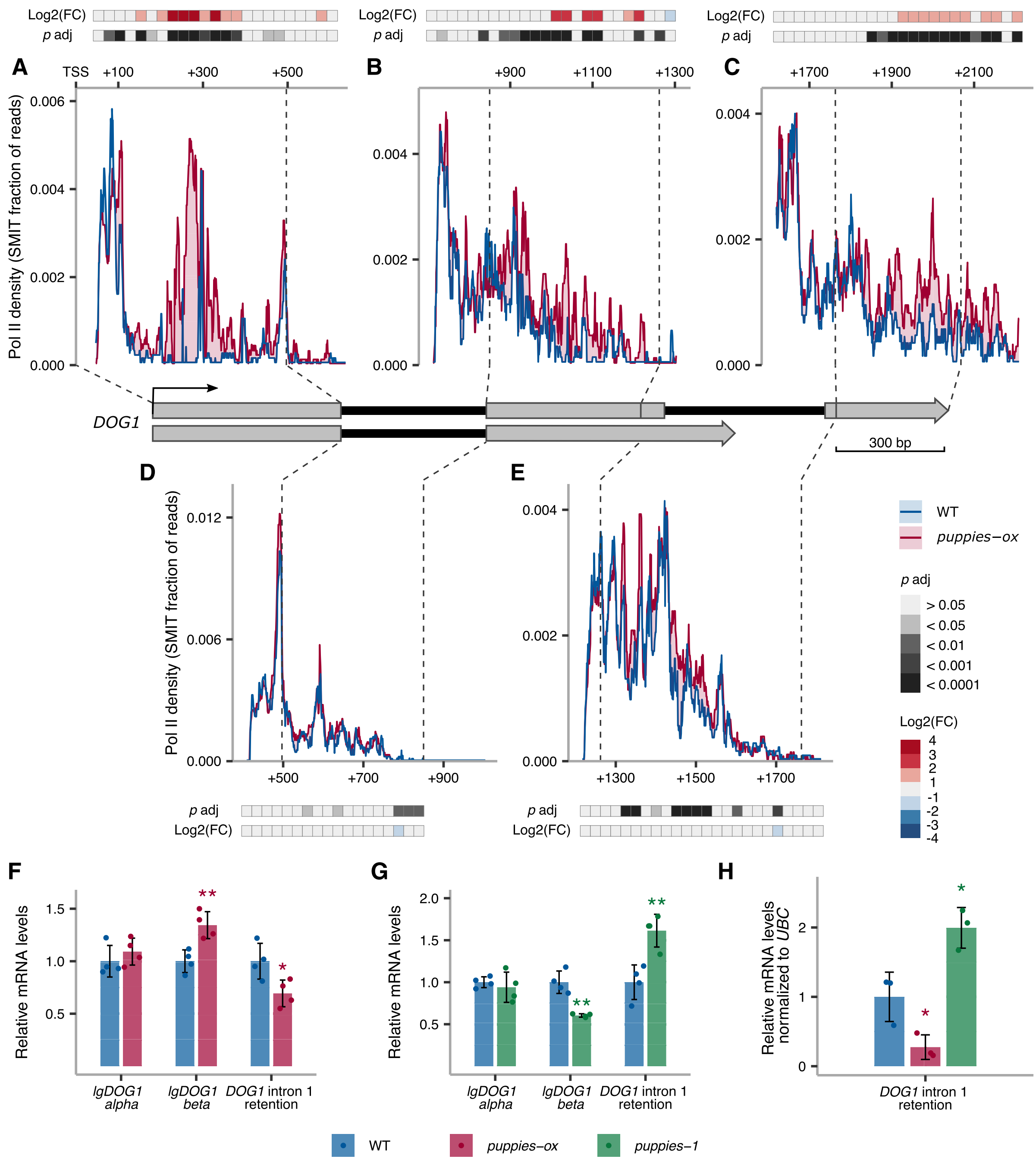


**Figure 5. *DOG1* transcription pausing and processivity dependent on *PUPPIES*.**

A–E The profile of Pol II during *DOG1* transcription. Plots show the fraction of unique reads from targeted NET-Seq using rolling median (11 nt). Targeted libraries for sequencing of *DOG1 locus* were obtained by usage of five primers across the gene. Pol II dynamics are analysed separately for each region sequenced in exon 1 (A), exon 2 (B), exon 3 (C), intron 1 (D) and intron 2 (E). The *x*-axis shows the distance in bp from *DOG1.* A light red colour fill highlights the regions where Pol II density is higher in *puppies-ox*. A light blue colour fill highlights the regions where Pol II density is higher in WT. For each plot, coloured tiles represent the fold-change (*puppies-ox*/WT) on a logarithmic scale. Greyscale tiles represent the result of a statistical test for the difference between genotypes from the two-tailed Student's *t*-test after Bonferroni correction. Tiles correspond to a 25 bp bin each. Vertical dashed lines connect the exon boundaries on the plots with the schematics of the *DOG1* gene with grey boxes representing exons and black lines representing introns.

F, G RT–qPCR measurement of alternative splicing of *lgDOG1* alpha and beta isoforms and unspliced intron 1 *DOG1* isoforms levels in *puppies-ox*, and *puppies-1* relative to WT, in seeds treated with 100 mM NaCl.

H RT–qPCR quantification of *DOG1* intron 1 retention on nascent RNA from seeds imbibed in 100 mM NaCl from *puppies-ox* and *puppies-1* relative to WT. Nascent RNA levels were normalized to *UBC21.*

Data information: (F–H) Bar and error bars represent the mean ± SD, and points represent biological replicates. *$P$-value < 0.05, **$P$-value < 0.01 from two-tailed Student's *t*-test.

 © 2023 The Authors

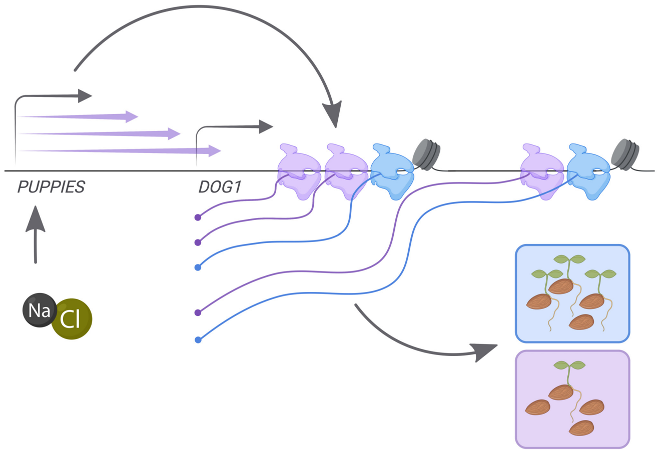


**Figure 6. Model for molecular regulation of *DOG1* gene expression mediated by *PUPPIES* in seeds under salt stress.**

Schematic representation of a model in which *PUPPIES* control *DOG1* expression upon salt stress during seed imbibition. The blue colour is used to represent a WT state with low levels of *DOG1* transcription after imbibition and fast seed germination. The purple colour is used to represent a state in which *PUPPIES* pervasive transcription on *DOG1* (purple arrows) is induced upon salt stress. The induction of *PUPPIES* causes the *DOG1* gene to be transcribed by additional (purple) Pol II molecules (higher transcriptional burst size). This state also includes enhanced promoter-proximal pausing, and slower transcription over exonic regions represented by a nucleosome. This results in a higher *DOG1* mRNA production and consequently a delay in seed germination. The TSS of *PUPPIES* and *DOG1* is indicated with grey arrows above the gene name. Additional grey arrows represent effects. This figure was created with BioRender.com.

genes involved in salt tolerance in vegetative tissues (Fig 1J), including positive regulators of salt tolerance, such as *NA⁺/H⁺ exchanger 1* (NHX1, Apse *et al*, 1999; Zhang & Blumwald, 2001; Zhang *et al*, 2001) and Salt-induced serine rich (SIS, Brinker *et al*, 2010). Additionally, salt stress promotes the expression of several negative regulators of seed germination (Fig 1J), including *DOG1*. This suggests that the salt-induced delay of germination is not uniquely dependent on *DOG1* but possibly on a combination of several players. Interestingly among them, *reduced dormancy 5* (*RDO5*; Xiang *et al*, 2014) and *Abscisic acid insensitive 3* (*ABI3*, Giraudat *et al*, 1992) were previously identified as candidate genes underlying *QTLs* for natural variation to salt tolerance (Quesada *et al*, 2002). Others include *ABA deficient 1* (*ABA1/ZEP*) shown to promote tolerance to osmotic stress (Koornneef *et al*, 1982; Barrero *et al*, 2005; Park *et al*, 2008), *late embryogenesis abundant 6* (*LEA6/ATEM6*) known to contribute to desiccation tolerance in seeds (Manfre *et al*, 2009) and *1-cysteine peroxiredoxin 1* (*PER1*) a seed-specific peroxiredoxin responsible for eliminating reactive oxygen species (ROS) and repression of germination by suppressing ABA catabolism and GA biosynthesis (Haslekås *et al*, 1998; Chen *et al*, 2020).

The ability of seeds to germinate rapidly and uniformly under suboptimal conditions is a valuable agronomical trait, especially in the light of current threats such as climate change and the loss of fertile soil. Our results reveal that under suboptimal conditions imposed by NaCl, seeds overexpress a selection of key regulators of seed germination, including *DOG1*. Misregulation of *DOG1* expression compromises the transcriptomic response to salt and alters the speed of germination upon salt stress. This supports a previously uncharacterized role of the *DOG1* gene in the salt-induced delay of germination.

Based on a series of 3’RNA-seq, 5′ and 3’RACE-seq and Sanger sequencing, we characterize *PUPPIES* lncRNAs, co-directionally transcribed from a TSS located ~1.5-Kb upstream of the *DOG1* gene (Fig 2A and B). *PUPPIES* are a result of pervasive transcription over the *DOG1* promoter and gene body and are extensively spliced (Fig 2A and D, and Appendix Fig S3). We show that *PUPPIES* are induced both during seed maturation and during salt stress in seeds concomitant with induction of *DOG1* expression (Fig EV1A and Appendix Fig S5A). Downregulation of *PUPPIES* expression caused by T-DNA insertion or upregulation of *PUPPIES* expression by CRISPR-Cas9 deletion in *PUPPIES* promoter leads to down and upregulation of *DOG1* expression, respectively (Fig 3A–E). These results are consistent with the positive role of *PUPPIES* on *DOG1*. The two regions of *PUPPIES* altered in the mutants are fairly distant from each other. Yet, as *PUPPIES* genomic sequence coincides with the *DOG1* promoter region, it is possible that the sequence alterations in the mutants would impact *DOG1* directly. Importantly, the dCas9 transcription-blocking system (Bikard *et al*, 2013; Qi *et al*, 2013; Piatek *et al*, 2015), which downregulates *PUPPIES* expression without altering its underlying genomic sequence also reduces *DOG1* expression (Fig EV2). It is, however, possible that the binding of dCas9 to its target sequence on the *DOG1* promoter occludes the binding of transcription factors. To reduce the influence of such an undesired secondary effect, we targeted dCas9 to three different regions along the *DOG1* promoter. Transgenic plants carrying the different constructs consistently display faster seed germination under salt stress. These results suggest that changes in *PUPPIES* expression affect *DOG1* gene expression independently of changes in the DNA sequence.

*PUPPIES* expression is positively correlated with *DOG1* expression in response to salt and during seed maturation. Yet, *DOG1* knockout does not downregulate *PUPPIES* (Fig EV1C) nor affects *PUPPIES* responsiveness to salt stress (Fig EV1D). Moreover, during secondary dormancy induction with heat stress, the strong *DOG1* induction is not followed by induction of *PUPPIES*, instead *PUPPIES* expression seems to be shut down (Fig EV1E). These results suggest that while some cross regulation between *PUPPIES* and *DOG1* possibly exists, *PUPPIES* regulation is partially independent of *DOG1*. Analyses of different heterozygous plant combinations allowed us to test the influence of *PUPPIES* from one allele on *DOG1* expression from a different allele as well as from a transgene (Fig EV3A and C). Our results suggest that *PUPPIES* are only able to induce *DOG1* expression in *cis* (Fig EV3B and D).

Consistent with a *cis*-mode of action, we speculated that *PUPPIES* would locally impact *DOG1* transcription. To study *DOG1* transcriptional dynamics, we performed smFISH in embryos of seeds under salt stress from the *PUPPIES* mutants. We observe changes in the fluorescence intensity of *foci* corresponding to transcription sites, consistent with *PUPPIES* inducing a higher number of Pol II that transcribe *DOG1* during each burst (Figs 4 and EV4). Cotranscriptional RNA processing is an important feature of gene expression in plants (Marquardt *et al*, 2022). Therefore, the higher intensity of TS could also reflect changes in chromatin-attached *DOG1* RNA length. A result of differential distribution of Pol II or mRNA retention at the site of transcription. To test this possibility, we measured the 3′

© 2023 The Authors 

to 5′ ratio of *DOG1* chrRNA by RT–qPCR. No changes in 3′/5′ ratio are observed in *PUPPIES* mutants compared with WT (Fig EV4K) Furthermore, the levels of *DOG1* intron 1 in chrRNA are lower in *puppies-ox* and higher in *puppies-1* (Fig 5H), opposite to the smFISH intensity of TS.

Therefore, the smFISH signal of TS is unlikely to be explained by changes in cotranscriptional splicing efficiency, mRNA release or Pol II distribution on *DOG1*, and is more likely to reflect differences in the number of transcribing Pol II (burst size). Moreover, salt stress also induces *DOG1* transcriptional burst size and the frequency of active transcription (Fig EV5).

In contrast to *PUPPIES*, most lncRNAs acting on neighbouring genes have been shown to repress their transcription by locally inducing a repressive chromatin environment (Swiezewski *et al*, 2009; Heo & Sung, 2011; Kim *et al*, 2012; Ariel *et al*, 2014; Kim & Sung, 2017). Alternatively, pervasive transcription can cause what is called transcriptional interference (Shearwin *et al*, 2005; Villa *et al*, 2022). In plants, transcription interference was shown to occur upon overlapping convergent transcription possibly through Pol II collisions (Kindgren *et al*, 2018). Besides, pervasive transcription from T-DNA-derived transcripts was shown to repress gene promoters (Nielsen *et al*, 2019). Different laboratories have contributed to the idea that upstream transcription has a negative impact on tandem downstream gene expression (Martens *et al*, 2004; Kim *et al*, 2012; Nguyen *et al*, 2014; Nielsen *et al*, 2019). In this study, we propose a contrasting model in which pervasive transcription induces downstream co-directional gene expression.

The model proposed in this study shares similarities with what was observed in RNA decay-deficient mutants. Many laboratories have shown that defective transcription termination leads to readthrough into downstream genes (Greger & Proudfoot, 1998; Vilborg *et al*, 2015; Baejen *et al*, 2017). Remarkably, in plants, readthroughs in the Rat1/Xrn2 homologue XRN3 mutant lead to the activation of downstream tandem genes. Notably, this was associated with enhanced Pol II occupancy and higher levels of H3K4me3 and H3K36me3 histone marks on the activated downstream genes (Krzyszton *et al*, 2018). An independent study confirmed this model by showing that the knockout of the upstream gene abolishes the positive effect of the readthrough on the downstream gene (Crisp *et al*, 2018). Moreover, the authors showed that the fold induction of the downstream gene was higher than the fold induction of the readthrough transcripts in the intergenic regions (Crisp *et al*, 2018). These works support the notion that upstream pervasive transcription enhances promoter-proximal pausing on the downstream gene. Here, we also observe higher Pol II density at the beginning of the *DOG1* gene as a result of *PUPPIES* overexpression (Fig 5A). We interpret this as stronger pausing of Pol II transcribing *DOG1* and unlikely to reflect *PUPPIES* transcription or termination over that region. This is because the primer used in targeted NET-seq for *DOG1* exon 1 is located before the 3’ss of *PUPPIES-fusion*, therefore not detecting this spliced isoform. In addition, when analysed in the chromatin fraction, unspliced *PUPPIES* constitute only a very small portion of the transcripts containing *DOG1* exon 1, suggesting that increased pausing at *DOG1* exon 1 cannot be attributed to *PUPPIES*-transcribing polymerases but rather represent the effect of *PUPPIES* on *DOG1*-transcribing polymerases kinetics (Appendix Fig S6A).

It is known that tandem genes have a high degree of co-expression (Hurst *et al*, 2004; Williams & Bowles, 2004; Chen *et al*, 2010). However, only recently the crosstalk between genes in co-directional gene pairs was explored at the transcriptional level. Nissani & Ulitsky, 2022, performed a computational analysis with gene expression datasets from the ENCODE project (Dunham *et al*, 2012; Luo *et al*, 2020a). The authors detected a strong accumulation of Pol II at the 5′ of downstream co-expressed tandem genes, as observed by us and Krzyszton *et al*, 2018. Strikingly, they found that treatment with splicing inhibitor Pladienolide B abolishes Pol II pausing on the downstream genes (Nissani & Ulitsky, 2022), suggesting the role of splicing in the readthrough-mediated activation of downstream gene expression. Fiszbein *et al* (2019) showed that splicing activates transcription from a TSS upstream of the 3’ss and induces Pol II accumulation shortly after the TSS. *PUPPIES* are spliced to a 3’ss immediately downstream of *DOG1* TSS. We speculate that splicing could be the factor driving the positive influence of *PUPPIES* on *DOG1* transcription and discriminate between the positive and negative effects of co-directional pervasive transcription globally. In future, it will be important to test the requirement of splicing for *PUPPIES*-mediated regulation of *DOG1* gene expression and to address whether the changes in transcriptional dynamics of the *DOG1* gene are driven by altered features of Pol II or chromatin.

## Materials and Methods

### Reagents and Tools table

| Reagent/Resource | Reference or Source | Identifier or Catalog Number |
|---|---|---|
| **Experimental Models** | | |
| *Arabidopsis thaliana dog1-3* | Nottingham Arabidopsis Stock Centre (NASC) | SALK_000867 |
| *Arabidopsis thaliana dog1-5* | Nottingham Arabidopsis Stock Centre (NASC) | SALK_022748 |
| *Arabidopsis thaliana puppies-1* | Nottingham Arabidopsis Stock Centre (NASC) | SALK_139540C |
| *pDOG1-LUC::DOG1* | Fedak *et al* (2016) | N/A |
| *psDOG1::LUC* | Fedak *et al* (2016) | N/A |
| **Oligonucleotides and sequence-based reagents** | | |
| Oligonucleotides | This study | Table EV3 |

 © 2023 The Authors

**Reagents and Tools table** (continued)

| Reagent/Resource | Reference or Source | Identifier or Catalog Number |
|---|---|---|
| **Recombinant DNA** | | |
| pHEE2E-TRI | Jin & Marquardt (2020) | Addgene Plasmid #71288 |
| pKI1.1R | Jin & Marquardt (2020) | Addgene Plasmid #85808 |
| pMOD_A0402 | Čermák *et al* (2017) | Addgene Plasmid #91009 |
| pMOD_B2303 | Čermák *et al* (2017) | Addgene Plasmid #91068 |
| pMOD_C3001 | Čermák *et al* (2017) | Addgene Plasmid #91094 |
| pTRANS_230 | Čermák *et al* (2017) | Addgene Plasmid #91118 |
| **Chemicals, enzymes and other reagents** | | |
| Acidic phenol-chloroform-isoamyl alcohol 125:24:1 pH 4.5 | ThermoFisher | AM9720 |
| AGAROSE | BioShop | AGA001.1 |
| Agencourt AMPure XP | Beckman Coulter | A63881 |
| Agencourt AMPure XP magnetic beads | Beckman Coulter | A63881 |
| All oligonucleotides (except stated otherwise) | Sigma-Aldrich | N/A |
| Beetle luciferin potassium salt | Promega | E1605 |
| Beta-mercaptoethanol | Sigma-Aldrich | M6250-100ML |
| Betaine 5M | Sigma-Aldrich | B0300-1VL |
| Chloroform | POCH | 234431116 |
| CloneJET PCR Cloning Kit | ThermoFisher | K1232 |
| COmplete protease inhibitors | Roche | 5056489001 |
| DAPI | Sigma-Aldrich | D9564-10MG |
| Dextran T40 | Sigma-Aldrich | 31389-500G |
| dNTP Mix 10 mM | ThermoFisher | R0192 |
| DTT | ThermoFisher | DTT001.10 |
| *E. coli* DNA Ligase | New England Biolabs | M0205L |
| *E. coli* DNA polymerase | New England Biolabs | M0209L |
| EDTA Sterile Solution 0.5 M pH 8.0 | BioShop | EDT111.500 |
| Empigen | Sigma-Aldrich | 30326-50ML |
| Ethanol 96 % | POCH | 396420113 |
| Ficoll 400 | BioShop | FIC400.100 |
| Formaldehyde | Sigma-Aldrich | F8775-4x25ML |
| Formamide | POCH | 432200116 |
| Glufosinate ammonium | Sigma-Aldrich | 45520-100MG |
| Glycerol | Chempur | 114433204 |
| HEPES | ACROS | FSBP31010 |
| Hygromycin | BioShop | HYG002 |
| Isopropanol | POCH | 751500111 |
| KCl | POCH | 739740114 |
| $KH_2PO_4$ | Sigma-Aldrich | P5655-100G |
| Mannitol | BioShop | MAN509.500 |
| Methanol | Chempur | 603-001-00-X |
| $MgCl_2$ | ThermoFisher | F 530-S |
| Miracloth | Sigma-Aldrich | 475855-1R |
| Murashige and Skoog (MS) medium | Sigma-Aldrich | M0404-10L |
| Murine RNase inhibitors | New England Biolabs | M0314L |
| *N,N*-Dimethylformamide | Sigma-Aldrich | 227056-250ML |

© 2023 The Authors

**Reagents and Tools table** (continued)

| Reagent/Resource | Reference or Source | Identifier or Catalog Number |
|---|---|---|
| $Na_2HPO_4 \cdot 7H_2O$ | Sigma-Aldrich | 30413-500G |
| NaCl | POCH | 794121116 |
| NEBNext Second Strand Synthesis (dNTP-free) Reaction Buffer | New England Biolabs | B6117S |
| NP-40 | BioShop | NON505.100 |
| Oligo(dT) | Sigma-Aldrich | N/A |
| PEG 6000 | Sigma-Aldrich | 8.07491.1000 |
| PEG 8000 50% | New England Biolabs | M0242S |
| Percoll | Sigma-Aldrich | P1644 |
| Phenol equilibrated stabilized | Applichem | A1153,0100 |
| Phusion High-Fidelity DNA Polymerase | ThermoFisher | F 530-S |
| Phusion High-Fidelity HF Buffer | ThermoFisher | F 530-S |
| Plant Agar | Duchefa | P1001.1000 |
| PMSF | Sigma-Aldrich | 93482-50ML-F |
| Q5 Hot Start High-Fidelity 2X Master Mix | New England Biolabs | M0049S |
| Qubit dsDNA HS | LifeTechnololies | Q32854 |
| Random hexamers | ThermoFisher | N8080127 |
| RiboLock 40 U/μl | ThermoFisher | EO0381 |
| RNase H | New England Biolabs | M0297-S |
| SDS | BDH Prolabo | 444464T |
| Sodium acetate | POCH | 805640115 |
| Spectinomycin | BioShop | SPE201.5 |
| Sucrose | CHEMPUR | 117720907 |
| SuperScript II | ThermoFisher | 18064014 |
| SuperScript III | ThermoFisher | 18064014 |
| SYBR Green mix | Roche | 4887352001 |
| T4 RNA ligase 2 Truncated 200 U/μl | New England Biolabs | M0242S |
| TRIS 1 M pH 7.5 | Bioshop | TRS111 |
| TRIS 1 M pH 8.0 | Bioshop | TRS222 |
| TRIS 1 M pH 8.5 | Bioshop | TRS333 |
| Triton X-100 | BioShop | TRX777.500 |
| TSO oligo | FUTURE Synthesis | N/A |
| TURBO DNase kit | Ambion | AM1907 |
| Urea | BioShop | URE001.500 |
| **Software** | | |
| PartSeg | Bokota *et al* (2021) | N/A |
| Olympus xCellence Software | Olympus | N/A |
| Napari | Sofroniew *et al* (2022) | N/A |
| ImageJ | https://imagej.nih.gov/ij/download.html | N/A |

## Methods and Protocols

### *Materials*

Materials used in this study, including reagents, plasmids and software, are listed in Table EV1. Oligonucleotides are listed in Table EV2.

### *Plant material*

*Arabidopsis thaliana* Col-0 was used as a WT for all experiments. Plants were grown in soil in a greenhouse under a long-day photoperiod (16 h light/8 h dark, 22°C/18°C). Seeds were harvested and stored in paper bags at room temperature. The *dog1-3* (SALK_000867), *dog1-5* (SALK_022748) and *puppies-1* (SALK_139540C)

 © 2023 The Authors

T-DNA insertion mutants were purchased from the Nottingham Arabidopsis Stock Centre (NASC). The sequences of the *DOG1* gene can be found in The Arabidopsis Information Resource (TAIR) database under the accession number AT5G45830. The DOG1-LUC reporter lines *pDOG1-LUC::DOG1* and *psDOG1::LUC* were generated before (Fedak *et al*, 2016).

For salt stress, freshly harvested seeds were sown on agar plates supplemented with different concentrations of NaCl, ranging from 50 to 200 mM. Plates were wrapped in aluminium foil and kept for 3 days at 4°C for cold stratification. After that, the material was collected or moved to a growth chamber under a long-day photoperiod for germination. Material collected for molecular analysis was obtained by flash-freezing the biological samples in liquid nitrogen and kept at −80°C.

#### Germination tests

Primary dormancy tests were performed for WT and mutants by sowing seeds on different days after harvest. Seeds were sown on half-strength MS agar plates and germinated under a long-day photoperiod. Pictures were taken each day using a high-resolution camera, and the appearance of root protrusion was used to count germination. Germination under salt stress was assessed by counting germinated seeds every day after cold stratification. Secondary dormancy treatment was performed as in Krzyszton *et al* (2022). Briefly, seeds were sown on plates with water-soaked blue paper and the plates were sealed with parafilm and incubated in the dark at 30°C for 4 h, 3 or 7 days before collecting material for expression analysis.

#### Quantification of luciferase activity

Quantification of LUC reporter expression was performed as in Kowalczyk *et al* (2017) with the following modifications. About 100 freshly harvested seeds of reporter lines were placed in wells of a white 96-well qPCR plate (Roche). Sixty microliter of water (mock) or water supplemented with different concentrations of NaCl, KCl, mannitol or PEG (treatment) was added to each well. Different concentrations of KCl, mannitol or PEG were used to induce osmotic potentials of −0.43, −0.64 and −0.86 MPa corresponding to 100, 150 and 200 mM of NaCl, respectively. Plates were covered with aluminium foil and kept for 2 days at 4°C. Then, the media were replaced by mock or treatment media supplemented with 1 mM beetle luciferin potassium salt. Plates were covered with aluminium foil and kept for one more day at 4°C. Before measuring the signal, 40 μl of excess media was discarded from the wells. The luminescence was measured using a NightSHADE camera (Berthold), with exposure times ranging from 20 to 30 min.

#### CRISPR-Cas9 mutant generation

The generation of the CRISPR-Cas9 mutant *puppies-ox* was performed following the protocol described by Jin & Marquardt (2020). Primers used for cloning are listed in Table EV2. T2 plants were confirmed to be Cas9-free by lack of RFP fluorescence and PCRs targeting the Cas9 and HygR genes on the transgene. The deletion range was determined by Sanger sequencing.

#### dCas9 mutant generation

The generation of the dCas9 transgenic plants was performed according to the detailed protocols 3B and 5 described by Čermák *et al* (2017). Two different sgRNAs were cloned in each construct. Three constructs were used for the transformation of WT plants. Primers used for cloning are listed in Table EV2. T1 transformant plants were selected by resistance to glufosinate ammonium. The seeds from the selected T1 plants were used for phenotypic analysis. Based on their phenotype, four independent transgenic lines were selected and propagated, and seeds from transgenic T2 plants were imbibed under 100 mM NaCl and used for expression analysis.

#### RNA extraction

RNA was extracted using the phenol–chloroform protocol. Seeds were ground to a fine powder while frozen using a plastic pellet pestle fitted in an electric drill. Seed material was mixed with 0.6 ml of RNA extraction buffer (100 mM Tris pH 8.5, 5 mM EDTA, 100 mM NaCl, 0.5% SDS, 1% beta-mercaptoethanol). Then, 0.6 ml of chloroform was added and samples were vortexed and centrifuged for 10 min at 14,000 *g* at 4°C. The supernatant was transferred to new tubes and 0.3 ml of phenol (pH 7.5–8.0) was added and samples were vortexed. Then, 0.3 ml of chloroform was added and samples were vortexed and centrifuged for 10 min at 14,000 *g* at 4°C. 0.5 ml of supernatant was transferred to new tubes and mixed with 0.5 ml of acidic phenol–chloroform–isoamyl alcohol 125:24:1 pH 4.5; samples were vortexed and centrifuged for 10 min at 14,000 *g* at 4°C. The last step was repeated once more. Then, the supernatant was mixed with 0.5 ml of chloroform and samples were vortexed and centrifuged for 10 min at 14,000 *g* at 4°C. Finally, the supernatant was mixed with 10% of the volume of 3 M sodium acetate (pH 5.2) and 80% of the volume of pure isopropanol and incubated for 20 min at −80°C. The RNA was pelleted by centrifuging for 30 min at 14,000 *g* at 4°C; the pellet was washed with 80% ethanol, dried and resuspended in Milli-Q water. DNase treatment of RNA samples was performed following the rigorous treatment from TURBO DNase protocol (ThermoFisher) with the following modifications: after adding DNase buffer to the RNA, the samples were mixed by pipetting up and down 10 times and centrifuged at 10,000 *g* for 2 min; and DNase incubation at 37°C was 30 + 20 min. RNA quality was assessed using agarose gel electrophoresis, Nanodrop 2000 spectrophotometer and PCR to test for genomic DNA contamination.

#### RT–qPCR

Reverse transcription (RT) was performed with SuperScript III according to the manufacturer's protocol using a mixture (1:1) of random hexamers and oligo(dT). qPCR was performed using a LightCycler 480 real-time system (Roche) with SYBR Green mix with primers listed in Table EV2. RT–qPCR results were normalized against the expression level of the housekeeping gene *UBC21* (AT5G25760; Czechowski *et al*, 2005). For absolute quantification, the target was PCR amplified and the amplicon was cloned in the pJet vector following the manufacturer's protocol (CloneJET PCR Cloning Kit). A series of 10-fold dilutions was used for qPCR to obtain standard curves for each primer.

#### 3'RNA-seq

3'RNA-seq was performed as described by Krzyszton *et al* (2022) using 500 ng of total RNA. Libraries were sequenced with Illumina NovaSeq 6000 in the Genomics Core Facility (Centre of New Technologies, University of Warsaw, Poland).

DEGs are defined as $|\log2\text{fold-change}| > \log2(1.5)$ and FDR $< 0.05$ and are listed in Dataset EV1. GO analysis was

performed using g:Profiler (https://biit.cs.ut.ee/gprofiler, Raudvere *et al*, 2019). Removal of redundant GO terms was done using the web server REVIGO (http://revigo.irb.hr, Supek *et al*, 2011). The full list of GO terms is provided in Dataset EV2.

#### 5'RACE-seq (rapid amplification of 5′ cDNA ends with high-throughput sequencing)

5'RACE-seq library preparation was based on nanoCAGE (Salimullah *et al*, 2011) and nanoPARE (Schon *et al*, 2018) procedures using template-switching RT. Five hundred nanogram of total RNA was mixed with 1 µl dNTP Mix 10 mM and 1 µl 6N_RT_TSO 50 µM and incubated at 72°C for 3 min and then put on ice. Then, the following reagents were added: 2 µl 5× First-Strand SuperScript II Buffer, 0.25 µl DTT 0.1 M, 1.8 µl $MgCl_2$ 50 mM, 2 µl Betaine 5 M, 0.5 µl TSO oligo 100 µM, 0.25 µl RiboLock 40 U/µl and 0.5 µl SuperSript II. The reaction was performed as follows: 25°C 5 min + 42°C 90 min + 10 cycles (50°C 2 min + 42°C 2 min) + 70°C 15 min + 4°C hold. Then, cDNA was purified using Agencourt AMPure XP magnetic beads and amplified in PCR (98°C 30 s + 10 cycles (98°C 10 s + 67°C 15 s + 72°C 1.5 min) + 72°C 10 min + 4°C hold) with Phusion using the TSO_n1 primer. The PCR product was purified using Agencourt AMPure XP and used as a template for second PCR (98°C 30 s + 30 cycles (98°C 10 s + 61°C 15 s + 72°C 30 s) + 72°C 10 min + 4°C hold) with Phusion using the TSO_n2 and *PUPPIES_*5RACE primers. The PCR product was purified using Agencourt AMPure XP and used as a template for third PCR (10 cycles) with Phusion using Illumina indexed primers. Concentration was checked by Qubit dsDNA HS (ThermoFisher). The libraries were pooled in equal amounts and sequenced with Illumina MiSeq in the Oligo facility (https://oligo.ibb.waw.pl). Reads were mapped to the genome using STAR (v2.7.8a; Dobin *et al*, 2013) and filtered based on UMIs using UMI-tools (v1.1.0; Smith *et al*, 2017). The position of the last 5′ nucleotide was extracted using bedtools (v2.30.0; Quinlan & Hall, 2010) and used for the pileup graphs.

#### 3'RACE-seq (rapid amplification of 3′ cDNA ends with high-throughput sequencing)

3'RACE-seq was performed based on the procedure described by Warkocki *et al* (2018). RA3_15N oligos were ligated to 3′ ends of RNA. One microgram of total RNA was denatured at 72°C for 3 min and put on ice. Then, the following reagents were added: 1.5 µl T4 ligation buffer 10×, 3 µl RA3_15N oligo 25 µM, 3.6 µl PEG 8000 50%, 0.3 µl RiboLock 40 U/µl and 1 µl T4 RNA ligase 2 Truncated 200 U/µl in 15 µl final volume. The ligation reaction was performed by incubating the samples for 1 h at 25 + 17°C overnight. RT was performed as follows: 15 µl ligation reaction was mixed with 3 µl RTP primer 20 µM, and 2 µl dNTP Mix 10 mM. Samples were incubated for 5 min at 65°C and then 10 min at 4°C. Then, 6 µl 5× First-Strand SuperScript III Buffer, 3 µl DTT 0.1 M, 0.5 µl RiboLock 40 U/µl, 1 µl SuperSript III was added and samples incubated for 45 min at 52°C and then 15 min at 70°C. cDNA was purified using Agencourt AMPure XP and used for 1$^{st}$ PCR (98°C 30 s + 15 cycles (98°C 10 s + 63°C 15 s + 72°C 35 s) + 72°C 10 min + 4°C hold) with Phusion using the PUPPIES_3RACE and mRTPXT primers. The PCR product was purified using Agencourt AMPure XP and used as a template for second PCR (98°C 30 s + 12 cycles (98°C 10 s + 61°C 15 s + 72°C 30 s) + 72°C 10 min + 4°C hold) with Phusion using the mXTf and mXTr primers. The PCR product was purified using Agencourt AMPure XP and used as a template for third PCR (10 cycles) with Phusion using Illumina indexing primers. Concentration was checked by Qubit dsDNA HS (ThermoFisher). The libraries were pooled in equal amounts and sequenced with Illumina MiSeq in the Oligo facility (https://oligo.ibb.waw.pl). Reads were mapped to the genome using STAR (v2.7.8a; Dobin *et al*, 2013) and filtered based on UMIs using UMI-tools (v1.1.0; Smith *et al*, 2017). The position of the last 3′ nucleotide was extracted using bedtools (v2.30.0; Quinlan & Hall, 2010) and used for the pileup graphs.

#### Extraction of chrRNA

One hundred milligram of seeds was grounded to a fine powder in liquid nitrogen and mixed with 20 ml of Honda Buffer (0.44 M sucrose, 20 mM HEPES-KOH pH 7.4, 1.25% Ficoll, 2.5% Dextran T40, 10 mM $MgCl_2$, 5 mM DTT, 0.5% Triton X-100, 1 mM PMSF, 10 mM beta-mercaptoethanol and 1 tablet/250 ml cOmplete protease inhibitors) for 10 min at 4°C. The homogenate was filtered through a double layer of Miracloth (Sigma). The flow-through was centrifuged at 2,000 *g* for 15 min at 4°C. The nuclei pellet was washed once with 1.5 ml of Honda Buffer supplemented with 15 U murine RNase inhibitors. The pellet was resuspended in 600 µl of Honda buffer and further purified on a Percoll density gradient as follows: On a 2 ml tube, 600 µl 40% Percoll in Honda buffer was gently placed on top of 600 µL of 75% Percoll in Honda buffer. The resuspended nuclei pellet was then placed on top of the Percoll gradient. Tubes were centrifuged at 10,000 *g* for 20 min at 4°C. Purified nuclei were obtained from the interface between the layers containing 40 and 75% Percoll. The nuclei were washed once more with Honda buffer. The pellet was then resuspended in 500 µl of cold glycerol buffer (50% glycerol, 20 mM Tris–HCl pH 8, 75 mM NaCl, 0.5 mM EDTA, 0.85 mM DTT, 1% Empigen, 10 mM beta-mercaptoethanol, 0.125 mM PMSF, 1 tablet/250 ml cOmplete protease inhibitor and 5 U murine RNase inhibitors) and overlaid on top of 500 µl of urea lysis buffer (10 mM HEPES-KOH pH 7.4, 7.5 mM $MgCl_2$, 0.2 mM EDTA, 300 mM NaCl, 1 M Urea, 1% NP-40, 10 mM beta-mercaptoethanol, 0.5 mM PMSF, 1 tablet/250 ml cOmplete protease inhibitor and 5 U murine RNase inhibitors). The samples were gently vortexed two times for 2 s, incubated on ice for 5 min and then centrifuged at 20,000 *g* for 2 min at 4°C. The chromatin pellet was washed twice with 600 µl of urea lysis buffer for 30 min at 4°C at 12 rpm in Rotator SB3 (Stuart). The washed chromatin pellet was then resuspended in 300 µl of RNA isolation buffer, and chrRNA was isolated with phenol–chloroform, followed by DNase treatment with TURBO DNase was performed as described previously.

#### Targeted NET-seq

The targeted NET-seq procedure is based on the SMIT assay (Oesterreich *et al*, 2016; Herz *et al*, 2019). Five hundred nanogram of chrRNA was used for 3′ end adapter ligation with RA3_15N oligos. Adapter ligation and RT were performed as described previously for 3'RACE-seq. cDNA was purified using Agencourt AMPure XP (1.6× the volume of the sample). Purified cDNA was used in the 1$^{st}$ PCR (15 cycles, 63°C annealing temperature and 30-s extension) using the primer RTP and primers spanning the *DOG1* gene listed in Table EV2. 1$^{st}$ PCR products were purified with Agencourt AMPure XP (1.8× the volume of the sample). The purified PCR products were used in the second PCR (10 cycles, 61°C annealing temperature and 30-s extension) using the primers RTP_XT and primers spanning the


© 2023 The Authors

*DOG1* gene with XT overhangs listed in Table EV2. The second PCR products were purified with Agencourt AMPure XP (1.4× the volume of the sample). The purified PCR products were used in the third PCR (10 cycles) using Illumina indexing primers. Libraries were purified with Agencourt AMPure XP (1.2× the volume of the sample). All PCRs were done using Phusion. Separate PCR reactions were performed for each primer. The concentration of final PCR products was checked by Qubit dsDNA HS (ThermoFisher). The libraries were pooled in equal amounts and sequenced with Illumina in the Oligo facility (https://oligo.ibb.waw.pl). FastQC was used for the initial quality-control analysis. Removal of adapter sequences and reads shorter than 30 nt was performed using cutadapt (v1.18; Martin, 2011). Reads were mapped to the genome using STAR (v2.7.8a; Dobin *et al*, 2013) and filtered based on UMIs using UMI-tools (v1.1.0; Smith *et al*, 2017). The position of the last 3′ nucleotide was extracted using bedtools (v2.30.0; Quinlan & Hall, 2010).

The nucleotide sequenced immediately after the adapter sequence corresponds to the 3′ end of the nascent RNA, which is the Pol II active site and therefore can be used to infer the position of a transcribing Pol II (Churchman & Weissman, 2011). However, the method also detects the 3′ ends of RNAs associated with the transcriptional machinery but not physically linked to Pol II such as intermediates of splicing after 5′ss cleavage (Prudêncio *et al*, 2020). Therefore, we removed reads in which the end position mapped precisely to a 5′ss of *DOG1*. Reads were also filtered to the genomic *DOG1 locus* and to reads which end is within 600 nt after the last nt of the sequencing primer. A table containing the number of reads with the same Pol II position and the corresponding genomic position was obtained. Pseudocounts were added, replacing the nt positions without any reads by the value 0.1. Then, we computed for each nt position, the ratio of the number of reads to the total number of reads from the same *DOG1* sequencing primer, for each biological replicate from WT and mutant. This normalization enables the interpretation of Pol II density regardless of the total number of transcribing Pol II so that a higher ratio of reads means that Pol II is observed more frequently at a certain position/region (interpreted as Pol II pausing or slowing down) rather than a generally higher number of transcribing Pol II. Then, data smoothing was performed using a rolling median of 11 nt. Followed by averaging the biological replicates for each region sequenced by the same *DOG1* primer. For statistical analysis, the data for each replicate were binned in 25 nt, and for each bin, a two-tailed *t*-test followed by Bonferroni correction was performed. The fold-change (*puppies-ox*/WT) was also calculated for 25 nt bins. All the codes for data processing and plotting are available on GitHub.

### smFISH

The set of smFISH probes was designed to target the full *shDOG1* sequence, including intron 1 using Stellaris Probe Designer version 4.2 (Biosearch Technologies). Including probes targeting *DOG1* intron enhances the chances of detecting signals from nascent *DOG1* RNA. Detection of only nascent *DOG1* RNA is probably impossible due to the short length and poor nt composition of the intron sequence. Each probe was labelled with the Quasar670 fluorophore. Probes sequences are listed in Table EV3. smFISH method is based on the procedure described by Duncan *et al* (2016) and adapted to *Arabidopsis* embryos with the following modifications. Seeds were imbibed for 3 days under salt stress as described previously. The seed coat was dissected under a binocular, and the embryo was isolated from the seed and quickly placed onto a drop of 4% formaldehyde on a microscope glass slide. Embryos were fixed for 30 min and then washed three times in 1× PBS. Permeabilization was achieved by air-drying the slides at room temperature for 1 h and then immersing them at 4°C in 70% ethanol overnight, followed by 2 h in pure methanol, and overnight in 70% ethanol.

The smFISH slides were imaged using a widefield inverted fluorescence microscope Olympus IX81 (Olympus), with a 100X UPLANSAPO oil-immersion objective (1.4 NA) and a Hamamatsu Orca-R2 (C10600) CCD camera. DAPI signal was acquired using a 387/11 excitation filter and 20 ms exposure time. The signal from *DOG1* probes labelled with Quasar670 was acquired using a 650/13 excitation and 1 s exposure time. 3D imaging was done by acquiring multiple optical slices spanning the entire cell. 25–40 *z*-slices were recorded with a 0.3 μm step size. For image acquisition, the xCellence software (Olympus) was used. For representative images shown in the figures, a maximum projection of the *z*-stack was obtained using ImageJ. The attribution of colours and cell segmentation on the maximum projection images was performed in Napari (Sofroniew *et al*, 2022).

We have limited our analysis to cells from the meristematic zone of the root because their shape, size and spatial architecture allow easy recognition and cell segmentation, and preservation of the tissue integrity in the squashed material. For image analyses, we used PartSeg, a novel computational tool for image processing, and segmentation described by Bokota *et al* (2021). Cells were segmented manually on one *z*-slice and projected to the *z*-slices above and below corresponding to the top and bottom edges of the cell. To each cell, a segmentation component is attributed, becoming a cell identifier. The area segmented as part of one cell cannot be part of a different cell, so no *foci* can be counted more than once. Nuclei segmentation was done using DAPI staining and give rise to three subcellular locations: nucleus, nuclear periphery and cytoplasm. Then, we developed the smFISH PartSeg plugin to identify, count and classify *foci* based on which cell component they belong to. The basis for *foci* segmentation in 3D was taken from FISH-quant v2 (Imbert *et al*, 2022) and adapted to the high background noise values in the embryo cells. Moreover, this plugin provides GUI and 2D/3D segmentation preview. First, we denoised image channels. Second, we normalized stack brightness based on the standard deviation of the denoised signal. And third, we applied a threshold-based identification algorithm to assign the *foci* to the segmentation components (cytoplasm, periphery and nucleus). Finally, a table is retrieved from the software with information relative to each of the identified *foci* regarding the genotype, embryo, cell and subcellular compartment they belong to and their fluorescence intensity.

For analysis of transcriptional burst size, we filtered *foci* present in the nucleus and nuclear periphery and discard the two brightest *foci* per cell, since up to two *foci* can correspond to transcription sites (TS) in diploid cells. From the remaining *foci*, we compute the average fluorescence intensity as a proxy for the intensity of one *DOG1* transcript. This was not done from cytoplasmic *foci* due to different background fluorescence in the nucleus and cytoplasm. Then, for all *foci* in the nucleus and nuclear periphery (including the two brightest *foci*), we compute the fold-change intensity to the computed average. *Foci* in which the fold-change was higher than or equal to 1.6 times the average were considered to be TS. This

© 2023 The Authors

threshold was selected based on the observations that the majority of the cells would have 1 or 2 TS. For cells with more than two *foci* with fold-change above the threshold, we considered the two brightest *foci* to be the two active TS. One limitation of this pipeline is that TS identification is only possible for cells with more than three nuclear *foci*. For analysis of active transcription, we divide the number of identified TS by the total number of alleles (two times the total number of cells).

#### *In silico* analysis

The coding/noncoding potential of *PUPPIES* transcripts was analysed using two different web tools, Coding Potential Calculator 2.0 (CPC 2.0) http://cpc2.cbi.pku.edu.cn (Kang *et al*, 2017) and Coding-NonCoding Identifying Tool (CNIT) http://cnit.noncode.org/CNIT/ (Guo *et al*, 2019). Both analyses were performed using the sequences of *PUPPIES-uns*, *PUPPIES-prom* with the inclusion of short alternative exon, *PUPPIES-prom*, the full genomic region of *DOG1* promoter, and *UBC21* as protein-coding gene control.

Prediction of nucleosome occupancy for *DOG1 locus* was performed based on *DOG1* genomic DNA sequence using the web server http://bio.physics.leidenuniv.nl/~noort/cgi-bin/nup3_st.py (van der Heijden *et al*, 2012).

MNase-seq data from (Data ref: Luo *et al*, 2020b) were used to map the nucleosome occupancy on the *DOG1 locus*.

All statistical analyses were performed using the R environment version 3.6.3. Sample sizes are mentioned in figure legends and/or displayed in plots as individual data points.

#### *Statistical analysis*

Statistical analyses were performed in RStudio. Sample sizes were not prespecified. The number of independent biological replicates ($n$) is mentioned in figure legends or displayed in the figures as data points. Information regarding the statistical test applied to each data is mentioned in the corresponding figure legend. Significant differences were accepted at $P$-values $< 0.05$.

## Data availability

All the codes for targeted NET-seq (https://github.com/Miguel-Montez/Targeted-NET-seq_analysis.git) and smFISH (https://github.com/Miguel-Montez/smFISH-data-analysis.git) data processing and plotting are publicly available on GitHub. The MNase-seq data used in this study were published previously (Data ref: Luo *et al*, 2020b) and can be found in the GEO database under the accession code GSE139465, https://www.ncbi.nlm.nih.gov/geo/query/acc.cgi?acc=GSE139465 (GSM4916341, https://www.ncbi.nlm.nih.gov/geo/query/acc.cgi?acc=GSM4916341). The 3’RNA-seq data generated for this study have been deposited at the Gene Expression Omnibus (GEO) under the accession code GSE208755 (https://www.ncbi.nlm.nih.gov/geo/query/acc.cgi?acc=GSE208755).

**Expanded View** for this article is available online.

### Acknowledgements

We thank Stefanie Rosa and Svenja Reeck for the help with the initial smFISH experiments, Lukasz Borowski and Aleksander Chlebowski for the assistance using the microscope facility, Tomasz W. Turowski for the support with NET-Seq analysis, Sebastian Marquardt for sharing vectors and protocols for CRISPR-Cas9 mutant generation, and Magdalena Boguta, Andrzej Jerzmanowski and Lien Brzezniak for the critical comments on the manuscript. We also thank all the members of our laboratory for the fruitful discussions throughout the entire duration of this work. This work was funded by the Foundation for Polish Science (FNP TEAM POIR.04.04.00-00-3C97/16) and by the Polish National Science Centre (NCN SONATA BIS UMO-2018/30/E/NZ1/00354) awarded to S.S. Miguel M. was supported by the Portuguese Fundação para a Ciência e a Tecnologia (FCT SFRH/BD/145264/2019) and the European Cooperation in Science and Technology (COST) short-term scientific mission (Action CA16212 INDEPTH). M. K. was supported by the Polish National Science Centre (NCN OPUS UMO-2021/41/B/NZ3/02605). D.P. research was funded by Warsaw University of Technology within the Excellence Initiative: Research University (IDUB) programme. D.P. and G.B. work was co-supported by the Polish National Science Centre (NCN 2019/35/O/ST6/02484 and 2020/37/B/NZ2/03757).

### Author contributions

**Miguel Montez:** Conceptualization; data curation; supervision; funding acquisition; investigation; methodology; writing – original draft; writing – review and editing. **Maria Majchrowska:** Investigation. **Michal Krzyszton:** Data curation; investigation. **Grzegorz Bokota:** Software. **Sebastian Sacharowski:** Investigation. **Magdalena Wrona:** Investigation. **Ruslan Yatusevich:** Investigation. **Ferran Massana:** Investigation. **Dariusz Plewczynski:** Software; supervision. **Szymon Swiezewski:** Supervision; funding acquisition; writing – original draft; writing – review and editing.

### Disclosure and competing interests statement

The authors declare that they have no conflict of interest.

## References

Abel GH, MacKenzie AJ (1964) Salt tolerance of soybean varieties (*Glycine max* L. Merrill) during germination and later growth. *Crop Sci* 4: 157–161

Alonso-Blanco C, Bentsink L, Hanhart CJ, Blankestijn-de Vries H, Koornneef M (2003) Analysis of natural allelic variation at seed dormancy loci of *Arabidopsis thaliana*. *Genetics* 164: 711–729

Apse MP, Aharon GS, Snedden WA, Blumwald E (1999) Salt tolerance conferred by overexpression of a vacuolar Na+/H+ Antiport in *Arabidopsis*. *Science* 285: 1256–1258

Argyris J, Dahal P, Hayashi E, Still DW, Bradford KJ (2008) Genetic variation for lettuce seed Thermoinhibition is associated with temperature-sensitive expression of Abscisic acid, gibberellin, and ethylene biosynthesis, metabolism, and response genes. *Plant Physiol* 148: 926–947

Ariel F, Jegu T, Latrasse D, Romero-Barrios N, Christ A, Benhamed M, Crespi M (2014) Noncoding transcription by alternative RNA polymerases dynamically regulates an auxin-driven chromatin loop. *Mol Cell* 55: 383–396

Baejen C, Andreani J, Torkler P, Battaglia S, Schwalb B, Lidschreiber M, Maier KC, Boltendahl A, Rus P, Esslinger S *et al* (2017) Genome-wide analysis of RNA polymerase II termination at protein-coding genes. *Mol Cell* 66: 38–49

Barrero JM, Piqueras P, González-Guzmán M, Serrano R, Rodríguez PL, Ponce MR, Micol JL (2005) A mutational analysis of the ABA1 gene of *Arabidopsis thaliana* highlights the involvement of ABA in vegetative development. *J Exp Bot* 56: 2071–2083

Bentsink L, Jowett J, Hanhart CJ, Koornneef M (2006) Cloning of DOG1, a quantitative trait locus controlling seed dormancy in *Arabidopsis*. *Proc Natl Acad Sci USA* 103: 17042–17047

 © 2023 The Authors

Bikard D, Jiang W, Samai P, Hochschild A, Zhang F, Marraffini LA (2013) Programmable repression and activation of bacterial gene expression using an engineered CRISPR-Cas system. *Nucleic Acids Res* 41: 7429 – 7437

Bokota G, Sroka J, Basu S, Das N, Trzaskoma P, Yushkevich Y, Grabowska A, Magalska A, Plewczynski D (2021) PartSeg: a tool for quantitative feature extraction from 3D microscopy images for dummies. *BMC Bioinformatics* 22: 1 – 15

Brinker M, Brosché M, Vinocur B, Abo-Ogiala A, Fayyaz P, Janz D, Ottow EA, Cullmann AD, Saborowski J, Kangasjärvi J *et al* (2010) Linking the salt transcriptome with physiological responses of a salt-resistant *Populus* species as a strategy to identify genes important for stress acclimation. *Plant Physiol* 154: 1697 – 1709

Butt H, Bazin J, Prasad KVSK, Awad N, Crespi M, Reddy ASN, Mahfouz MM (2022) The Rice Serine/arginine splicing factor RS33 regulates pre-mRNA splicing during abiotic stress responses. *Cell* 11: 1796

Cai L, Dalal CK, Elowitz MB (2008) Frequency-modulated nuclear localization bursts coordinate gene regulation. *Nature* 455: 485 – 490

Čermák T, Curtin SJ, Gil-Humanes J, Čegan R, Kono TJY, Konečná E, Belanto JJ, Starker CG, Mathre JW, Greenstein RL *et al* (2017) A multipurpose toolkit to enable advanced genome engineering in plants. *Plant Cell* 29: 1196 – 1217

Chen WH, de Meaux J, Lercher MJ (2010) Co-expression of neighbouring genes in *Arabidopsis*: separating chromatin effects from direct interactions. *BMC Genomics* 11: 1 – 8

Chen H, Ruan J, Chu P, Fu W, Liang Z, Li Y, Tong J, Xiao L, Liu J, Li C *et al* (2020) AtPER1 enhances primary seed dormancy and reduces seed germination by suppressing the ABA catabolism and GA biosynthesis in *Arabidopsis* seeds. *Plant J* 101: 310 – 323

Churchman LS, Weissman JS (2011) Nascent transcript sequencing visualizes transcription at nucleotide resolution. *Nature* 469: 368 – 373

Crisp PA, Smith AB, Ganguly DR, Murray KD, Eichten SR, Millar AA, Pogson BJ (2018) RNA polymerase II read-through promotes expression of neighboring genes in SAL1-PAP-XRN retrograde signaling. *Plant Physiol* 178: 1614 – 1630

Cyrek M, Fedak H, Ciesielski A, Guo Y, Sliwa A, Brzezniak L, Krzyczmonik K, Pietras Z, Kaczanowski S, Liu F *et al* (2015) Seed dormancy in *Arabidopsis* is controlled by alternative polyadenylation of DOG1. *Plant Physiol* 170: 947 – 955

Czechowski T, Stitt M, Altmann T, Udvardi MK, Scheible WR (2005) Genome-wide identification and testing of superior reference genes for transcript normalization in *Arabidopsis*. *Plant Physiol* 139: 5 – 17

Dobin A, Davis CA, Schlesinger F, Drenkow J, Zaleski C, Jha S, Batut P, Chaisson M, Gingeras TR (2013) STAR: ultrafast universal RNA-seq aligner. *Bioinformatics* 29: 15 – 21

Dolata J, Guo Y, Kolowerzo A, Smolinski D, Brzyzek G, Jarmolowski A, Swiezewski S (2015) NTR1 is required for transcription elongation checkpoints at alternative exons in *Arabidopsis*. *EMBO J* 34: 544 – 558

Dorone Y, Boeynaems S, Flores E, Jin B, Hateley S, Bossi F, Lazarus E, Pennington JG, Michiels E, de Decker M *et al* (2021) A prion-like protein regulator of seed germination undergoes hydration-dependent phase separation. *Cell* 184: 4284 – 4298

Duncan S, Rosa S (2017) Gaining insight into plant gene transcription using smFISH. *Transcription* 9: 166 – 170

Duncan S, Olsson TSG, Hartley M, Dean C, Rosa S (2016) A method for detecting single mRNA molecules in *Arabidopsis thaliana*. *Plant Methods* 12: 13

Dunham I, Kundaje A, Aldred SF, Collins PJ, Davis CA, Doyle F, Epstein CB, Frietze S, Harrow J, Kaul R *et al* (2012) An integrated encyclopedia of DNA elements in the human genome. *Nature* 489: 57 – 74

Fedak H, Palusinska M, Krzyczmonik K, Brzezniak L, Yatusevich R, Pietras Z, Kaczanowski S, Swiezewski S (2016) Control of seed dormancy in *Arabidopsis* by a cis-acting noncoding antisense transcript. *Proc Natl Acad Sci USA* 113: E7846 – E7855

Femino AM, Fay FS, Fogarty K, Singer RH (1998) Visualization of single RNA transcripts in situ. *Science* 280: 585 – 590

Finch-Savage WE, Footitt S (2017) Seed dormancy cycling and the regulation of dormancy mechanisms to time germination in variable field environments. *J Exp Bot* 68: 843 – 856

Fiszbein A, Krick KS, Begg BE, Burge CB (2019) Exon-mediated activation of transcription starts. *Cell* 179: 1551 – 1565

Giraudat J, Hauge BM, Valon C, Smalle J, Parcy F, Goodman HM (1992) Isolation of the *Arabidopsis* ABI3 gene by positional cloning. *Plant Cell* 4: 1251 – 1261

Gómez-Schiavon M, Chen LF, West AE, Buchler NE (2017) BayFish: Bayesian inference of transcription dynamics from population snapshots of single-molecule RNA FISH in single cells. *Genome Biol* 18: 164

Greger IH, Proudfoot NJ (1998) Poly(a) signals control both transcriptional termination and initiation between the tandem GAL10 and GAL7 genes of *Saccharomyces cerevisiae*. *EMBO J* 17: 4771 – 4779

Guo JC, Fang SS, Wu Y, Zhang JH, Chen Y, Liu J, Wu B, Wu JR, Li EM, Xu LY *et al* (2019) CNIT: a fast and accurate web tool for identifying protein-coding and long non-coding transcripts based on intrinsic sequence composition. *Nucleic Acids Res* 47: W516 – W522

Haslekås C, Stacy RAP, Nygaard V, Culiáñez-Macià FA, Aalen RB (1998) The expression of a peroxiredoxin antioxidant gene, AtPer1, in *Arabidopsis thaliana* is seed-specific and related to dormancy. *Plant Mol Biol* 36: 833 – 845

Heo JB, Sung S (2011) Vernalization-mediated epigenetic silencing by a long intronic noncoding RNA. *Science* 331: 76 – 79

Herz MAG, Kubaczka MG, Brzyżek G, Servi L, Krzyszton M, Simpson C, Brown J, Swiezewski S, Petrillo E, Kornblihtt AR (2019) Light regulates plant alternative splicing through the control of transcriptional elongation. *Mol Cell* 73: 1066 – 1074

Heydecker W, Higgins J, Gulliver RL (1973) Accelerated germination by osmotic seed treatment. *Nature* 246: 42 – 44

Hurst LD, Pál C, Lercher MJ (2004) The evolutionary dynamics of eukaryotic gene order. *Nat Rev Genet* 5: 299 – 310

Ietswaart R, Rosa S, Wu Z, Dean C, Howard M (2017) Cell-size-dependent transcription of FLC and its antisense long non-coding RNA COOLAIR explain cell-to-cell expression variation. *Cell Syst* 4: 622 – 635

Imbert A, Ouyang W, Safieddine A, Coleno E, Zimmer C, Bertrand E, Walter T, Mueller F (2022) FISH-quant v2: a scalable and modular tool for smFISH image analysis. *RNA* 28: 786 – 795

Jin Y, Marquardt S (2020) Dual sgRNA-based targeted deletion of large genomic regions and isolation of heritable Cas9-free mutants in *Arabidopsis*. *Bio Protoc* 10: e3796

Johnsson P, Ziegenhain C, Hartmanis L, Hendriks GJ, Hagemann-Jensen M, Reinius B, Sandberg R (2022) Transcriptional kinetics and molecular functions of long noncoding RNAs. *Nat Genet* 54: 306 – 317

Jones RA (1986) High salt tolerance potential in *Lycopersicon* species during germination. *Euphytica* 35: 575 – 582

Kang YJ, Yang DC, Kong L, Hou M, Meng YQ, Wei L, Gao G (2017) CPC2: a fast and accurate coding potential calculator based on sequence intrinsic features. *Nucleic Acids Res* 45: W12 – W16

Kim DH, Sung S (2017) Vernalization-triggered intragenic chromatin loop formation by long noncoding RNAs. *Dev Cell* 40: 302 – 312

Kim T, Xu Z, Clauder-Münster S, Steinmetz LM, Buratowski S (2012) Set3 HDAC mediates effects of overlapping noncoding transcription on gene induction kinetics. *Cell* 150: 1158 – 1169

Kindgren P, Ard R, Ivanov M, Marquardt S (2018) Transcriptional read-through of the long non-coding RNA SVALKA governs plant cold acclimation. *Nat Commun* 9: 1 – 11

Koornneef M, Jorna ML, Brinkhorst-van der Swan DLC, Karssen CM (1982) The isolation of abscisic acid (ABA) deficient mutants by selection of induced revertants in non-germinating gibberellin sensitive lines of *Arabidopsis thaliana* (L.) heynh. *Theor Appl Genet* 61: 385 – 393

Kowalczyk J, Palusinska M, Wroblewska-Swiniarska A, Pietras Z, Szewc L, Dolata J, Jarmolowski A, Swiezewski S (2017) Alternative polyadenylation of the sense transcript controls antisense transcription of DELAY OF GERMINATION 1 in *Arabidopsis*. *Mol Plant* 10: 1349 – 1352

Krzyszton M, Zakrzewska-Placzek M, Kwasnik A, Dojer N, Karlowski W, Kufel J (2018) Defective XRN3-mediated transcription termination in *Arabidopsis* affects the expression of protein-coding genes. *Plant J* 93: 1017 – 1031

Krzyszton M, Yatusevich R, Wrona M, Sacharowski SP, Adamska D, Swiezewski S (2022) Single seeds exhibit transcriptional heterogeneity during secondary dormancy induction. *Plant Physiol* 190: 211 – 225

Leymarie J, Robayo-Romero ME, Gendreau E, Benech-Arnold RL, Corbineau F (2008) Involvement of ABA in induction of secondary dormancy in barley (*Hordeum vulgare* L.) seeds. *Plant Cell Physiol* 49: 1830 – 1838

Lloret-Llinares M, Mapendano CK, Martlev LH, Lykke-Andersen S, Jensen TH (2016) Relationships between PROMPT and gene expression. *RNA Biol* 13: 6 – 14

Luo Y, Hitz BC, Gabdank I, Hilton JA, Kagda MS, Lam B, Myers Z, Sud P, Jou J, Lin K *et al* (2020a) New developments on the encyclopedia of DNA elements (ENCODE) data portal. *Nucleic Acids Res* 48: D882 – D889

Luo Y-X, Hou X-M, Zhang C-J, Tan L-M, Shao C-R, Lin R-N, Su Y-N, Cai X-W, Li L, Chen S *et al* (2020b) Gene Expression Omnibus GSE139460. (https://www.ncbi.nlm.nih.gov/geo/query/acc.cgi?acc=GSE139460). [DATASET]

Manfre AJ, LaHatte GA, Climer CR, Marcotte WR (2009) Seed dehydration and the establishment of desiccation tolerance during seed maturation is altered in the *Arabidopsis thaliana* mutant atem6-1. *Plant Cell Physiol* 50: 243 – 253

Marquardt S, Petrillo E, Manavella PA (2022) Cotranscriptional RNA processing and modification in plants. *Plant Cell* koac309 https://doi.org/10.1093/plcell/koac309

Martens JA, Laprade L, Winston F (2004) Intergenic transcription is required to repress the *Saccharomyces cerevisiae* SER3 gene. *Nature* 429: 571 – 574

Martin M (2011) Cutadapt removes adapter sequences from high-throughput sequencing reads. *EMBnet J* 17: 10

Mueller F, Senecal A, Tantale K, Marie-Nelly H, Ly N, Collin O, Basyuk E, Bertrand E, Darzacq X, Zimmer C (2013) FISH-quant: automatic counting of transcripts in 3D FISH images. *Nat Methods* 10: 277 – 278

Naftelberg S, Schor IE, Ast G, Kornblihtt AR (2015) Regulation of alternative splicing through coupling with transcription and chromatin structure. *Annu Rev Biochem* 84: 165 – 198

Nakabayashi K, Bartsch M, Xiang Y, Miatton E, Pellengahr S, Yano R, Seo M, Soppe WJJ (2012) The time required for dormancy release in *Arabidopsis* is determined by DELAY OF GERMINATION1 protein levels in freshly harvested seeds. *Plant Cell* 24: 2826 – 2838

Narsai R, Gouil Q, Secco D, Srivastava A, Karpievitch YV, Liew LC, Lister R, Lewsey MG, Whelan J (2017) Extensive transcriptomic and epigenomic remodelling occurs during *Arabidopsis thaliana* germination. *Genome Biol* 18: 1 – 18

Neugebauer KM (2002) On the importance of being co-transcriptional. *J Cell Sci* 115: 3865 – 3871

Nguyen T, Fischl H, Howe FS, Woloszczuk R, Barros AS, Xu Z, Brown D, Murray SC, Haenni S, Halstead JM *et al* (2014) Transcription mediated insulation and interference direct gene cluster expression switches. *Elife* 3: 1 – 21

Nielsen M, Ard R, Leng X, Ivanov M, Kindgren P, Pelechano V, Marquardt S (2019) Transcription-driven chromatin repression of intragenic transcription start sites. *PLoS Genet* 15: e1007969

Nissani N, Ulitsky I (2022) Unique features of transcription termination and initiation at closely spaced tandem human genes. *Mol Syst Biol* 18: e10682

Oesterreich FC, Herzel L, Straube K, Hujer K, Howard J, Neugebauer KM (2016) Splicing of nascent RNA coincides with intron exit from RNA polymerase II. *Cell* 165: 372 – 381

Park HY, Seok HY, Park BK, Kim SH, Goh CH, Lee BHA, Lee CH, Moon YH (2008) Overexpression of *Arabidopsis* ZEP enhances tolerance to osmotic stress. *Biochem Biophys Res Commun* 375: 80 – 85

Pelechano V, Wei W, Steinmetz LM (2013) Extensive transcriptional heterogeneity revealed by isoform profiling. *Nature* 497: 127 – 131

Piatek A, Ali Z, Baazim H, Li L, Abulfaraj A, Al-Shareef S, Aouida M, Mahfouz MM (2015) RNA-guided transcriptional regulation in planta via synthetic dCas9-based transcription factors. *Plant Biotechnol J* 13: 578 – 589

Prudêncio P, Rebelo K, Grosso AR, Martinho RG, Carmo-Fonseca M (2020) Analysis of mammalian native elongating transcript sequencing (mNET-seq) high-throughput data. *Methods* 178: 89 – 95

Qi LS, Larson MH, Gilbert LA, Doudna JA, Weissman JS, Arkin AP, Lim WA (2013) Repurposing CRISPR as an RNA-guided platform for sequence-specific control of gene expression. *Cell* 152: 1173 – 1183

Quesada V, García-Martínez S, Piqueras P, Ponce MR, Micol JL (2002) Genetic architecture of NaCl tolerance in *Arabidopsis*. *Plant Physiol* 130: 951 – 963

Quinlan AR, Hall IM (2010) BEDTools: a flexible suite of utilities for comparing genomic features. *Bioinformatics* 26: 841 – 842

Raj A, Peskin CS, Tranchina D, Vargas DY, Tyagi S (2006) Stochastic mRNA synthesis in mammalian cells. *PLoS Biol* 4: e309

Raj A, van den Bogaard P, Rifkin SA, van Oudenaarden A, Tyagi S (2008) Imaging individual mRNA molecules using multiple singly labeled probes. *Nat Methods* 5: 877 – 879

Raudvere U, Kolberg L, Kuzmin I, Arak T, Adler P, Peterson H, Vilo J (2019) G: profiler: a web server for functional enrichment analysis and conversions of gene lists (2019 update). *Nucleic Acids Res* 47: W191 – W198

Rosa S, Duncan S, Dean C (2016) Mutually exclusive sense–antisense transcription at FLC facilitates environmentally induced gene repression. *Nat Commun* 7: 1 – 7

Saldi T, Cortazar MA, Sheridan RM, Bentley DL (2016) Coupling of RNA polymerase II transcription elongation with pre-mRNA splicing. *J Mol Biol* 428: 2623 – 2635

Salimullah M, Mizuho S, Plessy C, Carninci P (2011) NanoCAGE: a high-resolution technique to discover and interrogate cells transcriptome. *Cold Spring Harb Protoc* 2011: pdb.prot5559

Schon MA, Kellner MJ, Plotnikova A, Hofmann F, Nodine MD (2018) NanoPARE: parallel analysis of RNA 5′ ends from low-input RNA. *Genome Res* 28: 1919 – 1930

Shearwin KE, Callen BP, Egan JB (2005) Transcriptional interference – a crash course. *Trends Genet* 21: 339 – 345

Smith T, Heger A, Sudbery I (2017) UMI-tools: modeling sequencing errors in unique molecular identifiers to improve quantification accuracy. *Genome Res* 27: 491 – 499

Sofroniew N, Lambert T, Evans K, Nunez-Iglesias J, Bokota G, Winston P, Peña-Castellanos G, Yamauchi K, Bussonnier M, Doncila Pop D *et al* (2022) Napari: a multi-dimensional image viewer for python. *Zenodo* https://doi.org/10.5281/ZENODO.6598542

 © 2023 The Authors

Sun L, Song G, Guo W, Wang W, Zhao H, Gao T, Lv Q, Yang X, Xu F, Dong Y *et al* (2019) Dynamic changes in genome-wide Histone3 Lysine27 Trimethylation and gene expression of soybean roots in response to salt stress. *Front Plant Sci* 10: 1031

Supek F, Bošnjak M, Škunca N, Šmuc T (2011) REVIGO summarizes and visualizes long lists of gene ontology terms. *PLoS ONE* 6: e21800

Suter DM, Molina N, Gatfield D, Schneider K, Schibler U, Naef F (2011) Mammalian genes are transcribed with widely different bursting kinetics. *Science* 332: 472 – 474

Swiezewski S, Liu F, Magusin A, Dean C (2009) Cold-induced silencing by long antisense transcripts of an *Arabidopsis* Polycomb target. *Nature* 462: 799 – 802

Thieffry A, Vigh ML, Bornholdt J, Ivanov M, Brodersen P, Sandelin A (2020) Characterization of *Arabidopsis thaliana* promoter Bidirectionality and antisense RNAs by inactivation of nuclear RNA decay pathways. *Plant Cell* 32: 1845 – 1867

Ungar IA (1978) Halophyte seed germination. *Bot Rev* 44: 233 – 264

Vallejo AJ, Yanovsky MJ, Botto JF (2010) Germination variation in *Arabidopsis thaliana* accessions under moderate osmotic and salt stresses. *Ann Bot* 106: 833 – 842

van der Heijden T, van Vugt JJFA, Logie C, van Noort J (2012) Sequence-based prediction of single nucleosome positioning and genome-wide nucleosome occupancy. *Proc Natl Acad Sci USA* 109: E2514 – E2522

Vilborg A, Steitz JA (2017) Readthrough transcription: how are DoGs made and what do they do? *RNA Biol* 14: 632 – 636

Vilborg A, Passarelli MC, Yario TA, Tycowski KT, Steitz JA (2015) Widespread inducible transcription downstream of human genes. *Mol Cell* 59: 449 – 461

Villa T, Porrua O, Villa CT, Porrua O (2022) Pervasive transcription: a controlled risk. *FEBS J* https://doi.org/10.1111/febs.16530

Wang Y, Fang Z, Yang L, Chan Z (2021) Transcriptional variation analysis of *Arabidopsis* ecotypes in response to drought and salt stresses dissects commonly regulated networks. *Physiol Plant* 172: 77 – 90

Waqas M, Korres NE, Khan MD, Nizami A-S, Deeba F, Ali I, Hussain H (2019) Advances in the concept and methods of seed priming. In *Priming and pretreatment of seeds and seedlings*, Hasanuzzaman M, Fotopoulos V (eds), pp 11 – 41. Singapore: Springer

Warkocki Z, Krawczyk PS, Adamska D, Bijata K, Garcia-Perez JL, Dziembowski A (2018) Uridylation by TUT4/7 restricts retrotransposition of human LINE-1 s. *Cell* 174: 1537 – 1548

Williams EJB, Bowles DJ (2004) Coexpression of neighboring genes in the genome of *Arabidopsis thaliana*. *Genome Res* 14: 1060 – 1067

Xiang Y, Nakabayashi K, Ding J, He F, Bentsink L, Soppe WJJ (2014) REDUCED DORMANCY5 encodes a protein phosphatase 2C that is required for seed DORMANCY in *Arabidopsis*. *Plant Cell* 26: 4362 – 4375

Yu X, Martin PGP, Michaels SD (2019) BORDER proteins protect expression of neighboring genes by promoting 3′ pol II pausing in plants. *Nat Commun* 10: 1 – 15

Zenklusen D, Larson DR, Singer RH (2008) Single-RNA counting reveals alternative modes of gene expression in yeast. *Nat Struct Mol Biol* 15: 1263 – 1271

Zhang HX, Blumwald E (2001) Transgenic salt-tolerant tomato plants accumulate salt in foliage but not in fruit. *Nat Biotechnol* 19: 765 – 768

Zhang HX, Hodson JN, Williams JP, Blumwald E (2001) Engineering salt-tolerant Brassica plants: characterization of yield and seed oil quality in transgenic plants with increased vacuolar sodium accumulation. *Proc Natl Acad Sci USA* 98: 12832 – 12836

Zhao X, Li J, Lian B, Gu H, Li Y, Qi Y (2018) Global identification of *Arabidopsis* lncRNAs reveals the regulation of MAF4 by a natural antisense RNA. *Nat Commun* 9: 1 – 12

**License:** This is an open access article under the terms of the Creative Commons Attribution License, which permits use, distribution and reproduction in any medium, provided the original work is properly cited.

# Expanded View Figures

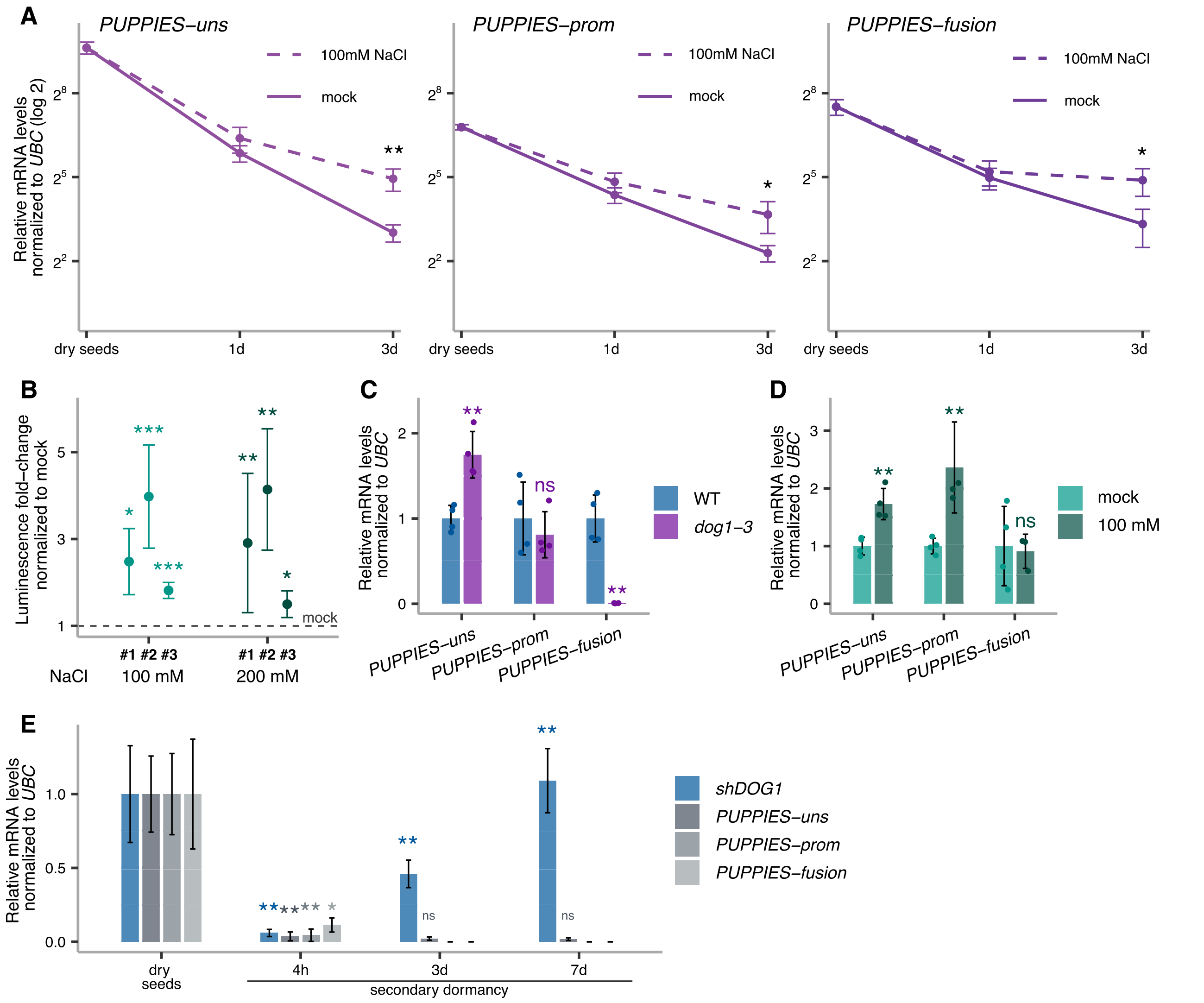


**Figure EV1. *PUPPIES* response to salt stress.**

- A Relative quantification of *PUPPIES-uns*, *PUPPIES-prom* and *PUPPIES-fusion* (from left to right) normalized to *UBC21* in dry seeds and seeds imbibed for 1 and 3 days in mock versus 100 mM NaCl. RT–qPCR points and error bars represent the mean ± SD. **P*-value < 0.05, ***P*-value < 0.01 from two-tailed Student's *t*-test.
- B Luciferase reporter assay. Plots represent the luminescence fold-change of seeds under 100 or 200 mM NaCl normalized to mock (horizontal dashed line) for three independent transgenic lines (#1, #2, #3) carrying the reporter construct *psDOG1::LUC*. Error bars represent the mean ± SD. **P*-value < 0.05, ***P*-value < 0.01, ****P*-value < 0.001 from paired Student's *t*-test comparing the raw luminescence levels in counts per second between mock and NaCl-treated samples.
- C RT–qPCR for *PUPPIES-uns*, *PUPPIES-prom* and *PUPPIES-fusion*. Expression in seeds treated with 100 mM NaCl in *dog1-3* relative to WT.
- D Expression in *dog1-3* mutant seeds treated with 100 mM NaCl relative to mock.
- E RT–qPCR for *shDOG1*, *PUPPIES-uns*, *PUPPIES-prom* and *PUPPIES-fusion* during heat stress induction of secondary dormancy of WT seeds. Bars and error bars represent the mean ± SD with $n = 3$. Expression levels normalized to *UBC21*. Expression levels of *PUPPIES-prom* and *PUPPIES-fusion* at 3 and 7 days were undetectable. Asterisks show the statistical significance for each transcript from the two-tailed Student's *t*-test for the comparison of each timepoint to its previous timepoint. ns *P*-value > 0.05, **P*-value < 0.05, ***P*-value < 0.01.

Data information: (C, D) Expression levels normalized to *UBC21*. Bars and error bars represent the mean ± SD. Points represent biological replicates. ns *P*-value > 0.05, ***P*-value < 0.01 from two-tailed Student's *t*-test.

 © 2023 The Authors

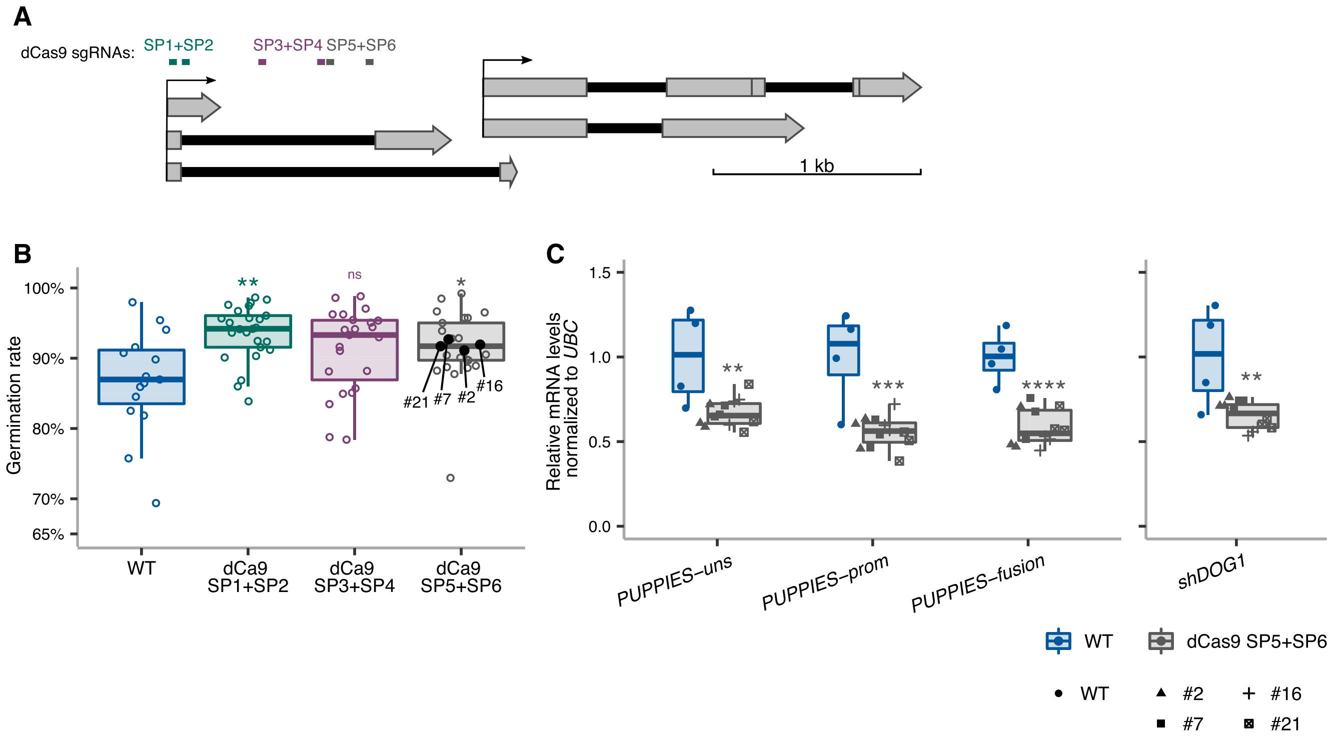


**Figure EV2. Blocking *PUPPIES* with dCas9.**

A Schematics of *DOG1 locus* with *PUPPIES* and *DOG1* TSS indicated by black arrows. Two sgRNAs per construct were introduced in WT targeting a ubiquitously expressed "dead" Cas9 protein (dCas9) to block *PUPPIES* transcription. The sgRNA positions are: SP1 + SP2 (in green), SP3 + SP4 (in purple), SP5 + SP6 (in grey).

B Box plots showing the percentage of germination in 150 mM NaCl at 4 days after stratification for WT (blue) and T2 seeds from selected transformants carrying dCas9 and the sgRNA pairs SP1 + SP2 (green), SP3 + SP4 (purple), SP5 + SP6 (grey). Points show the germination of seeds from each individual transgenic plant. Four independent SP5 + SP6 plants (#2, #7, #16, #21, represented by black points) with germination closest to the median were propagated and gene expression analyses (C) were carried out in the T3 seeds imbibed in the presence of 100 mM NaCl. Points represent biological replicates.

C Box plots show the expression levels measured by RT–qPCR of *PUPPIES-uns*, *PUPPIES-prom*, *PUPPIES-fusion* and *shDOG1* normalized to *UBC21* in the four different transgenic lines of SP5 + SP6 dCas9 (grey) relative to WT (blue). Points represent biological replicates.

Data information: (B, C) The box plot's central band marks the median, boxes mark the first and third quartiles, and whiskers extend the boxes to the largest value no further than 1.5 times the interquartile range. Statistical significance from two-tailed Student's *t*-test. ns *P*-value > 0.05, **P*-value < 0.05, ***P*-value < 0.01, ****P*-value < 0.001, *****P*-value < 0.0001.

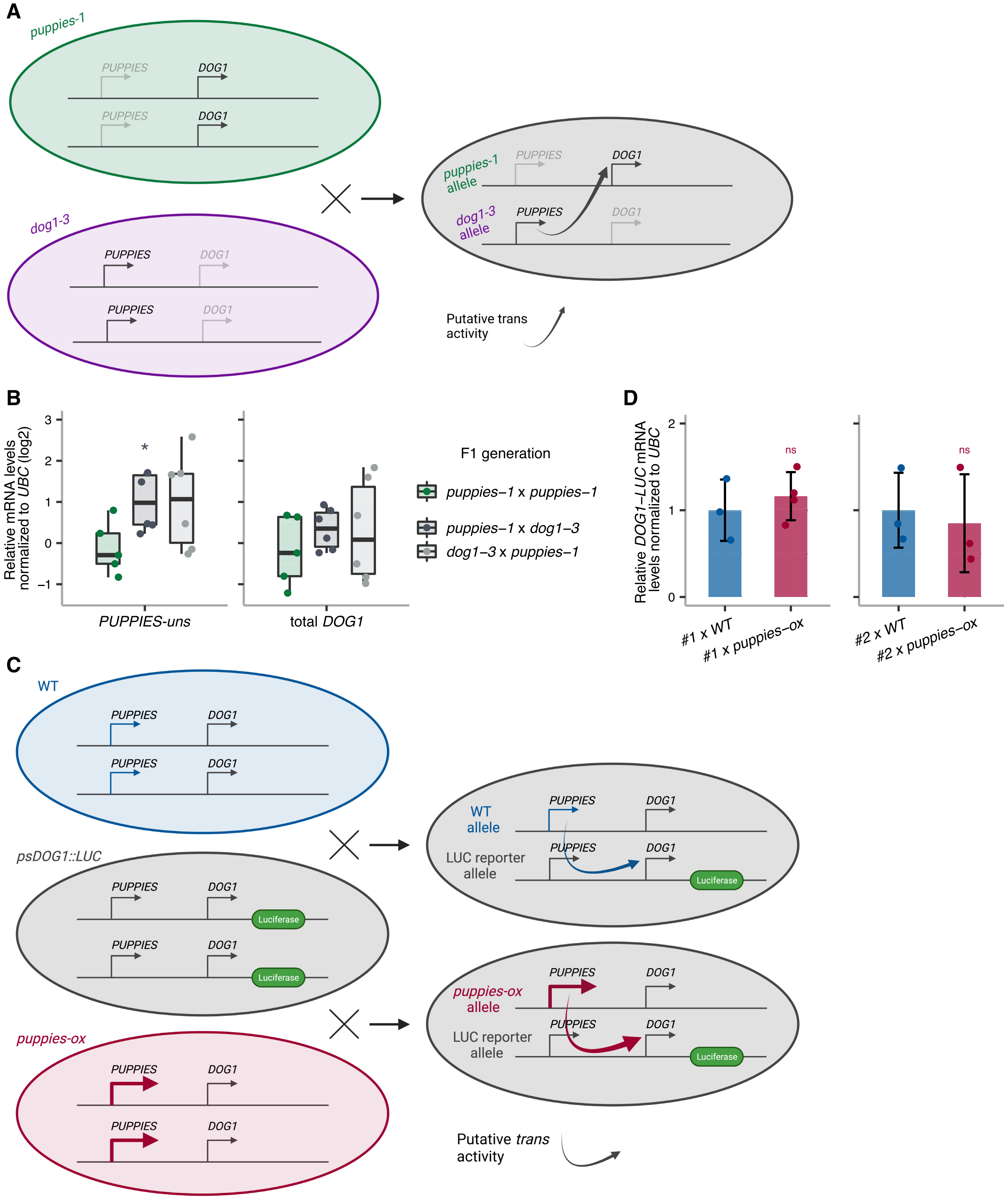


**Figure EV3.**

 © 2023 The Authors

◀

**Figure EV3.** ***PUPPIES* do not activate *DOG1* expression in *trans*.**

A Schematic representation of the cross between both diploid homozygous *puppies-1* and *dog1-3* mutants. The heterozygous F1 is used to test if supplying *PUPPIES* expression (from the *dog1-3* allele) induces *DOG1* expression (from the *puppies-1* allele) compared with the homozygous *puppies-1*.

B RT–qPCR for *PUPPIES-uns* and total *DOG1* normalized to *UBC21* in F1 seeds imbibed in the presence of 100 mM NaCl. *y*-axis in logarithmic scale. Points represent biological replicates. The box plot's central band marks the median, boxes mark the first and third quartiles, and whiskers extend the boxes to the largest value no further than 1.5 times the interquartile range. Statistical significance from two-tailed Student's *t*-test. **P*-value < 0.05.

C Schematic representation of the crosses between *psDOG1::LUC* and either WT or *puppies-ox*. The heterozygous F1 from *psDOG1::LUC* crossed with *puppies-ox* is used to test if supplying *PUPPIES* expression (from the *puppies-ox* allele) induces *DOG1-LUC* expression (from the reporter allele) relative to the levels of *DOG1-LUC* in the *psDOG1::LUC* crossed with WT.

D RT–qPCR with primers specific for *DOG1-LUC* in F1 seeds of two independent *psDOG1::LUC* lines (#1 and #2) crossed with either WT or *puppies-ox* imbibed in the presence of 100 mM NaCl. Expression was normalized to *UBC21*. Bars and error bars represent the mean ± SD. Points represent biological replicates. ns *P*-value > 0.05 from two-tailed Student's *t*-test.

**Figure EV4.** ***DOG1* single-molecule RNA FISH.**

▶

A Single *z*-section image of smFISH for *DOG1* in embryo cells from imbibed seeds. Separate DAPI (blue) and *DOG1* (grey) channels are shown on the left and middle images. A merged image is shown on the right. Manual segmentation (green outline) of the cells is performed based on a certain level of background fluorescence visible on the *DOG1* channel. Arrowhead points to the *focus* corresponding to the transcription site (TS). For each cell, the segmented area is projected to the *z*-sections above and below corresponding, respectively, to the top and bottom edges of the cell. Scale bar is 5 μm.

B After the projection of the cell segmentation across the *z*-stack, the image is denoised in each *z*-section. A maximum projection of the denoised *z*-stack is then performed to obtain a representative 2D image (C). The denoised *z*-stack is also used for the reconstruction of the cell and *foci* detection in 3D (see Materials and Methods section).

C Arrowhead points to the *focus* corresponding to the TS. Scale bar is 2 μm.

D, E *z*-stack max-projection images of *DOG1* smFISH in seeds imbibed in 100 mM NaCl of WT (left) versus *dog1-3* (right). The scale bar is 5 μm. Please note that WT picture in panel E is the same representative picture as used in Fig 4G.

F *z*-stack max-projection images of *DOG1* smFISH in WT without (left) versus with (right) RNase A treatment before hybridization. The scale bar is 5 μm.

G *z*-stack max-projection images of *DOG1* smFISH in seeds imbibed in 100 mM NaCl of WT (left) versus *puppies-1* (right). The scale bar is 5 μm.

H Comparison of fold-change of *DOG1* expression from RT–qPCR (left) with cytoplasmic *DOG1 foci* number per cell from smFISH (right; replotted data shown in Fig 4E) in *puppies-1* relative to WT. Points and error bars represent the mean ± SD. Diamond-shaped points in the RT–qPCR plot represent a pool of seeds collected from five plants, and diamond-shaped points in the smFISH plot represent a single embryo. Statistical significance from two-tailed Student's *t*-test. **P*-value < 0.05, *****P*-value < 0.0001.

I Distribution of intensities of cytoplasmic *DOG1 foci* in WT and *puppies-1* in arbitrary units (a.u.).

J *z*-stack max-projection images of *DOG1* smFISH in seeds imbibed in 100 mM NaCl of WT (left) versus *puppies-ox* (right). The scale bar is 5 μm.

K RT–qPCR quantification of 3′ end and 5′ end of *shDOG1* on nascent RNA from seeds imbibed in 100 mM NaCl from WT, *puppies-ox* and *puppies-1*. The plot shows the ratio of 3′ end to 5′ end relative to WT. Nascent RNA levels were normalized to *UBC21*. Bars and error bars represent the mean ± SD. Statistical significance from two-tailed Student's *t*-test. ns *P*-value > 0.05.

© 2023 The Authors

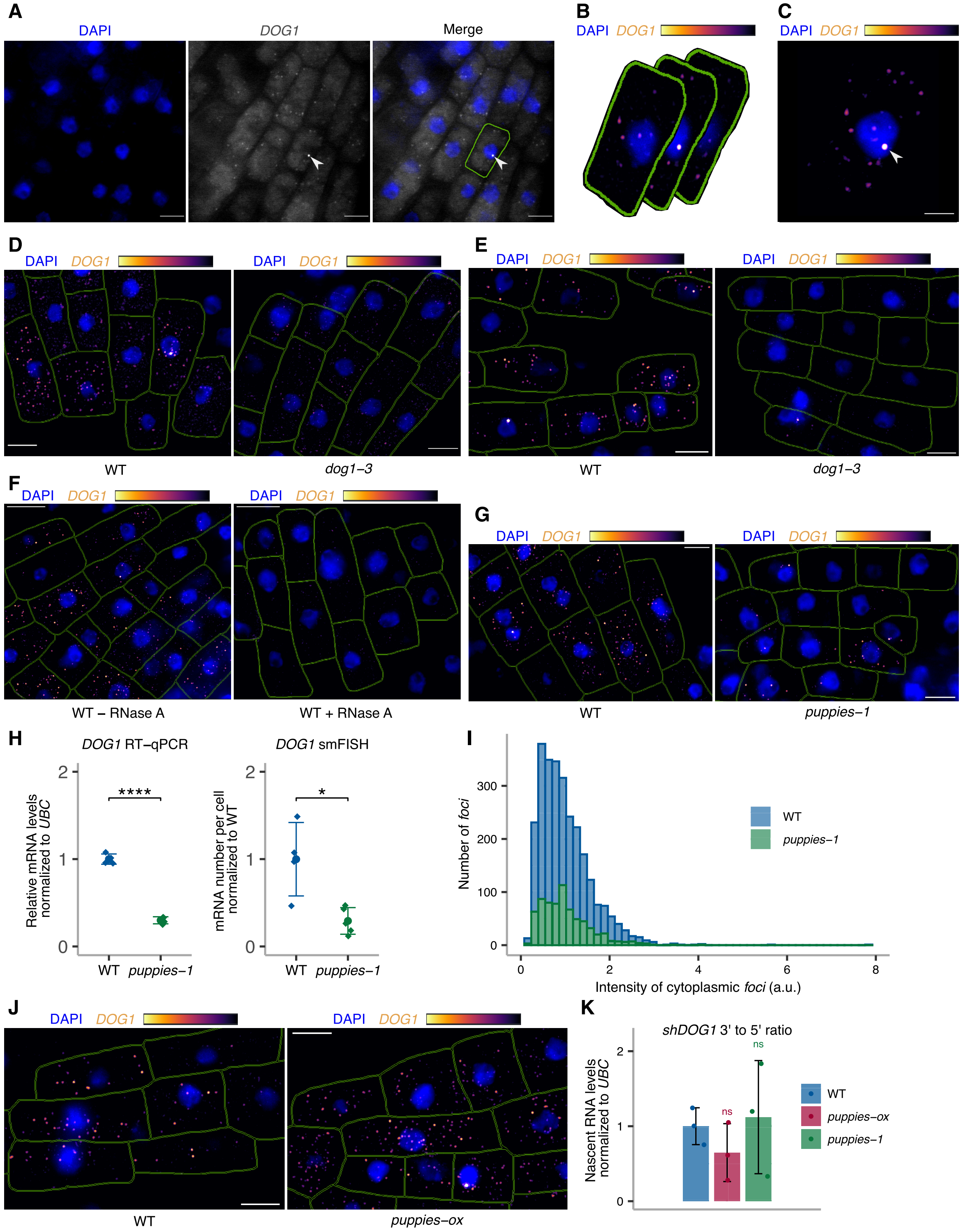


**Figure EV4.**

 © 2023 The Authors

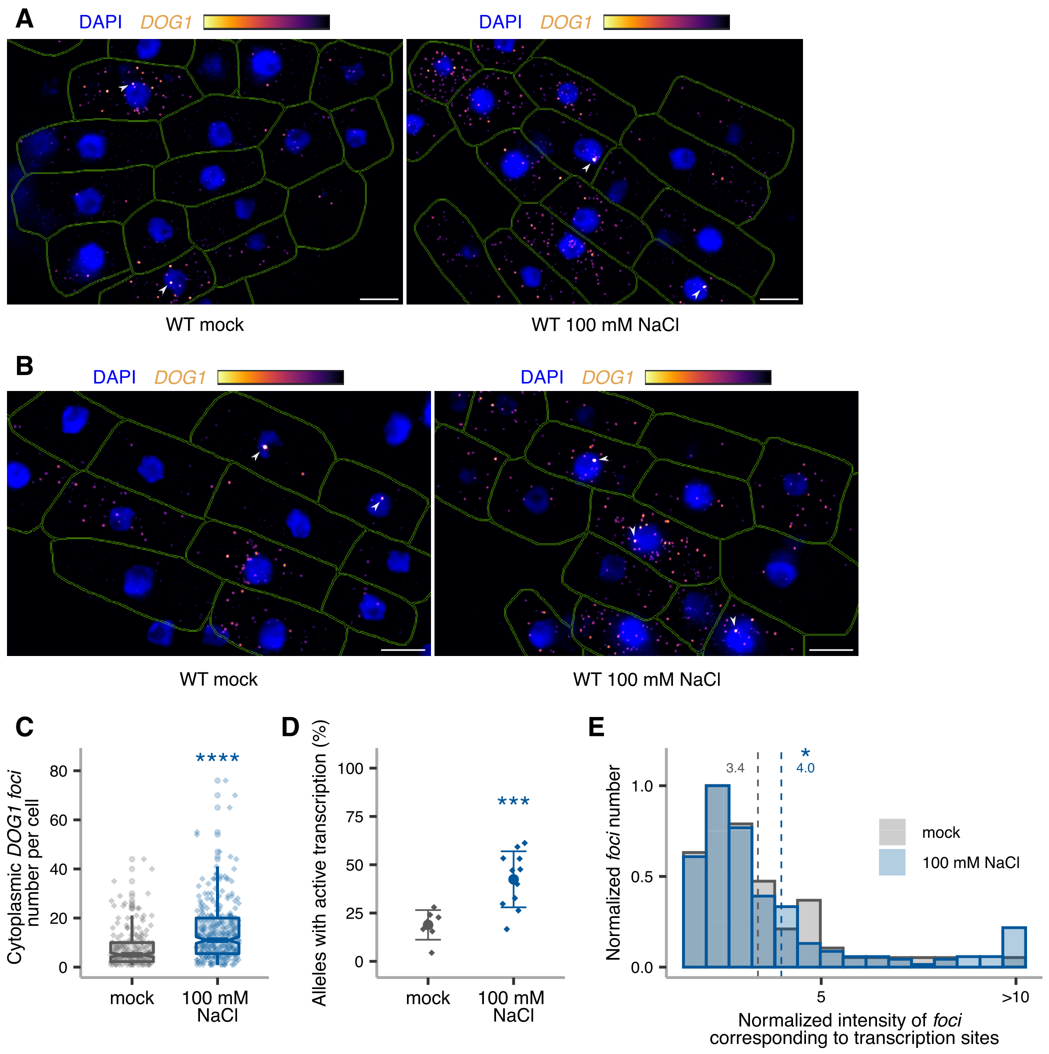


**Figure EV5. *DOG1* single-molecule RNA FISH in response to salt stress.**

A, B *z*-stack max-projection images of *DOG1* smFISH in WT embryos from imbibed seeds in mock (left) versus 100 mM NaCl (right). Arrowheads point to *foci* corresponding to the transcription sites (TS). The scale bar is 5 µm.

C Notched box plots showing the cytoplasmic *DOG1 foci* number per cell in mock versus salt treatment. Diamond-shaped points represent each cell, $n = 208$ cells from mock and $n = 309$ cells from NaCl-treated. The box plot's central band marks the median, lower and upper box limits mark the first and third quartiles, whiskers extend the boxes to the largest value no further than 1.5 times the interquartile range, and the notches extend to 1.58 times the interquartile range divided by sqrt ($n$).

D Plot showing the frequency of alleles with detected *foci* corresponding to *DOG1* transcription sites for mock versus NaCl-treated. Points and error bars represent the mean ± SD. Diamond-shaped points represent single embryos.

E Distribution of intensities of *foci* corresponding to *DOG1* TS in mock and NaCl-treated. In the *x*-axis is the fluorescence intensity fold-change of *foci* classified as TS to the average intensity of nuclear *foci*. In the *y*-axis is the number of *foci* normalized to their maximum value. Vertical dashed lines indicate the average fluorescence fold-change for mock in green (3.4) and NaCl-treated in blue (4.0). $n = 47$ *foci* from mock and $n = 159$ *foci* from NaCl-treated.

Data information: (C–E) Statistical significance from two-tailed Student's *t*-test. *$P$-value < 0.05, ***$P$-value < 0.001, ****$P$-value < 0.0001.

© 2023 The Authors

# Appendix for

Promoter-pervasive transcription causes Pol II pausing to boost *DOG1* expression in response to salt

Miguel Montez[1*], Maria Majchrowska[1], Michal Krzyszton[1], Grzegorz Bokota[2], Sebastian Sacharowski[1], Magdalena Wrona[1], Ruslan Yatusevich[1], Ferran Massana[1], Dariusz Plewczynski[2, 3] & Szymon Swiezewski[1*]

[1] Laboratory of Seeds Molecular Biology, Institute of Biochemistry and Biophysics, Polish Academy of Sciences, Pawinskiego 5a, 02-106, Warsaw, Poland.

[2] Laboratory of Functional and Structural Genomics, Centre of New Technologies, University of Warsaw, Banacha 2c, 02-097, Warsaw, Poland.

[3] Laboratory of Bioinformatics and Computational Genomics, Faculty of Mathematics and Information Science, Warsaw University of Technology, Koszykowa 75, 00-662, Warsaw, Poland.

[*] Co-corresponding authors

## Table of Contents

| Figure | Page |
| --- | --- |
| Appendix Figure S1 | 2 |
| Appendix Figure S2 | 2 |
| Appendix Figure S3 | 3 |
| Appendix Figure S4 | 4 |
| Appendix Figure S5 | 5 |
| Appendix Figure S6 | 6 |

## Appendix Figure S1

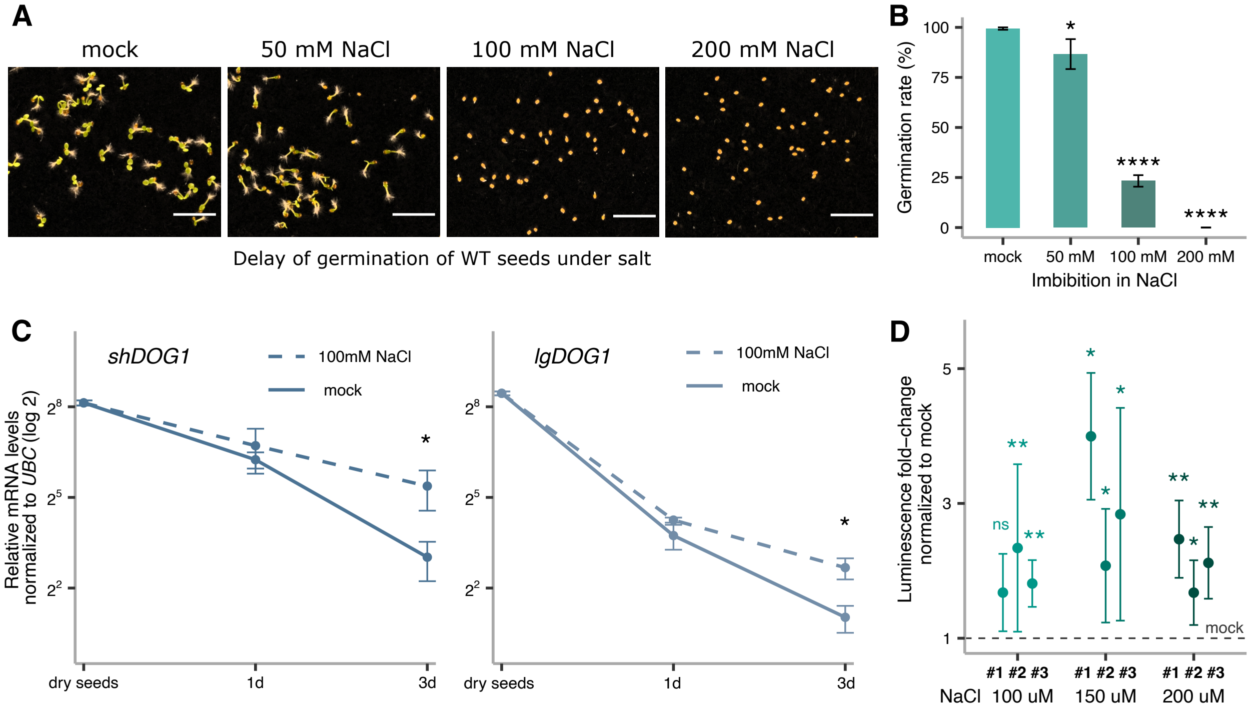


**Appendix Figure S1 - *DOG1* expression and seed germination under salt stress**

A Germination of WT seeds in different concentrations of NaCl and mock. Pictures were taken 3 days after stratification. Scale bars represent 5 mm.

B Quantification of germinated seeds 3 days after stratification. Bars and error bars represent the mean ± SD. * p-value < 0.05, **** p-value < 0.0001 from two-tailed Student's *t*-test.

C RT-qPCR relative quantification plots for *shDOG1* (left) and *lgDOG1* (right) normalized to *UBC21* (AT5G25760) in dry seeds and seeds imbibed for 1 day (1d) and 3 days (3d) in the absence or presence of 100 mM NaCl. Points and error bars represent the mean ± SD. * p-value < 0.05, **** p-value < 0.0001 from two-tailed Student's *t*-test.

D Luciferase reporter assay. The plot represents the luminescence fold-change of seeds under 100, 150, and 200 mM NaCl relative to mock (horizontal line) for 3 independent transgenic lines (#1, #2, #3) carrying the reporter construct *pDOG1-LUC::DOG1*. * p-value < 0.05, ** p-value < 0.01 from paired Student's *t*-test comparing the raw luminescence levels in counts per second between mock and NaCl-treated samples.

## Appendix Figure S2

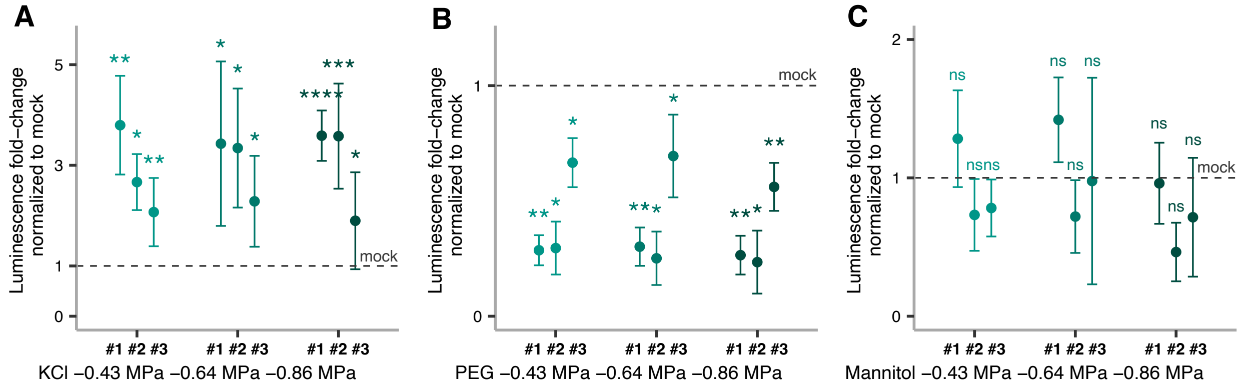


**Appendix Figure S2 - Induction of *DOG1* gene expression upon ionic stress**

A-C Luciferase reporter assays. Plots represent the changes in the luminescence of seeds caused by KCl (A), PEG (B), and mannitol (C), compared to mock (horizontal line). The osmotic pressures used are equivalent to the osmotic pressures caused by 100, 150, and 200 mM of NaCl. Three independent transgenic lines (#1, #2, #3) carrying the reporter construct *pDOG1-LUC::DOG1* were used. * p-value < 0.05, ** p-value < 0.01 from paired Student's *t*-test comparing the raw luminescence levels in counts per second between mock and treated samples.

## Appendix Figure S3

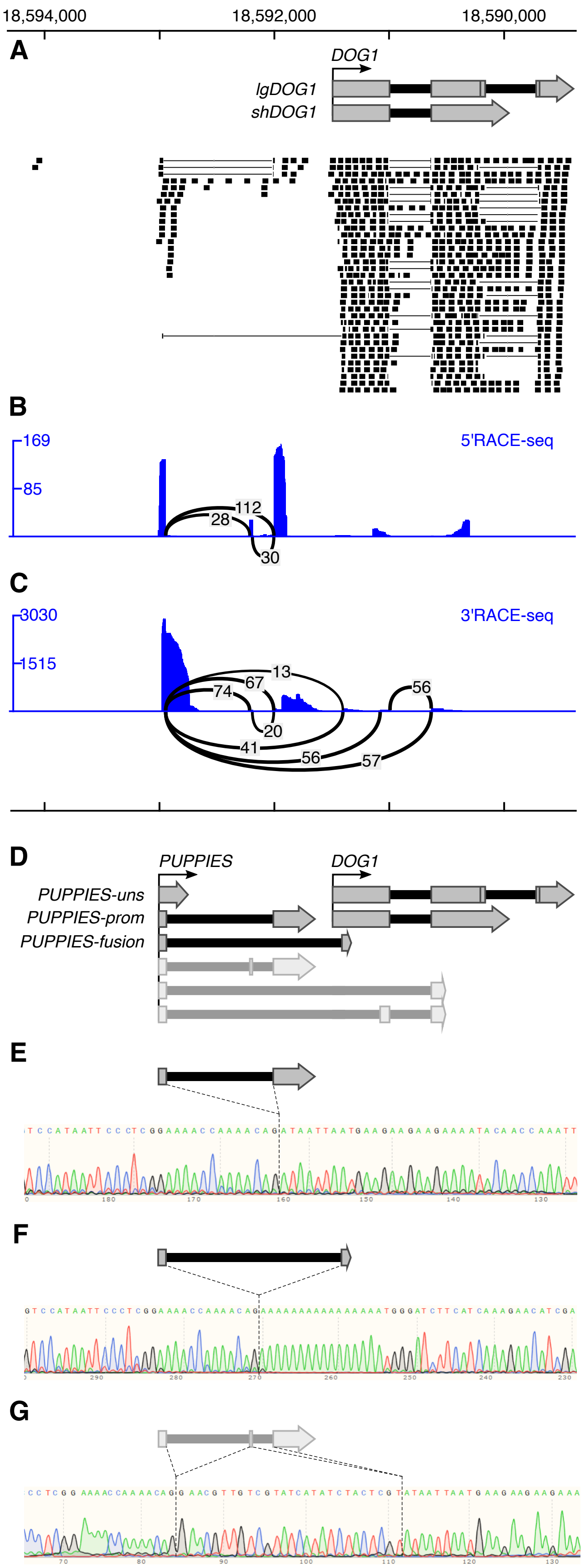


**Appendix Figure S3 - Splicing of *PUPPIES* transcript isoforms**

A Reads mapped to *DOG1 locus* from RNA-seq in seeds (Narsai *et al.*, 2017), showing reads for the *PUPPIES* region. Above is shown a horizontal line giving the chromosome coordinates and a schematic representation of the annotated transcripts from the *DOG1 locus*.

B Sashimi plot from 5´RACE-seq.

C Sashimi plot from 3´RACE-seq.

D Schematic representation of all newly annotated *PUPPIES* transcripts based on our results from 3'RNA-seq, 5' and 3' RACE-seq. In grey are the *PUPPIES* isoforms not quantified using RT-qPCR.

E-G Sanger sequencing of products obtained by PCR amplification of cDNA using primers for *PUPPIES* region. Chromatograms showing splicing of *PUPPIES-prom* isoform (E), splicing of *PUPPIES-fusion* isoform (F), and alternative splicing of *PUPPIES-prom* isoform with the inclusion of a short alternative exon (G).

## Appendix Figure S4

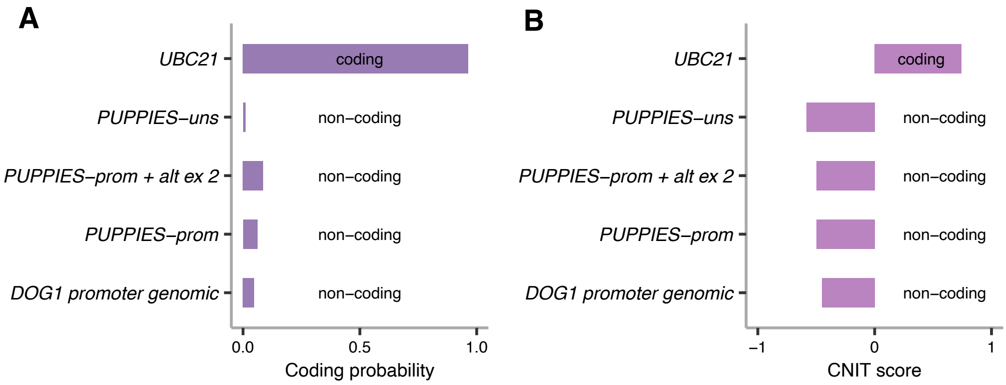


**Appendix Figure S4 - Coding/non-coding potential of *PUPPIES* RNA transcripts**

A Horizontal bar plot shows the coding probability from the online tool Coding Potential Calculator 2.0 (CPC 2.0; Kang *et al*., 2017).

B Horizontal bar plot shows the CNIT score for coding potential from the online tool Coding-NonCoding Identifying Tool (CNIT; Guo *et al*., 2019).

A, B Analyses were performed using the sequences of *PUPPIES-uns*, *PUPPIES-prom* with the inclusion of short alternative exon, *PUPPIES-prom*, the full genomic region of *DOG1* promoter, and *UBC21* as protein-coding gene control. Both CPC 2.0 and CNIT attribute a coding or non-coding label for each sequence analyzed as annotated on the plots.

# Appendix Figure S5

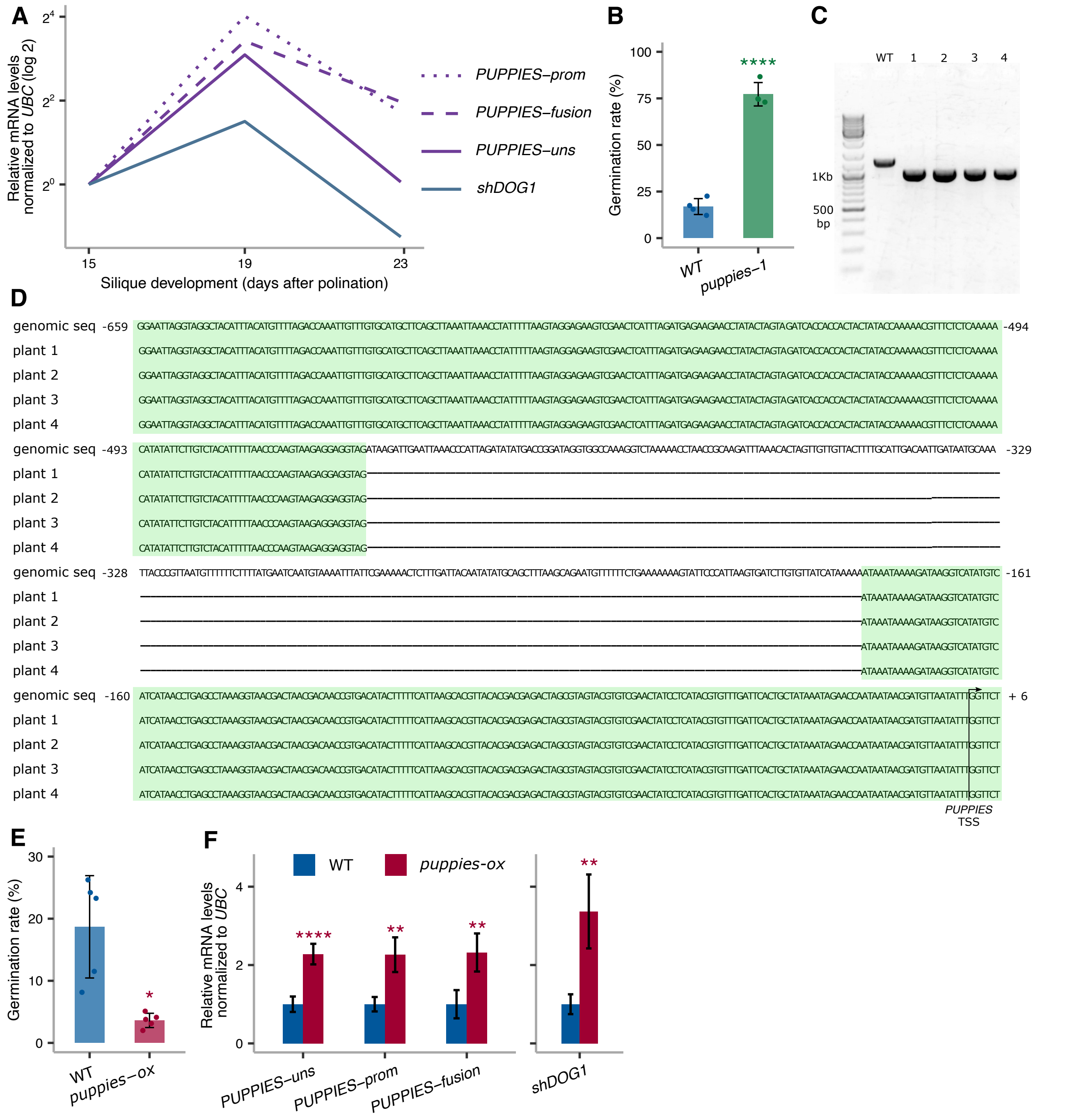


**Appendix Figure S5 - *PUPPIES* contribution to primary dormancy strength**

A Temporal expression patterns of *PUPPIES-prom*, *PUPPIES-fusion*, *PUPPIES-uns*, and *shDOG1*, during seed maturation (15, 19, and 23 days after pollination). RT-qPCR expression levels were normalized to *UBC21* and related to the first time point.

B Germination rate of seeds from WT and *puppies-1* one week after harvest (without stratification). Germination was counted 3 days after sowing.

C PCR amplification of genomic DNA from WT (lane 2) and 4 different T2 CRISPR-Cas9 plants (lanes 3 to 6) with primers flanking the deletion region. 1Kb Plus gel ladder (lane 1).

D Alignment of the results from Sanger sequencing of PCR products from 4 different T2 CRISPR-Cas9 plants to the genomic sequence of WT. Green boxes represent the sequences matching the genomic reference, while horizontal dashed lines represent the sequence deleted. Distance is given in bp as a relative to *PUPPIES* TSS (arrow).

E Germination rate of seeds from WT (blue) and *puppies-ox* (red) 3 weeks after harvest (without stratification).

B, E Bars and error bars represent the mean ± SD. Points represent individual biological replicates. * p-value < 0.05, **** p-value < 0.0001 from two-tailed Student's *t*-test.

F RT-qPCR expression fold-change of *PUPPIES-uns*, *PUPPIES-prom*, *PUPPIES-fusion*, and *shDOG1* in *puppies-ox* (red) relative to WT (blue) in maturing seeds from siliques 23 days after pollination. Bars and error bars represent the mean ± SD. ** p-value < 0.01, **** p-value < 0.0001 from two-tailed Student's *t*-test.

## Appendix Figure S6

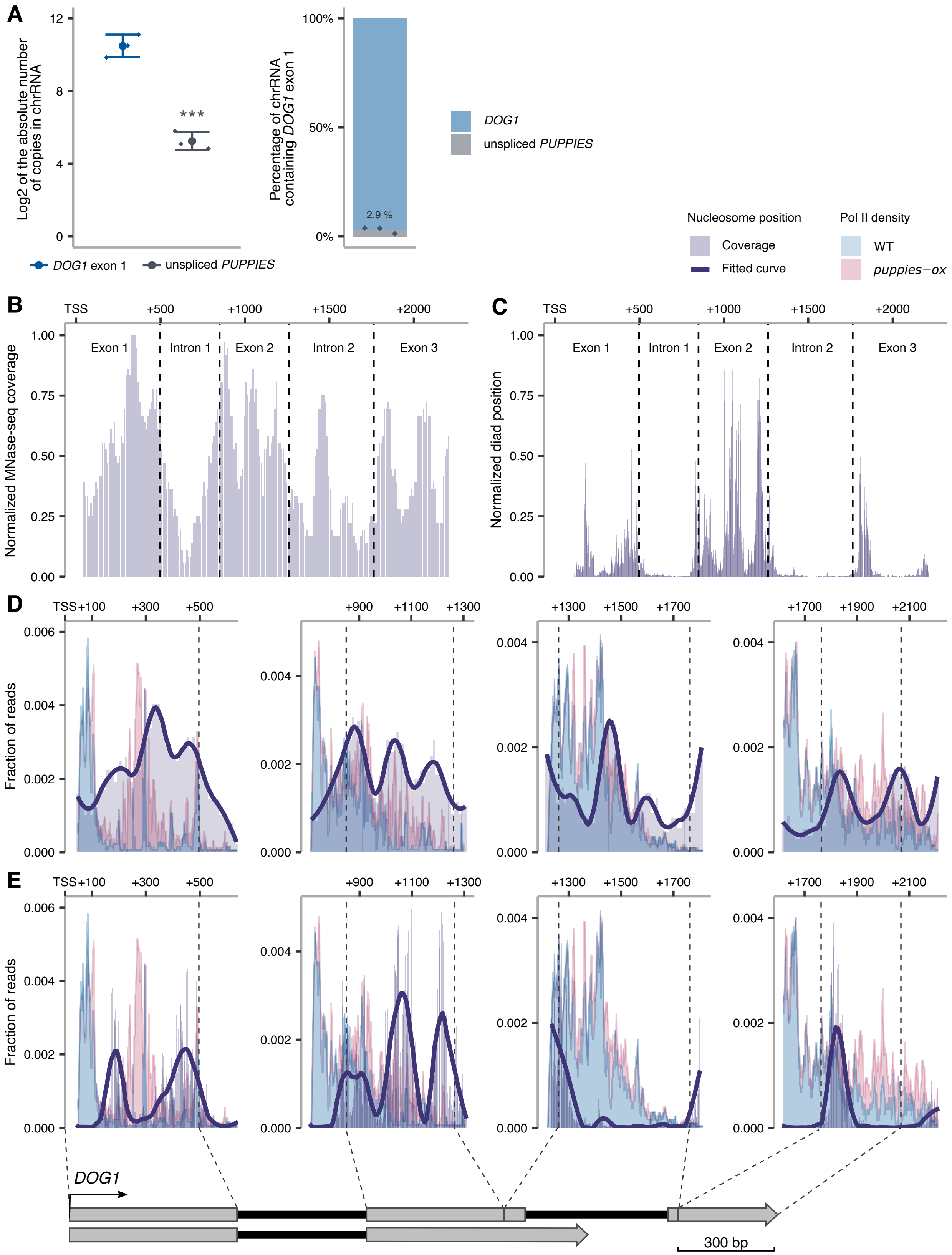

**Appendix Figure S6 - Overlap of nucleosome position with Pol II profile on *DOG1 locus***

A The plot on the left shows the absolute levels (in log2 of the number of copies) of chromatin-attached unspliced *PUPPIES* and transcripts containing *DOG1* exon 1, in WT seeds under salt stress, measured by absolute RT-qPCR quantification. The plot on the right shows the levels of unspliced *PUPPIES* as the percentage of absolute *DOG1* exon 1 copy number. The *DOG1* levels (in light blue) are estimated by subtracting the number of copies of unspliced *PUPPIES* from the total *DOG1* exon 1, displayed in percentage.

B Plot representing read coverage from the MNase-seq dataset GSM4916341 (Data ref: Luo *et al*., 2020b) on the *DOG1 locus*.

C Plot representing nucleosome position prediction *in silico* based on genomic DNA sequence of the *DOG1 locus* (van der Heijden *et al*., 2012). Distance is given in bp from *DOG1* TSS. Vertical dashed lines show the exon boundaries, with text annotation for exons and introns.

D, E Overlap of targeted NET-Seq profiles of Pol II density for WT (blue) and *puppies-ox* (red) with nucleosome position from MNase-seq (Data ref: Luo *et al*., 2020b; D) and from *in silico* prediction (E). The nucleosome position is represented by purple lines and bars. A schematic of the *DOG1 locus* is given at the bottom with dashed lines connecting the exon boundaries with the plots. Dark grey boxes for exons of *lgDOG1* RNA isoform and light grey boxes for exons of *shDOG1* RNA isoform.
