# Supplementary material for: Promoter‐pervasive transcription causes RNA polymerase II pausing to boost DOG1 expression in response to salt
Source: EMBO J. 2023 Jan 27;42(5):e112443. doi: 10.15252/embj.2022112443 (PMC9975946; doi:10.15252/embj.2022112443)
Supplement: Supplementary file 9 [file EMBJ-42-e112443-s008.pdf]

Promoter-pervasive transcription causes RNA polymerase II pausing to boost DOG1 expression in response to salt

Miguel Montez, Maria Majchrowska, Michal Krzyszton, Grzegorz Bokota, Sebastian Sacharowski, Magdalena Wrona, Ruslan Yatusevich, Ferran Massana, Dariusz Plewczynski, and Szymon Swiezewski
DOI: 10.15252/embj.2022112443

Corresponding author(s): Szymon Swiezewski (sswiez@ibb.waw.pl) , Miguel Montez (miguel-coelho@hotmail.com)

Review Timeline:

Submission Date:	23rd Aug 22
Editorial Decision:	22nd Sep 22
Revision Received:	28th Nov 22
Editorial Decision:	28th Dec 22
Revision Received:	2nd Jan 23
Accepted:	9th Jan 23

Editor: Stefanie Boehm

Transaction Report:

(Note: With the exception of the correction of typographical or spelling errors that could be a source of ambiguity, letters and reports are not edited. Depending on transfer agreements, referee reports obtained elsewhere may or may not be included in this compilation. Referee reports are anonymous unless the Referee chooses to sign their reports.)

1st Editorial Decision **22nd Sep 2022**

Thank you for submitting your manuscript reporting salt-responsive upstream lncRNA transcripts for the Arabidopsis DOG1 locus to The EMBO Journal. We have now received three referee reports on your study (copied below). After discussing these comments further, also with the referees, we have decided to invite you to prepare and submit a revised version of the manuscript.

As you will see, all referees acknowledge the interest of the findings for the field, but also raise several critical issues that must be resolved in the next and final version. In particular, as referee #1 argues in detail (also ref#2- point 1; referee #3- point 3), the genetic strategy to differentiate effects resulting from pervasive transcription of PUPPIES to those potentially due to alterations to the DOG1 promoter itself, must be reassessed and additional experimental data added and the conclusions carefully revised. In addition, the smFISH analyses should be expanded and the referees' issues resolved, as well as those related to transcriptional burst size and RNAPII occupancy (ref #1- point 3, 4; ref #2- point 2, (3); ref #3- point 1, 2). Please also carefully consider all other points the referees raise and revise the text and figures as appropriate. In addition, please also remember to include a detailed response to each of the comments when submitting a revised version.

Referee #1:

This ms describes the presence of a series of lncRNAs, called PUPPIES, in the promoter of DOG1, an important regulator of seed dormancy. Here, the author showed that PUPPIES are activated by salt stress and correlate the expression of PUPPIES with DOG1 in a variety of conditions and mutants. The results of the studies rely on the analysis of two mutations in the promoter of DOG1. One T-DNA insertion in the proximal promoter region (puppies-1, around 400 bp from the promoter, and a CRISPR mediated deletion from (-186 to -449) upstream of PUPPIES TSS that is in the distal DOG1 promoter region, about 1.7 kb upstream the DOG1 TSS (puppies-ox).
As expected, the T-DNA insertion (puppies-1, a large T-DNA insertion in the DOG1 promoter common to the transcriptional overlapping of PUPPIES that completely distorted this DNA region) in the proximal promoter strongly decreases PUPPIES and DOG1 expression. This mutant displays a weaker inhibition of seed germination upon salt stress. Whereas the deletion in the distal promoter (1.7 kb from DOG1 and around 200 bp for PUPPIES) induce both PUPPIES and DOG1 expression at high levels confirming that a repressor element acts through this region. Its germination is less sensitive to salt. Then, they made smFISH RNA to observe DOG1 expression in the cytoaplsm of WT and mutants coupled with a series of PolII deposition experiments to analyze transcription of this locus.

Based on these observations the authors conclude : "we obtained PUPPIES knock-down and overexpression mutants which uncover that PUPPIES are positive regulators of DOG1 expression acting both during seed maturation and seed germination under salt stress." However, this genetic strategy does not allow disentangling the effect of modulating pervasive RNA transcription in the promoter of DOG1 (PUPPIES expression) from the effect of directly impairing DOG1 transcription by changing the DNA sequence of the promoter. Indeed, introduction of such mutations in the promoter of DOG1 could simply impair the binding of transcription factor to cis-regulatory element as an alternative explanation of all experiments as they are essentially correlative between DOG1 and PUPPIES.
In addition,

1) the fact that the authors show that the dog1-3 KO T-DNA insertion in the first exon of DOG1 does not impairs PUPPIES expression does not allow to conclude that "PUPPIES transcription is independent of DOG1 ". A better strategy would have been to use CRISPR interference (CRISPRi) to alter PUPPIES expression without changing DOG1 promoter sequence. I recognize that this type of cis-effects are particularly difficult to deal with but at present there is no evidence that PUPPIES has anything to do with the regulation of DOG1 expression under salt stress.
2) What is the evidence that "PUPPIES induce the levels of DOG1 steady state mRNA"? Not the mutants reported. Adding PUPPIES to a plant without them or activating PUPPIES with a dead CRISPR activator or repressor can be used. Even an RNAi approach may serve but not drastic changes in DNA sequence.
3) The smFISH experiments are not clear and arrows should indicate what the authors want to highlight (e.g. there are foci in the dog1-3 mutant in SFig 7 d; the foci do not augment in the puppies-ox, the "nuclear" localized foci must clearly been indicated using stronger resolution)

4) What about the localization of PUPPIES lncRNAs under salt stress and control in comparison to DOG1? At present this smFISH data is mainly correlative to the PolII and qRT-PCR
5) Using promoter construct with or without PUPPIES promoter may better serve to analyse for concluding about this regulation although careful exploration to separate the endogenous PUPPIES from the "transgenic" reporter need to be undertaken

6. PUPPIES are induced at promoter level by salt stress. To conclude that "PUPPIES constitute an important environmental sensing element" is clearly overstated without any further data (e.g. RNA structure changes or modifications). It is just an inducible lncRNA.
7. The concept of "burst size" needs to be further explained and quantified to conclude about particular transcriptional features.

Referee #2:

This is an exciting study that contributes to the understanding of how important lncRNAs are at regulating transcription in cis
There are a few issues that need some attention:
- The perturbations causing the puppies-1 allele and PUPPIES-ox (generated by the T-DNA insertion and CRISPR Deletion) are remarkably close to the DOG1 promoter. Due to the nature of these lines, and the proximity of these lesions to the DOG1 TSS, the authors have not directly shown that PUPPIES transcription itself is regulating DOG1. Analysis of DOG1 expression in additional lines would strengthen this claim and should also be addressed in the discussion, with caveats added to the strong conclusions in sentences in line 168, 176-177 and 187.
- The authors claim brightness of foci corresponds to burst size. Changes to DOG1 smFISH signal intensity could be a consequence of cotranscriptional changes incurred during PUPPIES transcription. Since the probes cover exonic and intronic DOG1, the change to signal intensity could be related to intron turnover or changes to mRNA release from the locus.
- Without single molecule analysis it is not possible to determine if the NETseq peak in exon 1 is from DOG1 promoter proximal pausing or terminating PUPPIES-fus. The authors comment in the discussion: This is because the primer used in targeted NET-seq for DOG1 exon 1 is located before the 3'ss used in PUPPIES-fusion, therefore the Pol II density signal does not include polymerases transcribing this PUPPIES isoform, leaving only a chance for detection of long unspliced PUPPIES overlapping DOG1 sequence. Given the distance from the 3'ss of PUPPIES-fus to the 'Pol2 pause site' is short(~100nt) and without knowing the splicing efficiency of the long intron, PUPPIES-fus is likely to be unspliced. To address the possible contribution of PUPPIES to the pause site the authors should assay POL2 pausing by SMIT using primers specific to the PUPPIES.
Additionally, the NET-seq (SMIT) in Figure 5b & 5c indicate that there is more exon POL2 signal in the PUPPiES-ox. As this signal is primed from the intron, the signal is a property of POL2 initiation, elongation velocity and splicing efficiency. The authors claim this is the result of slower elongation through exons. However, these data could also be interpreted as lower co-transcriptional splicing efficiency (lower coupling) in the PUPPIES-ox. This also feeds back to the smFISH data where the authors detect brighter signal intensity in these lines. To address this, the authors provide steady state RNA levels showing that DOG1 spliced and unspliced levels fit their model. However, it would be good to include nascent RNA splicing levels of DOG1 to rule out post-transcription splicing obscuring nascent RNA processivity.
Minor comment: The text in lines 118-123 may be better in the discussion.

Referee #3:

The manuscript by Montez and colleagues describes a new role of Arabidopsis DOG1 gene in the regulation of salt stress responses. RNAseq data combined with phenotypic analyses exhibited the requirement of DOG1 for seed germination under high NaCl concentrations. DOG1 transcript is induced by NaCl, which correlated with faster and delayed germination phenotypes of the dog1-3 (reduced DOG1 levels) and dog1-5 (DOG1 over-expressor) mutant lines, respectively. A more detailed look at the transcriptional activity over the DOG1 locus under high NaCl conditions revealed the existence of upstream lncRNA transcripts named PUPPIES. At least three different alternatively spliced PUPPIES isoforms could be detected, all induced by NaCl, and required to delay germination by inducing DOG1 expression under salt stress conditions. In contrast to previous examples of transcriptional interference, the authors propose a model whereby PUPPIES pervasive transcription induces expression of downstream co-directional DOG1 gene. PUPPIES overexpression is associated with increased Pol II promoter-proximal pausing and slower transcription through exonic regions, possibly allowing the loading of addition Pol II molecules per round of transcription. This manuscript provides a new example of regulation of plant stress responses by lncRNA transcripts. Moreover, this work presents evidence that promoter upstream non-coding transcripts are able to induce transcription of downstream transcripts. I have some comments to improve this manuscript.

Major comments

1- I appreciate the effort that the authors have made in applying smFISH in Arabidopsis embryos to address the molecular mechanism of PUPPIES, given the limitations of this method in plants. However, the following points should be addressed:

- Although mentioned in Methods, it is not clear to this reviewer whether the experiments presented in Figure 4 and SFig 7 were done under salt stress conditions. Please clarify this in the main text and figure legends. Besides, it would be interesting to see images of DOG1 expression by smFISH with and without NaCl treatment.

- Overall, using probes that detect mRNA may not be the best choice for this manuscript in which the authors claim that the act of PUPPIES transcription has a regulatory effect on Pol II dynamics over the DOG1 locus. For example, Fig. 4b shows the high variability in mRNA quantity per cell, but does not add much information about the proposed transcriptional dynamics model. I am not totally convinced that the mRNA probes are suitable to measure transcriptional bursts. I wonder if it would be possible to repeat these experiments using probes designed against lgDOG1, including both introns, and improving the proportion of intronic/exonic probes.

- The representative images presented for WT in Fig. 4a, c and g are misleading. For instance, WT in Fig. 4g looks very different than those in Fig. 4a and c. When looking at the mutants, puppies-1 shows clearly lower expression than the WT in Fig. 4c but perhaps not than WT in Fig. 4g. Please clarify the differences observed. Adding additional representative images of WT and mutants in supplementary files could help. In addition, please explain what tissues/cell types of the embryo have been imaged.

2- The authors hypothesize that the increased amounts of DOG1 transcripts induced by PUPPIES may be due to loading of a higher number of Pol II molecules per round of transcription, based on smFISH and targeted NET-seq. However, these are not direct measurements of Pol II enrichment over the DOG1 locus. Pol II ChIP experiments in WT vs mutants (puppies-1 and puppies-ox) could help to support the model proposed.

3- The authors propose that the act of transcription from the PUPPIES upstream locus is what activates transcription of the DOG1 downstream locus. However, the analyses described here were carried out in either a T-DNA mutant (puppies-1) or a CRISPR mutant (puppies-ox). The authors cannot exclude the possibility that genetic alterations of DOG1 upstream sequences could be the cause of the phenotype, independent of PUPPIES expression. This possibility should be discussed in the text.

4- Based on analysis of the dog1-3 T-DNA line (Fig. 2a), the conclusion is that "PUPPIES transcription is independent of DOG1 and that upon salt stress PUPPIES act upstream of, and as a positive regulator of DOG1 expression to control seed germination". Is this also true for dog1-5 T-DNA line?

5- The augmented levels of shDOG1 mRNA under 100mM NaCl seem to be equivalent between dog1-5 (Fig. 1c) and puppies-ox plants (Fig. 3e). Yet, the delayed germination phenotype of dog1-5 under 100mM NaCl (Fig. 1b) is markedly different than that observed for puppies-ox (Fig. 3d). Some comments about possible explanations for the differences observed would improve the manuscript.

Minor comments

- Fig. 3f, please choose other color combination to clearly differentiate between dry seed and NaCl data.

Author’s response

We are pleased to submit the revised version of our manuscript (EMBOJ-2022-112443) entitled “Pervasive transcription on *DOG1* promoter causes Pol II pausing to boost its expression in response to salt”.

First of all, we thank the reviewers for their comprehensive list of comments that were very insightful and allowed us to strengthen our claims. We also thank the editor for handling our process with notable speed and professionalism.

We have addressed the reviewer’s comments and suggestions, and we provide additional experimental data (presented in **Figures EV1E**, **EV2**, **EV3**, **EV4K**, **EV5, 5H** and **Appendix Fig S6A**), as well as additional text together with minor changes in the attached .pdf file (all changes marked).

Below, we provide a detailed point-by-point response to the reviewers’ comments.

***IMPORTANT NOTE: we now perform an initial quality control of all revised manuscripts before re-review. Your manuscript will FAIL this control and the handling will be DELAYED if the following APPLIES:

1) A data availability section is missing.
2) Your manuscript contains error bars based on n=2. Please use scatter blots showing the individual datapoints in these cases. The use of statistical tests needs to be justified.

When submitting your revised manuscript, please carefully review the instructions that follow below. Failure to include requested items will delay the evaluation of your revision.***

The Data Availability section was added to the revised version of the manuscript according to the Journal’s publication guidelines.

The data presented in Fig. 3c for *puppies-1* in the initial manuscript was based on data from two independent biological replicates (n = 2). In the new **Fig 3C**, that was replaced by different data from three independent biological replicates (n = 3). The data used for the matching WT control is the same as plotted for WT in **Fig 1B**.

Referee #1:

This ms describes the presence of a series of lncRNAs, called PUPPIES, in the promoter of DOG1, an important regulator of seed dormancy. Here, the author showed that PUPPIES are activated by salt stress and correlate the expression of PUPPIES with DOG1 in a variety of conditions and mutants. The results of the studies rely on the analysis of two mutations in the promoter of DOG1. One T-DNA insertion in the proximal promoter region (puppies-1, around 400 bp from the promoter, and a CRISPR mediated deletion from (-186 to -449) upstream of PUPPIES TSS that is in the distal DOG1 promoter region, about 1.7 kb upstream the DOG1 TSS (puppies-ox).

As expected, the T-DNA insertion (puppies-1, a large T-DNA insertion in the DOG1 promoter common to the transcriptional overlapping of PUPPIES that completely distorted this DNA region) in the proximal promoter strongly decreases PUPPIES and DOG1 expression. This mutant displays a weaker inhibition of seed germination upon salt stress. Whereas the deletion in the distal promoter (1.7 kb from DOG1 and around 200 bp for PUPPIES) induce both PUPPIES and DOG1 expression at high levels confirming that a repressor element acts through this region. Its germination is less sensitive to salt. Then, they made smFISH RNA to observe DOG1 expression in the cytoaplsm of WT and mutants coupled with a series of PolII deposition experiments to analyze transcription of this locus.

Based on these observations the authors conclude : "we obtained PUPPIES knock-down and overexpression mutants which uncover that PUPPIES are positive regulators of DOG1 expression acting both during seed maturation and seed germination under salt stress." However, this genetic strategy does not allow disentangling the effect of modulating pervasive RNA transcription in the promoter of DOG1 (PUPPIES expression) from the effect of directly impairing DOG1 transcription by changing the DNA sequence of the promoter. Indeed, introduction of such mutations in the promoter of DOG1 could simply impair the binding of transcription factor to cis-regulatory element as an alternative explanation of all experiments as they are essentially correlative between DOG1 and PUPPIES.

The reviewer is completely right. Disentangling the function of transcription/transcript from its inherent DNA sequence is unarguably key to understanding lncRNAs. This very same issue was raised by all three reviewers. As Reviewer #1 points out throughout points 1), 2) and 5), one way to address this concern is to repress *PUPPIES* without changing the genomic sequence of the *DOG1 locus* or to provide an additional source of *PUPPIES*. We have addressed both of those suggestions.

We have generated transgenic lines carrying a construct that constitutively expresses a “dead” or inactive Cas9 protein (dCas9) and sgRNAs targeting *PUPPIES* in different locations. This system was used to

target dCas9 to *DOG1* promoter without cleaving it and therefore create a roadblock to polymerases transcribing *PUPPIES* while causing no changes to the genomic sequence (Bikard et al., NAR 2013; Qi et al., Cell 2013; Piatek et al., Plant Biotechnol. J. 2017). The results presented in **Fig EV2** show that dCas9 transgenic plants display faster germination under salt stress. In selected lines, we also show that *PUPPIES* levels are indeed reduced, and consequently the *DOG1* expression is downregulated. These new results are consistent with the data from *puppies-1* and *puppies-ox* mutants and expression patterns during seed maturation and salt stress. We agree that this is an important additional piece of evidence supporting the role of *PUPPIES* transcription/transcripts and not the underlying genomic sequence.

Because of the overlap of *PUPPIES* with *DOG1* promoter and gene body, we reasoned that the use of transgenes would not be the best suited to provide an additional source of *PUPPIES*. Instead, we crossed *puppies-1* mutant that lacks *PUPPIES* and expresses *DOG1* at a reduced level with *dog1-3* mutant which lacks detectable *DOG1* expression but has a normal level of *PUPPIES* expression. The results presented in **Fig EV3A** and **B** show no induction of *DOG1* despite the presence of *PUPPIES* originating from the *dog1-3* allele. We propose that this reflects the *cis*-acting nature of *PUPPIES* and therefore their function over *DOG1* cannot be tested this way. Due to concerns regarding the use of T-DNA mutants in this assay, we further supported our claim by crossing two independent transgenic lines of *DOG1-LUC* reporter line with either WT or *puppies-ox*, and testing whether enhanced expression of *PUPPIES* from the *puppies-ox* allele, leads to induction of the *DOG1* from the *DOG1-LUC* reporter allele. The results presented in **Fig EV3C** and **D** further support that *PUPPIES* are unable to regulate *DOG1* expression in *trans*.

In addition,

1) the fact that the authors show that the dog1-3 KO T-DNA insertion in the first exon of DOG1 does not impairs PUPPIES expression does not allow to conclude that "PUPPIES transcription is independent of DOG1 ". A better strategy would have been to use CRISPR interference (CRISPRi) to alter PUPPIES expression without changing DOG1 promoter sequence. I recognize that this type of cis-effects are particularly difficult to deal with but at present there is no evidence that PUPPIES has anything to do with the regulation of DOG1 expression under salt stress.

In the first part of this point 1), Reviewer #1 points out that the fact that *PUPPIES* are not downregulated in *dog1-3* is not enough to conclude that *PUPPIES* transcription is independent of *DOG1*. In the revised version of this manuscript, we have included additional data which convinced us that *PUPPIES* do not always follow the same expression changes as *DOG1*. It was shown by us before that during seed imbibition under heat stress, *DOG1* is strongly induced (Krzyszton et al., Plant Physiol. 2022). The data presented in **Fig EV1E** shows that in contrast to *DOG1*, *PUPPIES* expression is not induced during this

treatment and remains at very low levels. These results provide a piece of important additional evidence that *PUPPIES* regulation differs from that of *DOG1*, and that *DOG1* expression can be either strongly repressed (*dog1-3*) or induced (secondary dormancy) without changing *PUPPIES* expression.

In the second part of this point 1), Reviewer #1 proposed CRISPR interference (CRISPRi) to alter *PUPPIES* expression without changing *DOG1* promoter sequence, and acknowledges the challenges of these types of experiments. As mentioned in detail in the above response to the reviewer, we used dCas9 to repress *PUPPIES* without changing the genomic sequence. We believe this a good alternative to the approach proposed by the reviewer.

2) What is the evidence that "PUPPIES induce the levels of DOG1 steady state mRNA"? Not the mutants reported. Adding PUPPIES to a plant without them or activating PUPPIES with a dead CRISPR activator or repressor can be used. Even an RNAi approach may serve but not drastic changes in DNA sequence.

In this point, Reviewer #1 notes that the T-DNA and CRISPR-Cas9 deletion mutants are not sufficient to claim that *DOG1* expression is regulated by *PUPPIES*. The additional data from dCas9 experiments (described in detail above and in the revised version of the manuscript) support the view that *PUPPIES* do regulate *DOG1* expression. That, together with changes observed in the T-DNA and CRISPR-Cas9 deletion mutants builds up our confidence that *PUPPIES* induce the levels of *DOG1* steady-state mRNA.

3) The smFISH experiments are not clear and arrows should indicate what the authors want to highlight (e.g. there are foci in the dog1-3 mutant in SFig 7 d; the foci do not augment in the puppies-ox, the "nuclear" localized foci must clearly been indicated using stronger resolution)

In the revised version of the manuscript, we have provided additional representative images with arrowheads indicating relevant information. We have also replaced one image in the outdated Fig. 4g with a new image with a higher resolution that better represents the data in the new **Fig 4G**.

The reviewer points out that *foci* are visible in the *dog1-3* image in the outdated SFig. 7d now **Fig EV4D**. Dots are indeed observed in that image, yet most of them are background signal. It is hard to present this kind of background without reference dots that truly correspond to *DOG1* transcripts. To make it clearer, we have added an additional image in **Fig EV4E** where one dot is visible in *dog1-3* (which we believe corresponds to an active *DOG1* transcription site). This dot is used as a reference to adjust the min and max intensity brightness in the image.

The reviewer mentions that “the foci do not augment in the puppies-ox”. We do agree that the image provided for *puppies-ox* presented in the outdated Fig. 4g was not the best-suited image. We have

replaced it with a new image in **Fig 4G**. The new image has better resolution, presents cells more similar to WT cells, a cleaner cell layer (without additional cells and nuclei from the stacks above and below which get projected in the 2D image), and arrowheads pointing to the *DOG1* transcription sites. We have also provided an extra image of WT and *puppies-ox* in **Fig EV4J**.

4) What about the localization of PUPPIES lncRNAs under salt stress and control in comparison to DOG1? At present this smFISH data is mainly correlative to the PolII and qRT- PCR

The reviewer raises a very interesting question. To visualize the localization of *PUPPIES* lncRNAs we would have to perform smFISH with probes specific to these transcripts. We did not perform such an experiment due to major issues regarding the probes. *PUPPIES* are very diverse in sequence due to alternative splicing and termination. The 60 bp-long *PUPPIES* exon 1 shared by all isoforms, is far too short to design the minimal number of probes required to obtain a specific signal (which is around 35 probes of 18-21 nt each spaced by at least 1 nt). Similarly, the length of the different alternative *PUPPIES* exons is still not enough to obtain a specific signal. Alternatively, a set of probes covering the entire *DOG1* promoter-transcribed region (which is mostly *PUPPIES* intronic regions) could be used, but would also not be suited as intronic probes would target the nascent transcripts only, providing no clues into the localization of *PUPPIES* spliced lncRNAs.

5) Using promoter construct with or without PUPPIES promoter may better serve to analyse for concluding about this regulation although careful exploration to separate the endogenous PUPPIES from the "transgenic" reporter need to be undertaken

Deletion constructs are a classical way to address the function of lncRNAs. Yet, in the particular case of *PUPPIES*, we believe that the use of transgenes would not be the best-suited strategy since we show (in the revised version of the manuscript) that *PUPPIES* do not regulate *DOG1* in *trans*. *cis* effects of lncRNA can also be studied using transgenes but in the case of *PUPPIES*, its overlap with *DOG1* promoter would by necessity imply altering the DNA sequence of *DOG1* promoter. We reasoned from the start that it would be a better approach to generate CRISPR-Cas9 deletions in the endogenous *DOG1* sequence instead. To support our conclusion about *PUPPIES* acting in *cis* to activate *DOG1*, we provide additional data using the dCas9 system as described above and in the revised version of the manuscript.

6. PUPPIES are induced at promoter level by salt stress. To conclude that "PUPPIES constitute an important environmental sensing element" is clearly overstated without any further data (e.g. RNA structure changes or modifications). It is just an inducible lncRNA.

We do agree with Reviewer #1 that it is an overstated conclusion that "*PUPPIES* constitute an important environmental sensing element". We have therefore removed this statement. In the revised version of the manuscript, we provide data showing that in contrast to salt stress in response to heat stress, *PUPPIES* are not induced while *DOG1* is (**Fig EV1E**). This suggests some level of specificity of the *PUPPIES*-mediated regulation of *DOG1* expression. In the revised manuscript, we have made changes to the initial claims and suggest only partial independence of *PUPPIES* from *DOG1* regulation (lines 180-181 and 387-388).

7. The concept of "burst size" needs to be further explained and quantified to conclude about particular transcriptional features.

We agree with the reviewer that the concept of "burst size" was not clearly presented. In the revised version we have provided additional text in the Results section (lines 263-265): "The release of Pol II from promoters often happens in bursts with the transcription by several Pol II enzymes followed by a period of absence of initiation events, a phenomenon called transcriptional bursting. The number of Pol II per burst of transcription is defined as transcriptional burst size (Cai et al., 2008; Raj et al., 2006; Suter et al., 2011; Zenklusen et al., 2008)". Additional text was added to the Discussion section (lines 392-404).

We have also provided additional control experiments as suggested by Reviewer #1 and Reviewer #2. In essence, the increased intensity of *foci* corresponding to transcription sites in the *puppies-ox* mutant could be explained by increased intron retention or, alternatively, by retention of full-length transcripts at the chromatin. We have analysed splicing in chromatin-attached RNA and found that the *puppies-ox* mutant has lower intron retention (**Fig 5H**) excluding the possibility that intron retention explains the brighter signal. Also, our analysis of the ratio of 3'/5' ends of *DOG1* chromatin attached RNA suggests that the levels of full-length transcripts retained at chromatin are not increased in *puppies-ox* (**Fig EV4K**). Furthermore, the smFISH analyses, including analysis of transcriptional burst size were expanded to the WT response to NaCl (as suggested by Reviewer #3). The data is presented in **Fig EV5**.

Referee #2:

This is an exciting study that contributes to the understanding of how important lncRNAs are at regulating transcription in cis

There are a few issues that need some attention:

- The perturbations causing the puppies-1 allele and PUPPIES-ox (generated by the T-DNA insertion and CRISPR Deletion) are remarkably close to the DOG1 promoter. Due to the nature of these lines, and the proximity of these lesions to the DOG1 TSS, the authors have not directly shown that PUPPIES transcription itself is regulating DOG1. Analysis of DOG1 expression in additional lines would strengthen this claim and should also be addressed in the discussion, with caveats added to the strong conclusions in sentences in line 168, 176- 177 and 187.

We thank Reviewer #2 for the comments and positive feedback. We agree that the perturbations to *DOG1* genomic sequence in *PUPPIES* T-DNA and CRISPR-Cas9 deletion mutants are of major concern. To address this point, in the revised version of the manuscript we used a catalytically inactive dCas9 system to repress *PUPPIES* without changing their underlying genomic sequence. The results presented in **Fig EV2** show that dCas9 transgenic plants display faster germination under salt stress, and consistent with such phenotype, selected lines show downregulation of *PUPPIES* and *DOG1*. While we cannot exclude the possibility that dCas9 binding interferes with for example the binding of *DOG1* activators, the fact that 3 different sets of sgRNAs gave the same result suggests that *PUPPIES* do activate *DOG1* expression. The sentences in lines 176-177 and 187 from the initial version have been modified. We have also addressed this in the Discussion section of the revised manuscript.

- The authors claim brightness of foci corresponds to burst size. Changes to DOG1 smFISH signal intensity could be a consequence of cotranscriptional changes incurred during PUPPIES transcription. Since the probes cover exonic and intronic DOG1, the change to signal intensity could be related to intron turnover or changes to mRNA release from the locus.

The reviewer is right and raises an important point. The smFISH signal intensity of *foci* corresponding to *DOG1* transcription sites is not only dependent on the number of Pol II/nascent RNAs but also on the length of such chrRNAs. Cotranscriptional splicing and mRNA release from the *locus* are possibly the two major contributors to changes in the length of chrRNA and therefore important to be addressed when analysing smFISH intensities.

In the revised version of the manuscript, we have provided new data addressing those possibilities. One can expect that lower cotranscriptional intron splicing efficiency would result in higher smFISH signal

intensity due to more probes being able to hybridise with the nascent RNA. In the initial version of the manuscript, we showed increased splicing efficiency in *puppies-ox* (Fig. 5f), when analysed in total RNA. However, that does not necessarily reflect cotranscriptional splicing and therefore, we repeated this analysis on chrRNA. The data presented in **Fig 5H** shows that in chrRNA, intron 1 splicing is more and less efficiently spliced in *puppies-ox* and *puppies-1*, respectively. This suggests that intron retention does not explain the changes in brightness of foci corresponding to transcription sites in *PUPPIES* mutants.

We also reasoned that the changes in smFISH signal in *PUPPIES* mutants are not caused by changes in mRNA release from the *locus* as we do not observe a strong accumulation of targeted NET-seq reads at the 3' end of *DOG1* in *puppies-ox* (**Fig 5C** and **E**). To corroborate this conclusion, we isolated chrRNA and calculated the ratio of 3' to 5' ends for *DOG1*, so that for example a higher 3' to 5' ends ratio would reflect a higher abundancy of longer transcripts retained at the chromatin, which could explain the stronger smFISH signal observed in *puppies-ox*. In contrast, **Fig EV4K** shows that *puppies-ox* has no statistically significant differences in 3'/5' ends ratio compared to WT, and in fact slightly lower ratio on average. This could be explained by the massive promoter-proximal pausing which even though is balanced by slower transcription throughout exons 1 and 2, still skews the ratio towards 5' ends, compared to WT. No significant differences in 3'/5' ends ratio are observed for *puppies-1* as well. We would like to thank Reviewer #2 for the suggestions as we are confident that the manuscript benefits from having these additional controls.

- Without single molecule analysis it is not possible to determine if the NETseq peak in exon 1 is from DOG1 promoter proximal pausing or terminating PUPPIES-fus. The authors comment in the discussion: This is because the primer used in targeted NET-seq for DOG1 exon 1 is located before the 3'ss used in PUPPIES-fusion, therefore the Pol II density signal does not include polymerases transcribing this PUPPIES isoform, leaving only a chance for detection of long unspliced PUPPIES overlapping DOG1 sequence. Given the distance from the 3'ss of PUPPIES-fus to the 'Pol2 pause site' is short(~100nt) and without knowing the splicing efficiency of the long intron, PUPPIES-fus is likely to be unspliced. To address the possible contribution of PUPPIES to the pause site the authors should assay POL2 pausing by SMIT using primers specific to the PUPPIES.

This is a very interesting point. As we discussed in the initial version of the manuscript, we used a primer on *DOG1* exon 1 that is located before the 3' splice site of *PUPPIES-fusion*, therefore preventing obtaining NET-seq signal from this spliced transcript. However, as pointed out by the reviewer, the NET-seq signal may come from unspliced *PUPPIES* that transcribe into *DOG1*.

The reviewer proposes to perform SMIT (targeted NET-seq) assay using primers specific to *PUPPIES*. We believe that while feasible this experiment will not clarify the putative contribution of *PUPPIES*-

transcribing polymerases to the massive pausing that we observe on *DOG1* exon1 using a primer located at beginning of *DOG1* exon 1. This is because the targeted NET-seq is relative due to PCR library preparation with gene-specific primers, and the sequencing signal from different primers cannot be directly compared. Instead, to clarify this, we have performed absolute RT-qPCR quantification of chrRNA to measure the number of unspliced *PUPPIES* that are transcribed into *DOG1* and could potentially contribute to changes in the targeted NET-seq signal. Using primers close to *DOG1* TSS the ct values were too late laying outside of the linearity of the standard curves. Nonetheless, we were able to measure total unspliced *PUPPIES* (with primers for *PUPPIES* exon 1-intron 1 junction), which gives us an overestimation of the unspliced *PUPPIES* that read into *DOG1*. The results show that unspliced *PUPPIES* constitute only 2.9 % of total chrRNA transcripts containing *DOG1* exon 1, in WT (**Appendix Fig S6A**). Therefore, we believe that the unspliced *PUPPIES* that read into *DOG1* are very unlikely to explain the strong increase in Pol II signal observed in the NET-seq.

Additionally, the NET-seq (SMIT) in Figure 5b & 5c indicate that there is more exon POL2 signal in the PUPPiES-ox. As this signal is primed from the intron, the signal is a property of POL2 initiation, elongation velocity and splicing efficiency. The authors claim this is the result of slower elongation through exons. However, these data could also be interpreted as lower co-transcriptional splicing efficiency (lower coupling) in the PUPPIES-ox. This also feeds back to the smFISH data where the authors detect brighter signal intensity in these lines. To address this, the authors provide steady state RNA levels showing that DOG1 spliced and unspliced levels fit their model. However, it would be good to include nascent RNA splicing levels of DOG1 to rule out post-transcription splicing obscuring nascent RNA processivity.

Reviewer #2 points out that the high NET-seq signal over *DOG1* exons 2 and 3 in *puppies-ox* could reflect lower cotranscriptional splicing efficiency. As requested by the reviewer we have performed the splicing analysis on chrRNA and discussed the results in detail in Reviewer #2 point 2. In brief, the data presented in **Fig 5H** shows that intron 1 splicing is more efficient in *puppies-ox*, and therefore cannot explain the higher NET-seq signal.

Minor comment: The text in lines 118-123 may be better in the discussion.

We agree with the reviewer and we moved the text in lines 118-123 to the Discussion section.

Referee #3:

The manuscript by Montez and colleagues describes a new role of Arabidopsis DOG1 gene in the regulation of salt stress responses. RNAseq data combined with phenotypic analyses exhibited the requirement of DOG1 for seed germination under high NaCl concentrations. DOG1 transcript is induced by NaCl, which correlated with faster and delayed germination phenotypes of the dog1-3 (reduced DOG1 levels) and dog1-5 (DOG1 over-expressor) mutant lines, respectively. A more detailed look at the transcriptional activity over the DOG1 locus under high NaCl conditions revealed the existence of upstream lncRNA transcripts named PUPPIES. At least three different alternatively spliced PUPPIES isoforms could be detected, all induced by NaCl, and required to delay germination by inducing DOG1 expression under salt stress conditions. In contrast to previous examples of transcriptional interference, the authors propose a model whereby PUPPIES pervasive transcription induces expression of downstream co-directional DOG1 gene. PUPPIES overexpression is associated with increased Pol II promoter-proximal pausing and slower transcription through exonic regions, possibly allowing the loading of addition Pol II molecules per round of transcription. This manuscript provides a new example of regulation of plant stress responses by lncRNA transcripts. Moreover, this work presents evidence that promoter upstream non-coding transcripts are able to induce transcription of downstream transcripts. I have some comments to improve this manuscript.

We appreciate the reviewer's positive assessment of our work and thank for the constructive comments below.

Major comments

1- I appreciate the effort that the authors have made in applying smFISH in Arabidopsis embryos to address the molecular mechanism of PUPPIES, given the limitations of this method in plants. However, the following points should be addressed:

- Although mentioned in Methods, it is not clear to this reviewer whether the experiments presented in Figure 4 and SFig 7 were done under salt stress conditions. Please clarify this in the main text and figure legends. Besides, it would be interesting to see images of DOG1 expression by smFISH with and without NaCl treatment.

All the smFISH experiments in the initial version of the manuscript were performed using seeds under salt stress, however, as the reviewer pointed out this information was only in the Methods section and is now added to the revised version in the text and figure legends.

Additionally, as suggested by the reviewer we have extended our smFISH experiments and analyses to the salt stress response in WT. The data presented in **Fig EV5** shows two representative smFISH images of WT with and without NaCl, and the analyses of *DOG1* mRNA per cell, frequency of *DOG1* active transcription and *DOG1* burst size. The results are consistent with our previous data and show that salt stress induces the frequency of active *DOG1* transcription with a higher number of Pol II transcribing per burst, concomitant with observed increase in cytoplasmic mRNA levels.

- Overall, using probes that detect mRNA may not be the best choice for this manuscript in which the authors claim that the act of PUPPIES transcription has a regulatory effect on Pol II dynamics over the DOG1 locus. For example, Fig. 4b shows the high variability in mRNA quantity per cell, but does not add much information about the proposed transcriptional dynamics model. I am not totally convinced that the mRNA probes are suitable to measure transcriptional bursts. I wonder if it would be possible to repeat these experiments using probes designed against lgDOG1, including both introns, and improving the proportion of intronic/exonic probes.

Reviewer #3 is right, that the use of intronic probes would be better suited for the quantification of transcriptional bursting. However, with the current smFISH method, that would not be possible as a set of intronic probes is too small to obtain a specific signal, due to the length and A/T richness of *DOG1* introns.

As suggested by the reviewer, we did try to improve the proportion of intronic/exonic probes and have therefore included in the original design probes that cover *DOG1* intron 1 and part of intron 2 (which is exon 2 of *shDOG1*). The reviewer suggests designing probes that would target the whole intron 2. Unfortunately, this would give us only around 240 bp of additional sequence to use. Also, based on our published absolute quantification of *short* and *long DOG1* (Fedak et al., 2016), Pol II ChIP data (Dolata et al., 2015), and chrRNA analysis (this manuscript), we believe that *lgDOG1* is expressed at a much lower level than the *shDOG1*, suggesting that adding probes to the complete intron 2 would not benefit our imaging.

- The representative images presented for WT in Fig. 4a, c and g are misleading. For instance, WT in Fig. 4g looks very different than those in Fig. 4a and c. When looking at the mutants, puppies-1 shows clearly lower expression than the WT in Fig. 4c but perhaps not than WT in Fig. 4g. Please clarify the differences observed. Adding additional representative images of WT and mutants in supplementary files could help. In addition, please explain what tissues/cell types of the embryo have been imaged.

In the revised version of the manuscript, we have provided additional representative images for all experiments. We hope this will clarify this point. However, we would also like to emphasize that all the experiments were performed using internal controls that were grown and analysed side by side. While there

may be some differences between the experiments, we believe that the comparison to the matched control is the best way to avoid artefacts related to plants growing conditions.

To reduce the influence of cell type-specific expression on our results, we have limited our analyses to the cells from the root meristematic zone of the embryo. With the current smFISH method, the embryos are squashed and we lose the position of the cells. However, some of the tissue architecture is preserved and some cell types can be recognized. We do observe *DOG1* expression in cells from the apical meristem, vasculature and root meristematic zone. Yet, cells from the apical meristem are very small and compact, making it very hard to obtain a nearly single-cell layer, and therefore very challenging to properly perform segmentation of the cells in 3D. Cells from the vasculature are very elongated but narrow, causing the same problem with cell segmentation. The cells from the meristematic zone of the root, however, due to their size, shape and spatial architecture can be easily recognised and segmented, and thus were chosen. This information was added to the Methods section of the revised manuscript.

2- The authors hypothesize that the increased amounts of DOG1 transcripts induced by PUPPIES may be due to loading of a higher number of Pol II molecules per round of transcription, based on smFISH and targeted NET-seq. However, these are not direct measurements of Pol II enrichment over the DOG1 locus. Pol II ChIP experiments in WT vs mutants (puppies-1 and puppies-ox) could help to support the model proposed.

We agree with the reviewer that smFISH and NET-seq are all indirect methods to measure Pol II molecules loaded per round of transcription. However, Pol II ChIP would not help to address the number of Pol II molecules per round of transcription. It would only support the view that *PUPPIES* regulate *DOG1* transcription but we believe the smFISH, NET-seq, chromatin attached RNA analysis as well as the *cis* but not *trans* nature of *PUPPIES* activity all point in this direction.

3- The authors propose that the act of transcription from the PUPPIES upstream locus is what activates transcription of the DOG1 downstream locus. However, the analyses described here were carried out in either a T-DNA mutant (puppies-1) or a CRISPR mutant (puppies-ox). The authors cannot exclude the possibility that genetic alterations of DOG1 upstream sequences could be the cause of the phenotype, independent of PUPPIES expression. This possibility should be discussed in the text.

We agree with the reviewer that the genetic alterations on the *DOG1 locus* in *PUPPIES* mutants could be causing the observed changes in *DOG1* expression, as mentioned by Reviewer #1 and Reviewer #2 as well. We provide a detailed response to this issue in the response to Reviewer #1. Briefly, in the revised manuscript we used a dCas9 system to repress *PUPPIES* without changing their genomic sequence. The results presented in **Fig EV2** show that dCas9 transgenic plants display faster germination under salt stress,

and consistent with such phenotype, a downregulation of *PUPPIES* and *DOG1* supporting the function of *PUPPIES* transcripts/transcription independent of alterations on its underlying sequence. We believe this is an important additional piece of evidence supporting the function of *PUPPIES* that strengthens the confidence in the final conclusion that *PUPPIES* positively regulate *DOG1* expression.

4- Based on analysis of the dog1-3 T-DNA line (Fig. 2a), the conclusion is that "PUPPIES transcription is independent of DOG1 and that upon salt stress PUPPIES act upstream of, and as a positive regulator of DOG1 expression to control seed germination". Is this also true for dog1-5 T-DNA line?

To answer the reviewer's question, we tested the expression of *PUPPIES* in *dog1-5* (a mutant with high *shDOG1* and low or absent *lgDOG1* and antisense *asDOG1* in developing seeds; Fedak et al., 2016). Below we include the data that shows that *PUPPIES* expression, as *DOG1*, is higher in *dog1-5* than in WT seeds imbibed under salt stress.

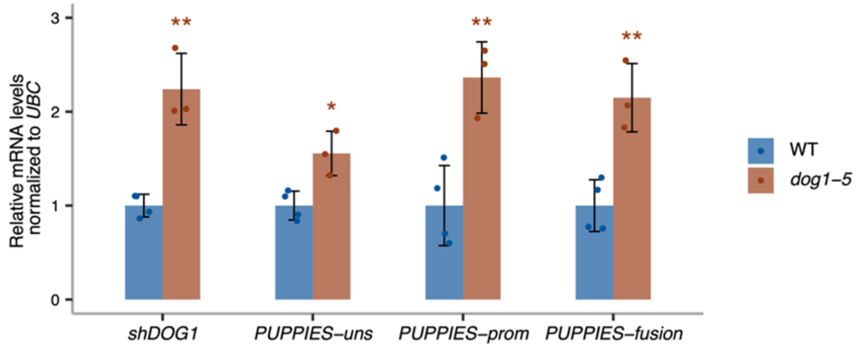

This data together with the positive correlation between *DOG1* and *PUPPIES* expression in response to salt treatment (**Fig EV1A** and **Appendix Fig S1C**) and during seed maturation (**Appendix Fig S5A**) suggests that under some conditions *PUPPIES* are possibly regulated by some of the *DOG1* regulators and/or by *DOG1* itself. In the revised manuscript, we show that *DOG1* expression, but not *PUPPIES*, is upregulated in response to heat stress, triggering secondary dormancy establishment in imbibed seeds (**Fig EV1E**). This, together with the data from *dog1-3* showing no change in *PUPPIES* expression, suggests some level of *PUPPIES* independence and specificity in their regulation. We currently do not fully understand how *PUPPIES* are regulated by *DOG1* or its regulators, however, a good candidate to play a role in this process is the *asDOG1* which is strongly affected in *dog1-5* mutant seeds (Fedak et al., PNAS 2016). The interplay between *PUPPIES*, *DOG1* and *asDOG1* is very interesting and may be addressed by us in the future. In line with the reviewer's suggestion, in the revised manuscript, we have made changes to the initial claims and suggest only partial independence of *PUPPIES* from *DOG1* regulation (lines 180-181 and 387-388).

5- The augmented levels of shDOG1 mRNA under 100mM NaCl seem to be equivalent between dog1-5 (Fig. 1c) and puppies-ox plants (Fig. 3e). Yet, the delayed germination phenotype of dog1-5 under 100mM NaCl (Fig. 1b) is markedly different than that observed for puppies-ox (Fig. 3d). Some comments about possible explanations for the differences observed would improve the manuscript.

Reviewer #3 correctly points out that while *dog1-5* and *puppies-ox* show similar *DOG1* upregulation in seeds imbibed under salt stress, the phenotype of *dog1-5* is much stronger. While we have no definite answer to explain this observation, we believe it may be linked to the fact that during seed maturation *dog1-5* has a much stronger effect on *DOG1* expression than *puppies-ox*. Preliminary results suggest that in contrast to seed germination in salt, during seed maturation *puppies-ox* has up to 3 times higher *DOG1* expression while *dog1-5* (Fedak et al., PNAS 2016) has 5-20 times higher *DOG1* expression, during the initial stages of seed maturation. DOG1 protein was shown to be accumulated during seed maturation and stored stably post-harvest and during imbibition (Nakabayashi et al., Plant Cell 2012). Given the high stability of the DOG1 protein and its posttranslational modifications, we predict that part of the *dog1-5* phenotype could be dependent on the DOG1 protein accumulated during development. Alternatively, the stronger phenotype of *dog1-5* could indicate a suppressive function of the *long DOG1* transcript on for example *shDOG1* translation.

Minor comments

- Fig. 3f, please choose other color combination to clearly differentiate between dry seed and NaCl data.

The colours on the plot from Fig. 3f were changed to allow easier interpretation of the data (**Fig 3F**).

1st Revision - Editorial Decision **28th Dec 2022**

Thank you for submitting your revised manuscript. We have now received comments from all referees (please see below), and I am happy to say they now support publication. Referee #1 notes a potential concern regarding the dCas9 approach, please consider responding to this in a second brief point-by-point response and/or adding to the discussion in the manuscript. In addition, I would ask you to please resolve a number of editorial issues that are listed in detail below. Please use the document that the data editors have added their comments to for any changes. If you have any further questions regarding the specific points listed below or the final manuscript version, please contact me.

Referee #1:

This revised ms has added several experiments that allowed them to conclude that the PUPPIES lncRNAs do not act in trans. The authors agreed with my previous comments that the CRIPSR and T-DNA insertions do not allow to conclude about a relation of causality between PUPPIES transcription and activation of DOG. Indeed, cis-acting lncRNAs are difficult to address in a conclusive manner and multiple approaches are required. Now they have also added conditions where both genes are differentially regulated further supporting that PUPPIES and DOG1 expression maybe unconnected (at least in these two conditions?). They have also now added a dCAS9 approach targeted to the region of PUPPIES RNA and conclude that this experiment "prevent" PUPPIES expression and hence down-regulate DOG1. However, I think they cannot discard that the presence of the dCAS9 protein in the promoter region of DOG1 may also prevent the recruitment of a TF or other factor required for DOG1 gene expression. They have also significantly improved the presentation of the smFISH data and now is clearer the purpose and conclusions of these experiments. The ms have significantly improved and displays a large number of experiments to support their model. All my previous criticisms have been addressed. The story is globally very nice and propose a new mechanism through which pervasive transcription may regulate downstream gene expression.

Referee #2:

This is a nice piece of work detailing how non-coding transcription can influence an important trait. The authors have done an admirable job of addressing the concerns of all the reviewers. I have no additional requests.

Referee #3:

The authors have addressed all my comments. I have no further comments on this manuscript.

All editorial and formatting issues were resolved by the authors.

2nd Revision - Editorial Decision 9th Jan 2023

Thank you again for submitting the final revised version of your manuscript and addressing the remaining points. I am happy to inform you that we have formally accepted your study for publication in The EMBO Journal.

EMBO Press Author Checklist

Corresponding Author Name: Miguel Montez & Szymon Swiezewski	
Journal Submitted to: The EMBO Journal	
Manuscript Number: EMBOJ-2022-112443	

USEFUL LINKS FOR COMPLETING THIS FORM
The EMBO Journal - Author Guidelines
EMBO Reports - Author Guidelines
Molecular Systems Biology - Author Guidelines
EMBO Molecular Medicine - Author Guidelines

Reporting Checklist for Life Science Articles (updated January
This checklist is adapted from Materials Design Analysis Reporting (MDAR) Checklist for Authors. MDAR establishes a minimum set of requirements in transparent reporting in the life sciences (see Statement of Task: 10.31222/osf.io/9sm4x). Please follow the journal's guidelines in preparing your
Please note that a copy of this checklist will be published alongside your article.

Abridged guidelines for figures
1. Data
The data shown in figures should satisfy the following conditions:
- the data were obtained and processed according to the field's best practice and are presented to reflect the results of the experiments in an accurate and unbiased manner.
- ideally, figure panels should include only measurements that are directly comparable to each other and obtained with the same assay.
- plots include clearly labeled error bars for independent experiments and sample sizes. Unless justified, error bars should not be shown for technical
- if n<5, the individual data points from each experiment should be plotted. Any statistical test employed should be justified.
- Source Data should be included to report the data underlying figures according to the guidelines set out in the authorship guidelines on Data

2. Captions
Each figure caption should contain the following information, for each panel where they are relevant:
- a specification of the experimental system investigated (eg cell line, species name).
- the assay(s) and method(s) used to carry out the reported observations and measurements.
- an explicit mention of the biological and chemical entity(ies) that are being measured.
- an explicit mention of the biological and chemical entity(ies) that are altered/varied/perturbed in a controlled manner.
- the exact sample size (n) for each experimental group/condition, given as a number, not a range;
- a description of the sample collection allowing the reader to understand whether the samples represent technical or biological replicates (including how many animals, litters, cultures, etc.).
- a statement of how many times the experiment shown was independently replicated in the laboratory.
- definitions of statistical methods and measures:
  - common tests, such as t-test (please specify whether paired vs. unpaired), simple χ2 tests, Wilcoxon and Mann-Whitney tests, can be unambiguously identified by name only, but more complex techniques should be described in the methods section;
  - are tests one-sided or two-sided?
  - are there adjustments for multiple comparisons?
  - exact statistical test results, e.g., P values = x but not P values < x;
  - definition of 'center values' as median or average;
  - definition of error bars as s.d. or s.e.m.

Please complete ALL of the questions below.
Select "Not Applicable" only when the requested information is not relevant for your study.

Materials

Newly Created Materials	Information included in the manuscript?	In which section is the information available? (Reagents and Tools Table, Materials and Methods, Figures, Data Availability Section)
New materials and reagents need to be available; do any restrictions apply?	Not Applicable	

Antibodies	Information included in the manuscript?	In which section is the information available? (Reagents and Tools Table, Materials and Methods, Figures, Data Availability Section)
For **antibodies** provide the following information:<br>- Commercial antibodies: RRID (if possible) or supplier name, catalogue number and or/clone number<br>- Non-commercial: RRID or citation	Not Applicable	

DNA and RNA sequences	Information included in the manuscript?	In which section is the information available? (Reagents and Tools Table, Materials and Methods, Figures, Data Availability Section)
**Short novel DNA or RNA including primers, probes**: provide the sequences.	Yes	Data Availability Section

Cell materials	Information included in the manuscript?	In which section is the information available? (Reagents and Tools Table, Materials and Methods, Figures, Data Availability Section)
**Cell lines:** Provide species information, strain. Provide accession number in repository **OR** supplier name, catalog number, clone number, and/**OR** RRID.	Not Applicable	
**Primary cultures:** Provide species, strain, sex of origin, genetic modification status.	Not Applicable	
Report if the cell lines were recently **authenticated** (e.g., by STR profiling) and tested for mycoplasma contamination.	Not Applicable	

Experimental animals	Information included in the manuscript?	In which section is the information available? (Reagents and Tools Table, Materials and Methods, Figures, Data Availability Section)
**Laboratory animals or Model organisms:** Provide species, strain, sex, age, genetic modification status. Provide accession number in repository **OR** supplier name, catalog number, clone number, **OR** RRID.	Not Applicable	
**Animal observed in or captured from the field:** Provide species, sex, and age where possible.	Not Applicable	
Please detail **housing and husbandry conditions**.	Not Applicable	

Plants and microbes	Information included in the manuscript?	In which section is the information available? (Reagents and Tools Table, Materials and Methods, Figures, Data Availability Section)
**Plants:** provide species and strain, ecotype and cultivar where relevant, unique accession number if available, and source (including location for collected wild specimens).	Yes	Materials and Methods
**Microbes:** provide species and strain, unique accession number if available, and source.	Not Applicable	

Human research participants	Information included in the manuscript?	In which section is the information available? (Reagents and Tools Table, Materials and Methods, Figures, Data Availability Section)
If collected and within the bounds of privacy constraints report on age, sex and gender or ethnicity for all study participants.	Not Applicable	

Core facilities	Information included in the manuscript?	In which section is the information available? (Reagents and Tools Table, Materials and Methods, Figures, Data Availability Section)
If your work benefited from core facilities, was their service mentioned in the acknowledgments section?	Yes	Materials and Methods

Design

Study protocol	Information included in the manuscript?	In which section is the information available? (Reagents and Tools Table, Materials and Methods, Figures, Data Availability Section)
If study protocol has been **pre-registered, provide DOI in the manuscript**. For clinical trials, provide the trial registration number **OR** cite DOI.	Not Applicable	
Report the **clinical trial registration number** (at ClinicalTrials.gov or equivalent), where applicable.	Not Applicable	

Laboratory protocol	Information included in the manuscript?	In which section is the information available? (Reagents and Tools Table, Materials and Methods, Figures, Data Availability Section)
Provide DOI OR other citation details if **external detailed step-by-step protocols** are available.	Not Applicable	

Experimental study design and statistics	Information included in the manuscript?	In which section is the information available? (Reagents and Tools Table, Materials and Methods, Figures, Data Availability Section)
Include a statement about **sample size** estimate even if no statistical methods were used.	Not Applicable	
Were any steps taken to minimize the effects of subjective bias when allocating animals/samples to treatment (e.g. **randomization procedure**)? If yes, have they been described?	Not Applicable	
Include a statement about **blinding** even if no blinding was done.	Not Applicable	
Describe **inclusion/exclusion criteria** if samples or animals were excluded from the analysis. Were the criteria pre-established?<br><br>If sample or data points were omitted from analysis, report if this was due to attrition or intentional exclusion and provide justification.	Not Applicable	
For every figure, are **statistical tests** justified as appropriate? Do the data meet the assumptions of the tests (e.g., normal distribution)? Describe any methods used to assess it. Is there an estimate of variation within each group of data? Is the variance similar between the groups that are being statistically compared?	Yes	Figure legends, Materials and Methods

Sample definition and in-laboratory replication	Information included in the manuscript?	In which section is the information available? (Reagents and Tools Table, Materials and Methods, Figures, Data Availability Section)
In the figure legends: state number of times the experiment was **replicated** in laboratory.	Not Applicable	
In the figure legends: define whether data describe **technical or biological replicates**.	Yes	Figure legends

Ethics

Ethics	Information included in the manuscript?	In which section is the information available? (Reagents and Tools Table, Materials and Methods, Figures, Data Availability Section)
Studies involving **human participants**: State details of **authority granting ethics approval** (IRB or equivalent committee(s), provide reference number for approval.	Not Applicable	
Studies involving **human participants**: Include a statement confirming that **informed consent** was obtained from all subjects and that the experiments conformed to the principles set out in the WMA Declaration of Helsinki and the Department of Health and Human Services Belmont Report.	Not Applicable	
Studies involving **human participants:** For publication of **patient photos**, include a statement confirming that consent to publish was obtained.	Not Applicable	
Studies involving experimental **animals**: State details of **authority granting ethics approva**l (IRB or equivalent committee(s), provide reference number for approval. Include a statement of compliance with ethical regulations.	Not Applicable	
Studies involving **specimen and field samples:** State if relevant **permits** obtained, provide details of authority approving study; if none were required, explain why.	Not Applicable	

Dual Use Research of Concern (DURC)	Information included in the manuscript?	In which section is the information available? (Reagents and Tools Table, Materials and Methods, Figures, Data Availability Section)
Could your study fall under dual use research restrictions? Please check biosecurity documents and list of **select agents and toxins** (CDC): https://www.selectagents.gov/sat/list.htm	Not Applicable	
If you used a select agent, is the security level of the lab appropriate and reported in the manuscript?	Not Applicable	
If a study is subject to dual use research of concern regulations, is the name of the **authority granting approval and reference number** for the regulatory approval provided in the manuscript?	Not Applicable	

Reporting

The MDAR framework recommends adoption of discipline-specific guidelines, established and endorsed through community initiatives. Journals have their own policy about requiring specific guidelines and recommendations to complement MDAR.

Adherence to community standards	Information included in the manuscript?	In which section is the information available? (Reagents and Tools Table, Materials and Methods, Figures, Data Availability Section)
State if relevant guidelines or checklists (e.g., **ICMJE, MIBBI, ARRIVE, PRISMA**) have been followed or provided.	Not Applicable	
For **tumor marker prognostic studies,** we recommend that you follow the **REMARK** reporting guidelines (see link list at top right). See author guidelines, under 'Reporting Guidelines'. Please confirm you have followed these guidelines.	Not Applicable	
For **phase II and III randomized controlled trials**, please refer to the **CONSORT** flow diagram (see link list at top right) and submit the CONSORT checklist (see link list at top right) with your submission. See author guidelines, under 'Reporting Guidelines'. Please confirm you have submitted this list.	Not Applicable	

Data Availability

Data availability	Information included in the manuscript?	In which section is the information available? (Reagents and Tools Table, Materials and Methods, Figures, Data Availability Section)
Have **primary datasets** been deposited according to the journal's guidelines (see 'Data Deposition' section) and the respective accession numbers provided in the Data Availability Section?	Yes	Data Availability Section
Were **human clinical and genomic datasets** deposited in a public access-controlled repository in accordance to ethical obligations to the patients and to the applicable consent agreement?	Not Applicable	
Are **computational models** that are central and integral to a study available without restrictions in a machine-readable form? Were the relevant accession numbers or links provided?	Not Applicable	
If publicly available data were reused, provide the respective **data citations in the reference list**.	Yes	References
